# Supplementary material for: Widespread signatures of natural selection across human complex traits and functional genomic categories
Source: Nat Commun. 2021 Feb 19;12:1164. doi: 10.1038/s41467-021-21446-3 (PMC7896067; doi:10.1038/s41467-021-21446-3)
Supplement: Supplementary file 1 — Supplementary Information [file 41467_2021_21446_MOESM1_ESM.pdf]

Supplementary Information

Supplementary Note 1: SBayesS model

Let us consider an individual-level data-based multiple regression model in a GWAS data set:

$$\mathbf{y} = \mathbf{X}\boldsymbol{\beta} + \mathbf{e} \tag{1}$$

where $\mathbf{y}$ is the vector of phenotypes adjusted for all fixed effects, $\mathbf{X}$ is the column-centered genotype matrix, $\boldsymbol{\beta}$ is the vector of SNP effects, and $\mathbf{e}$ is the vector of residuals with $Var(\mathbf{e}) = \mathbf{I}\sigma_e^2$ for a sample of unrelated individuals. Assuming Hardy-Weinberg equilibrium (HWE), the variance of the genotypes of SNP j is $h_j = 2p_jq_j$, where p_j is the minor allele frequency (MAF) and $q_j = 1 - p_j$. Let $\mathbf{D}$ be a diagonal matrix with $D_j = \mathbf{X}_j'\mathbf{X}_j = h_jn_j$, where n_j is per-SNP sample size. Multiplying both sides of (1) by $\mathbf{D}^{-1}\mathbf{X}'$ gives

$$\mathbf{D}^{-1}\mathbf{X}'\mathbf{y} = \mathbf{D}^{-1}\mathbf{X}'\mathbf{X}\boldsymbol{\beta} + \mathbf{D}^{-1}\mathbf{X}'\mathbf{e} \tag{2}$$

Note that $\mathbf{D}^{-1}\mathbf{X}'\mathbf{y} = \mathbf{b}$, the vector of least square estimates of SNP marginal effects from GWAS, and $\mathbf{D}^{-1}\mathbf{X}'\mathbf{X} = \mathbf{D}^{-\frac{1}{2}}\mathbf{B}\mathbf{D}^{\frac{1}{2}}$ where $\mathbf{B} = \mathbf{D}^{-\frac{1}{2}}\mathbf{X}'\mathbf{X}\mathbf{D}^{-\frac{1}{2}}$ is the linkage disequilibrium (LD) correlation matrix among all SNPs [1]. Let $\boldsymbol{\epsilon} = \mathbf{D}^{-1}\mathbf{X}'\mathbf{e}$. Then, (2) can be written as

$$\mathbf{b} = \mathbf{D}^{-\frac{1}{2}}\mathbf{B}\mathbf{D}^{\frac{1}{2}}\boldsymbol{\beta} + \boldsymbol{\epsilon} \tag{3}$$

Or, in a scalar form,

$$b_j = \sum_{k=1}^{m} \sqrt{\frac{h_kn_k}{h_jn_j}} B_{jk}\beta_k + \epsilon_j \tag{4}$$

with m being the total number of SNPs. Let $\sigma_{X_j}^2$, $\sigma_{X_k}^2$ and σ_{X_j,X_k} denote the genotype variance of SNP j and k and their covariance. Then, (4) can be simplified to

$$b_j = \sum_{k=1}^{m} \sqrt{\frac{n_k}{n_j}} \beta_{X_j,X_k}\beta_k + \epsilon_j \tag{5}$$

where $\beta_{X_j,X_k} = \sigma_{X_j,X_k}/\sigma_{X_j}^2$ is the regression of SNP k on that of SNP j. In other words, we model the marginal effect of each SNP as a linear combination of other SNP effects with the weights being a function of regression coefficient of SNP genotypes and per-SNP sample sizes. In contrast to the identity structure of residual variance in (1), the residuals in (3) are not independent in the presence of LD, because

$$\begin{aligned} Var(\boldsymbol{\epsilon}) &= Var\left(\mathbf{D}^{-1}\mathbf{X}'\mathbf{e}\right) \\ &= \mathbf{D}^{-1}\mathbf{X}'\mathbf{X}\mathbf{D}^{-1}\sigma_e^2 \\ &= \mathbf{D}^{-\frac{1}{2}}\mathbf{B}\mathbf{D}^{-\frac{1}{2}}\sigma_e^2 \end{aligned} \tag{6}$$

Let $\mathbf{W} = \mathbf{D}^{-\frac{1}{2}}\mathbf{B}\mathbf{D}^{\frac{1}{2}}$ and $\mathbf{R} = \mathbf{D}^{-\frac{1}{2}}\mathbf{B}\mathbf{D}^{-\frac{1}{2}}$. Finally, from (3) we have

$$\mathbf{b} = \mathbf{W}\boldsymbol{\beta} + \boldsymbol{\epsilon} \tag{7}$$

with $Var\left(\boldsymbol{\epsilon}\right) = \mathbf{R}\sigma_e^2$. This is a generic form of summary-data-based Bayesian regressions (SBR), which is similar to the RSS model of Zhu and Stephens [2].

As in BayesS [3], we assume the effect size is related to MAF through parameter S:

$$\beta_j \begin{cases} \sim N\left(0, h_j^S \sigma_\beta^2\right), & \pi \\ = 0, & 1-\pi \end{cases} \tag{8}$$

where S, σ_β^2 and π are considered as unknown. The prior for S is a standard normal distribution

$$S \sim N(0,1)$$

The prior for π is a uniform between zero and one, namely

$$\pi \sim Beta(1,1)$$

The prior for σ_β^2 is a scaled inverse chi-square distribution

$$\sigma_\beta^2 \sim \nu_\beta \tau_\beta^2 \chi_{\nu_\beta}^{-2}$$

where $\nu_\beta = 4$ and

$$\tau_\beta^2 = \frac{\nu_\beta - 2}{\nu_\beta} \frac{V_P h_0^2}{\pi_0 \sum_j h_j^{S_0+1}}$$

where V_P is the phenotypic variance estimated from the summary statistics (as shown below) and h_0^2, π_0 and S_0 are the prior knowledge of SNP-based heritability, π and S, resepctively. Similarly, we give a scaled inverse chi-square prior for σ_e^2 in (6)

$$\sigma_e^2 \sim \nu_e \tau_e^2 \chi_{\nu_e}^{-2}$$

where $\nu_e = 4$ and

$$\tau_e^2 = \frac{\nu_e - 2}{\nu_e} V_P \left(1 - h_0^2\right)$$

We estimate the phenotypic variance of the trait following Yang et al [1]'s approach, which is based on the stadard error of the marginal SNP effect estimate from GWAS. Because

$$SE_j^2 = \frac{\sigma_j^2}{\mathbf{X}_j'\mathbf{X}_j} = \frac{\mathbf{y}'\mathbf{y} - \mathbf{X}_j'\mathbf{X}_j b_j^2}{\mathbf{X}_j'\mathbf{X}_j}$$

where σ_j^2 is the residual variance for the GWAS model fitting SNP j. Rearranging gives

$$V_{P,j} = \frac{\mathbf{y}'\mathbf{y}}{n_j} = D_{jj} \left(SE_j^2 + \frac{b_j^2}{n_j} \right) \tag{9}$$

The phenotypic variance V_P is then calculated as the median of $V_{P,j}$ across all SNPs [1]. Since $D_{jj} = 2p_j q_j n_j$ but the allele frequencies from the publicly available summary data are often not exact, we substitute $V_{P,j}$ by V_P in (9) to reestimate p_j given the input values of SE_j, b_j and n_j.

We call model (7) with the above prior as "SBayesS". Specifying a different prior distribution to β_j gives SBR form of other Bayesian alphabet models. For example, a mixture prior of normals with different variances for β_j under SBR framework becomes SBayesR [4].

As shown above, when the LD correlations are computed using all SNPs in the GWAS sample, SBayesS model is a linear transformation of the BayesS model without loss of information, in which case the two models are equivalent in terms of posterior inference (Section). However, it is impractical to store pairwise LD correlations of all genome-wide SNPs in the computer memory and not always feasible to access individual-level genotypes of the GWAS sample. Thus, we propose to use a sparse LD matrix that is computed from a reference sample, ignoring the small LD correlation estimates due to sampling variation. In this case, SBayesS becomes an approximation to BayesS. Assuming the LD reference sample is a random draw from the same population of the GWAS sample, the discrepancy between SBayesS and BayesS arises from the sampling variance of LD correlations used in SBayesS. Ignoring the sampling variance of LD estimates may cause a failure to converge in the Markov chain Monte Carlo (MCMC) sampling process or a bias in parameter estimation (Section). In this study, we model analytically the sampling variance of LD estimates as part of the residual variance and allow the estimate of residual variance to vary across SNPs (Section). We show the MCMC sampling scheme for the model parameters in Section and an efficient updating stragety in MCMC in Section .

Equivalence between SBayesS and BayesS

Here, we show that when the LD correlations are computed using all SNPs in the GWAS sample, SBayesS and BayesS models are equivalent in terms of posterior inference. Without loss of generality we assume $\pi = 0$, the posterior distribution of $\boldsymbol{\beta}$ in SBayesS is

$$\begin{aligned}
f(\boldsymbol{\beta}|\,\mathbf{b}, else) &\propto f(\mathbf{b}|\,\boldsymbol{\beta}, else)\, f(\boldsymbol{\beta}) \\
&\propto \exp\left\{-\frac{1}{2\sigma_e^2}(\mathbf{b}-\mathbf{W}\boldsymbol{\beta})'\,\mathbf{R}^{-1}(\mathbf{b}-\mathbf{W}\boldsymbol{\beta})\right\}\exp\left\{-\frac{\boldsymbol{\beta}'\mathbf{G}^{-1}\boldsymbol{\beta}}{2\sigma_\beta^2}\right\} \\
&= \exp\left\{-\frac{1}{2\sigma_e^2}\left[\mathbf{b}'\mathbf{R}^{-1}\mathbf{b} - 2\boldsymbol{\beta}'\mathbf{W}'\mathbf{R}^{-1}\mathbf{b} + \boldsymbol{\beta}'\left(\mathbf{W}'\mathbf{R}^{-1}\mathbf{W} + \frac{\sigma_e^2}{\sigma_\beta^2}\mathbf{G}^{-1}\right)\boldsymbol{\beta}\right]\right\} \\
&\propto \exp\left\{-\frac{1}{2\sigma_e^2}\left[\boldsymbol{\beta}'\left(\mathbf{W}'\mathbf{R}^{-1}\mathbf{W} + \frac{\sigma_e^2}{\sigma_\beta^2}\mathbf{G}^{-1}\right)\boldsymbol{\beta} + 2\boldsymbol{\beta}'\mathbf{W}'\mathbf{R}^{-1}\mathbf{b}\right]\right\}
\end{aligned}$$

Note that

$$\begin{aligned}\mathbf{W}'\mathbf{R}^{-1}\mathbf{W} &= \mathbf{D}^{\frac{1}{2}}\mathbf{B}\mathbf{D}^{-\frac{1}{2}}\mathbf{D}^{\frac{1}{2}}\mathbf{B}^{-1}\mathbf{D}^{\frac{1}{2}}\mathbf{D}^{-\frac{1}{2}}\mathbf{B}\mathbf{D}^{\frac{1}{2}}\\ &= \mathbf{D}^{\frac{1}{2}}\mathbf{B}\mathbf{D}^{\frac{1}{2}}\\ &= \mathbf{D}^{\frac{1}{2}}\mathbf{D}^{-\frac{1}{2}}\mathbf{X}'\mathbf{X}\mathbf{D}^{-\frac{1}{2}}\mathbf{D}^{\frac{1}{2}}\\ &= \mathbf{X}'\mathbf{X}\end{aligned}$$

and

$$\begin{aligned}\mathbf{W}'\mathbf{R}^{-1}\mathbf{b} &= \mathbf{D}^{\frac{1}{2}}\mathbf{B}\mathbf{D}^{-\frac{1}{2}}\mathbf{D}^{\frac{1}{2}}\mathbf{B}^{-1}\mathbf{D}^{\frac{1}{2}}\mathbf{b}\\ &= \mathbf{D}\mathbf{b}\\ &= \mathbf{X}'\mathbf{y}\end{aligned}$$

Thus, the posterior distribution becomes

$$f\left(\boldsymbol{\beta}|\,\mathbf{b}, else\right) \propto \exp\left\{-\frac{1}{2\sigma_e^2}\left[\boldsymbol{\beta}'\left(\mathbf{X}'\mathbf{X}+\frac{\sigma_e^2}{\sigma_\beta^2}\mathbf{G}^{-1}\right)\boldsymbol{\beta}+2\boldsymbol{\beta}'\mathbf{X}'\mathbf{y}\right]\right\}$$

This is equivalent to the posterior distribution given the indivdiual genotype and phenotype data. It can be shown that the above is the kernel of a multivariate normal distribution, i.e.

$$\boldsymbol{\beta}|\,\mathbf{b}, else \sim MVN\left(\mathbf{C}^{-1}\mathbf{r}, \mathbf{C}^{-1}\sigma_{\mathbf{e}}^{\mathbf{2}}\right)$$

where $\mathbf{C} = \mathbf{W}'\mathbf{R}^{-1}\mathbf{W} + \frac{\sigma_e^2}{\sigma_\beta^2}\mathbf{G}^{-1} = \mathbf{X}'\mathbf{X} + \frac{\sigma_e^2}{\sigma_\beta^2}\mathbf{G}^{-1}$ and $\mathbf{r} = \mathbf{W}'\mathbf{R}^{-1}\mathbf{b} = \mathbf{X}'\mathbf{y}$. It is recognized that $\mathbf{C}$ and $\mathbf{r}$ are the left- and right-hand sides of the mixed-model equations

$$\underbrace{\left[\mathbf{W}'\mathbf{R}^{-1}\mathbf{W}+\mathbf{G}^{-1}\lambda\right]}_{\mathbf{C}}\boldsymbol{\beta} = \underbrace{\mathbf{W}'\mathbf{R}^{-1}\mathbf{b}}_{\mathbf{r}} \tag{10}$$

with $\lambda = \sigma_e^2/\sigma_\beta^2$. It can be further shown that in the Gibbs sampling, the full conditional distribution of β_j is

$$\beta_j\left|\mathbf{b}, \boldsymbol{\beta}_{-j}, else \sim N\left(\frac{r_j}{C_j}, \frac{\sigma_e^2}{C_j}\right)\right. \tag{11}$$

where

$$\begin{aligned}r_j &= D_j b_j - \sum_{k\neq j} D_j^{\frac{1}{2}} B_{jk} D_k^{\frac{1}{2}}\beta_k \qquad (12)\\ &= \mathbf{X}_j'\mathbf{y} - \sum_{k\neq j}\mathbf{X}_j'\mathbf{X}_k\beta_k\\ C_j &= D_j + \frac{\sigma_e^2}{h_j^S\sigma_\beta^2} = \mathbf{X}_j'\mathbf{X}_j + \frac{\sigma_e^2}{h_j^S\sigma_\beta^2}\end{aligned}$$

Consequence of ignoring LD sampling variation

The consequence of using a sparse LD matrix is that it can potentially bias the mean of the full conditional distribution for β_j. Let k denote a SNP in nonzero LD with the target SNP and l denote a SNP that does not have significant LD with the target SNP. The r_j in (12) can be written as

$$\begin{aligned} r_j &= D_j b_j - \sum_k D_j^{\frac{1}{2}} B_{jk} D_k^{\frac{1}{2}} \beta_k - \sum_l D_j^{\frac{1}{2}} B_{jl} D_l^{\frac{1}{2}} \beta_l \\ &= D_j b_j - \sum_k D_j^{\frac{1}{2}} B_{jk} D_k^{\frac{1}{2}} \beta_k - t_j \\ &= r_j^* - t_j \end{aligned} \tag{13}$$

The full conditional of β_j in (11) becomes

$$\beta_j | t_j \sim N\left(\frac{r_j^* - t_j}{C_j}, \frac{\sigma_e^2}{C_j}\right)$$

When reduced LD matrix is used, B_{jl} is set to be zero, we therefore completely ignore t_j and do not adjust the mean of the full conditional by the effects of SNPs in very low LD. However, although individual LD is trivial, the sum of them can be substantial after multiplied by n because $Var\left(\sum_l \mathbf{X}_j'\mathbf{X}_l\right) = D_j Var\left(\sum_l B_{jl}\right) \propto nm$ under the null. This may break the property of MCMC and fail the Gibbs sampling.

Modelling LD sampling variance

The use of a sparse LD matrix from a reference sample will result in two sources of sampling variation. The first is the difference in sampling variance between the reference and GWAS samples for the LD correlations included in the sparse LD matrix. The second is the sampling variance of LD correlations that are set to be zero. As shown above, ignoring these sampling variations will result in a bias of the mean in the full conditional distribution of β_j and thereby biases in the estimation of other model parameters. Here, we account for both sampling variations in the model, as described below.

Suppose the observed LD correlation between SNP j and k equal to the true population LD (ρ_{jk}) plus a deviation (tilde refers to the LD reference sample):

$$\begin{aligned} B_{jk} &= \rho_{jk} + \delta_{jk} \\ \widetilde{B}_{jk} &= \rho_{jk} + \widetilde{\delta}_{jk} \end{aligned}$$

Then, the LD correlation in the GWAS sample is

$$B_{jk} = \begin{cases} \widetilde{B}_{jk} + \left(\delta_{jk} - \widetilde{\delta}_{jk}\right) & \text{if } \rho_{jk} \neq 0 \\ \delta_{jk} & \text{if } \rho_{jk} = 0 \end{cases} \tag{14}$$

Let Δ_{jk} denote the unobserved quantity in (14), i.e.

$$\Delta_{jk} = \begin{cases} \delta_{jk} - \widetilde{\delta}_{jk} & \text{if } \rho_{jk} \neq 0 \\ \delta_{jk} & \text{if } \rho_{jk} = 0 \end{cases}$$

In (7), we can write

$$\mathbf{W} = \mathbf{W}^{+} + \mathbf{W}^{-} \tag{15}$$

where $\mathbf{W}^{+} = \mathbf{D}^{-\frac{1}{2}}\widetilde{\mathbf{B}}\mathbf{D}^{\frac{1}{2}}$ is the observed data, and $\mathbf{W}^{-} = \mathbf{D}^{-\frac{1}{2}}\boldsymbol{\Delta}\mathbf{D}^{\frac{1}{2}}$ is not observed. Substituing (15) in (7) give

$$\mathbf{b} = \mathbf{W}^{+}\boldsymbol{\beta} + \boldsymbol{\eta} \tag{16}$$

where $\boldsymbol{\eta} = \mathbf{W}^{-}\boldsymbol{\beta} + \boldsymbol{\epsilon}$ are the new residuals that contain difference in sampling deviation of LD between the GWAS and reference samples when the population LD is not zero, and sampling deviation of LD in the GWAS sample when the population LD is zero.

Conditional on $\boldsymbol{\Delta}$, the residual variance is

$$Var\left(\boldsymbol{\eta}\,|\boldsymbol{\Delta}\right) = \mathbf{W}^{-\prime}\mathbf{G}\mathbf{W}^{-}\sigma_{\beta}^{2} + \mathbf{R}\sigma_{e}^{2} \tag{17}$$

with $\mathbf{R} = \mathbf{D}^{-\frac{1}{2}}\widetilde{\mathbf{B}}\mathbf{D}^{-\frac{1}{2}}$. However, this cannot be computed because Δ is not observed.

Considering both $\boldsymbol{\beta}$ and $\boldsymbol{\Delta}$ as random, the diagonal value of $Var\left(\boldsymbol{\eta}\right)$ in 16 is

$$\begin{aligned}
Var\left(\eta_{j}\right) &= E\left[Var\left(\eta_{j}|\,\boldsymbol{\Delta}_{j}\right)\right] + Var\left[E\left(\eta_{j}|\,\boldsymbol{\Delta}_{j}\right)\right] \\
&= E\left[\left(\mathbf{W}_{j}^{-}\right)'\mathbf{G}\mathbf{W}_{j}^{-}\sigma_{\beta}^{2}\pi + D_{j}^{-1}\sigma_{e}^{2}\right] + 0 \\
&= E\left[\sum_{k=1}^{m} D_{j}^{-1}\Delta_{jk}^{2}D_{k}G_{k}\sigma_{\beta}^{2}\pi\right] + D_{j}^{-1}\sigma_{e}^{2} \\
&= D_{j}^{-1}\sigma_{\beta}^{2}\pi E\left[D_{k}G_{k}\right]E\left[\sum_{k}\Delta_{jk}^{2}\right] + D_{j}^{-1}\sigma_{e}^{2} \\
&= D_{j}^{-1}\sigma_{\beta}^{2}\pi\frac{\sum_{k}\left(2p_{k}q_{k}\right)^{S+1}n_{j}}{m}E\left[\sum_{k}\Delta_{jk}^{2}\right] + D_{j}^{-1}\sigma_{e}^{2} \\
&= D_{j}^{-1}\left(\pi\sum_{k}\left(2p_{k}q_{k}\right)^{S+1}\sigma_{\beta}^{2}\right)\frac{n_{j}}{m}E\left[\sum_{k}\Delta_{jk}^{2}\right] + D_{j}^{-1}\sigma_{e}^{2} \\
&= D_{j}^{-1}\sigma_{g}^{2}\frac{n_{j}}{m}E\left[\sum_{k}\Delta_{jk}^{2}\right] + D_{j}^{-1}\sigma_{e}^{2} \\
&= D_{j}^{-1}\left(\frac{n_{j}}{m}\sum_{k}E\left[\Delta_{jk}^{2}\right]\sigma_{g}^{2} + \sigma_{e}^{2}\right) \\
&= D_{j}^{-1}\left(\frac{n_{j}}{m}\sum_{k}Var\left(\Delta_{jk}\right)\sigma_{g}^{2} + \sigma_{e}^{2}\right)
\end{aligned} \tag{18}$$

When $\rho_{jk} \neq 0$,

$$\begin{aligned} Var\left(\Delta_{jk}\right) &= Var\left(\delta_{jk}\right) + Var\left(\widetilde{\delta}_{jk}\right) - 2Cov\left(\delta_{jk}, \widetilde{\delta}_{jk}\right) \\ &= \frac{\left(1-\rho_{jk}^2\right)^2}{n_j} + \frac{\left(1-\rho_{jk}^2\right)^2}{\widetilde{n}_j} - 2Cov\left(\delta_{jk}, \widetilde{\delta}_{jk}\right) \end{aligned} \tag{19}$$

If there is no sample overlap between the LD reference and GWAS samples, then $Cov\left(\delta_{jk}, \widetilde{\delta}_{jk}\right) = 0$. If we compute LD from the GWAS sample itself, then $Var\left(\Delta_{jk}\right) = 0$.

When $\rho_{jk} = 0$,

$$Var\left(\Delta_{jk}\right) = Var\left(\delta_{jk}\right) = \frac{1}{n_j}$$

If we estimate ρ_{jk} by $\widetilde{B}_{jk}$ and assume there is no sample overlap between the LD reference and GWAS samples, substituting these results in (18) gives

$$Var\left(\eta_j\right) = D_j^{-1}\left[\left(\frac{n_j}{m}s_j^2 + \frac{m_j^0}{m}\right)\sigma_g^2 + \sigma_e^2\right] \tag{20}$$

where m_j^0 is the number of SNPs not in population LD with SNP j, σ_g^2 is the trait genetic variance, and

$$s_j^2 = \sum_{k=1}^{m_j}\left[\frac{\left(1-\rho_{jk}^2\right)^2}{n_j} + \frac{\left(1-\rho_{jk}^2\right)^2}{\widetilde{n}_j} - 2Cov\left(\delta_{jk}, \widetilde{\delta}_{jk}\right)\right]$$

is the total sampling variance for non-zero LD. In practice, we approximate ρ_{jk} by $\widetilde{B}_{jk}$. In the absence of sample overlap between the LD reference and GWAS samples, $Cov\left(\delta_{jk}, \widetilde{\delta}_{jk}\right) = 0$. In the case of complete sample overlap, $s_j^2 = 0$.

Similarly, considering both $\boldsymbol{\beta}$ and $\boldsymbol{\Delta}$ as random, the off-diagonal value of $Var\left(\boldsymbol{\eta}\right)$ in 16 is

$$\begin{aligned} Cov\left(\eta_j, \eta_k\right) &= E\left[Cov\left(\eta_j, \eta_k \middle| \boldsymbol{\Delta}_j, \boldsymbol{\Delta}_k\right)\right] + Cov\left[E\left(\eta_j \middle| \boldsymbol{\Delta}_j\right), E\left(\eta_k \middle| \boldsymbol{\Delta}_k\right)\right] \\ &= E\left[\left(\mathbf{W}_j^-\right)' \mathbf{G}\mathbf{W}_k^- \sigma_\beta^2 + D_j^{-\frac{1}{2}}\widetilde{B}_{jk}D_k^{-\frac{1}{2}}\sigma_e^2\right] + 0 \\ &= E\left[\sum_l D_j^{-\frac{1}{2}}D_k^{-\frac{1}{2}}\Delta_{jl}\Delta_{kl}D_lG_l\sigma_\beta^2\pi\right] + D_j^{-\frac{1}{2}}\widetilde{B}_{jk}D_k^{-\frac{1}{2}}\sigma_e^2 \\ &= 0 + D_j^{-\frac{1}{2}}\widetilde{B}_{jk}D_k^{-\frac{1}{2}}\sigma_e^2 \\ &= R_{jk}\sigma_e^2 \end{aligned}$$

Thus, the sampling variance of LD correlations only affect the diagonal but not off-diagonal values of the residual variance.

According to the derivation above, we have the following observations:

1. The LD sampling variance only affects the variance but not covariance of the model residuals. Thus, accounting for the LD sampling variance in the Gibbs sampling of β_j is straightforward (see below).

2. The LD sampling variation has two components, one due to the use of a different reference sample for LD information and the other due to the use of a sparse LD matrix, both of which are proportional to the genetic variance. If LD are estimated from the GWAS sample, $Var(\eta_j) = D_j^{-1}\left(\frac{m_j^0}{m}\sigma_g^2 + \sigma_e^2\right)$. Further, if the genome-wide full LD matrix is used, $Var(\eta_j) = D_j^{-1}\sigma_e^2$, the same as that in (6).

3. If SNP j is independent of all other SNPs, $m_j^0 = m-1$ and $s_j^2 = 0$. Therefore, $Var(\eta_j) = D_j^{-1}\left(\frac{m-1}{m}\sigma_g^2 + \sigma_e^2\right) = D_j^{-1}\left(\sigma_y^2 - \sigma_j^2\right) = D_j^{-1}\left(y^{'}y - D_j\sigma_\beta^2\right)/n_j$, which is the residual variance under a single-SNP GWAS model.

4. Under some conditions, e.g., small $\tilde{n}_j$ but large n_j, s_j^2 can be greater than 1. Thus, in the presence of LD sampling variance, the total residual variance (in the square brackets of (20)) can be greater than the phenotypic variance of the trait.

MCMC sampling scheme

The joint distribution of the data and parameters in model (7) is

$$\begin{aligned} f\left(\mathbf{b},\boldsymbol{\beta},\pi,S,\sigma_\beta^2,\sigma_e^2\right) \propto\ & \left|\mathbf{R}\sigma_e^2\right|^{-\frac{1}{2}}\exp\left\{-\frac{1}{2\sigma_e^2}\left(\mathbf{b}-\mathbf{W}\boldsymbol{\beta}\right)'\mathbf{R}^{-1}\left(\mathbf{b}-\mathbf{W}\boldsymbol{\beta}\right)'\right\} \\ \times\ & \prod_{j=1}^{m}\left[\left(h_j^S\sigma_\beta^2\right)^{-\frac{1}{2}}\exp\left\{-\frac{\beta_j^2}{2h_j^S\sigma_\beta^2}\right\}\pi+\phi\left(1-\pi\right)\right] \\ \times\ & \exp\left\{-\frac{S^2}{2}\right\} \\ \times\ & \left(\sigma_\beta^2\right)^{-\frac{2+\nu_\beta}{2}}\exp\left\{-\frac{\nu_\beta\tau_\beta^2}{2\sigma_\beta^2}\right\} \\ \times\ & \left(\sigma_e^2\right)^{-\frac{2+\nu_e}{2}}\exp\left\{-\frac{\nu_e\tau_e^2}{2\sigma_e^2}\right\} \end{aligned}$$

To obtain a joint posterior sample for parameter inference, we iteratively sample each parameter from its full conditional distribution. Except S, the full conditional distribution has a closed form for all the parameters, as shown below.

To deal with the mixture prior for β_j, we introduce an indicator variable δ_j

$$\delta_j \sim Bernoulli(\pi)$$

such that

$$\beta_j \begin{cases} \sim N(0, h_j^S\sigma_\beta^2), & \delta_j = 1 \\ = 0, & \delta_j = 0 \end{cases}$$

We first sample δ_j unconditional on β_j and then sample β_j conditional on δ_j, which has been shown to have slightly better mixing. The full conditional distribution for δ_j is

$$\delta_j\left|\mathbf{b}, else\right. \sim Bernoulli(\hat{\pi})$$

where

$$\hat{\pi} = \frac{f(\mathbf{b}\,|\delta_j = 1, else)\,\pi}{f(\mathbf{b}\,|\delta_j = 1, else)\,\pi + f(\mathbf{b}\,|\delta_j = 0, else)\,(1-\pi)}$$
$$= \left[1 + \frac{f(\mathbf{b}\,|\delta_j = 0, else)}{f(\mathbf{b}\,|\delta_j = 1, else)}\frac{1-\pi}{\pi}\right]^{-1} \quad (21)$$

It is obvious that

$$f(\mathbf{b}\,|\delta_j = 0, else) = \left(2\pi\,|\mathbf{R}|\,\sigma_e^2\right)^{-\frac{1}{2}} \exp\left\{-\frac{1}{2\sigma_e^2}\mathbf{b}'_{adj}\mathbf{R}^{-1}\mathbf{b}_{adj}\right\} \quad (22)$$

where $\mathbf{b}_{adj} = \mathbf{b} - \sum_{k\neq j}\mathbf{W}_k\beta_k$ is the adjusted $\mathbf{b}$ for all the other SNP effects except SNP j. Fortunately, we do not need to compute this quantity because it will be cancelled in the likelihood ratio in (21), as shown below. For $f(\mathbf{b}\,|\delta_j = 1, else)$, to be unconditional on β_j, we compute

$$f(\mathbf{b}\,|\delta_j = 1, else) = \int f(\mathbf{b}\,|\delta_j = 1, \beta_j, else)\, f(\beta_j)\, d\beta_j$$
$$= \left(2\pi\,|\mathbf{R}|\,\sigma_e^2\right)^{-\frac{1}{2}} \exp\left\{-\frac{1}{2\sigma_e^2}\mathbf{b}'_{adj}\mathbf{R}^{-1}\mathbf{b}_{adj}\right\}\left(\frac{\sigma_{e_j}^{2*}}{h_j^S\sigma_\beta^2 C_j^*}\right)^{\frac{1}{2}} \exp\left\{\frac{\left(r_j^*\right)^2}{2C_j^*\sigma_{e_j}^{2*}}\right\} \quad (23)$$

This equation is derived based on integrating β_j out of the joint distribution of $\mathbf{b}$ and β_j, which is closely related to the full conditional distribution of β_j, as shown next. Also see below for the definition of $\sigma_{e_j}^{2*}$, C_j^* and r_j^*. Substituting (22) and (23) into (21) gives the full conditional probability for $\delta_j = 1$.

Let $\sigma_{e_j}^{2*} = \left(\frac{n_j}{m}s_j^2 + \frac{m_j^0}{m}\right)\sigma_g^2 + \sigma_e^2$, which explicitly models the sampling variation of LD as described above. The full conditional distribution of β_j (11) is

$$\beta_j\,|\mathbf{b}, \boldsymbol{\beta}_{-j}, else \sim N\left(\frac{r_j^*}{C_j^*}, \frac{\sigma_{e_j}^{2*}}{C_j^*}\right) \quad (24)$$

with r_j^* as in (13) (ignores t_j the cumulative effects of SNPs in chance LD) and $C_j^* = D_j + \frac{\sigma_{e_j}^{2*}}{h_j^S\sigma_\beta^2}$. It can be seen that instead of adjusting for the "leftover" effect from the mean, we shrink the mean towards zero while increase the variance (uncertainty) of the posterior distribution, because $\sigma_{e_j}^{2*}/C_j^* = 1/\left(D_j/\sigma_{e_j}^{2*} + 1/h_j^S\sigma_\beta^2\right)$.

Given the sampled values of $\boldsymbol{\delta}$ and $\boldsymbol{\beta}$, the full conditional distribution for π, σ_β^2 and S is the same as in BayesS, with the sampling procedure elaborated in the Supplementary Note of Zeng et al [3].

The full conditional distribution for the residual variance σ_e^2 is

$$\sigma_e^2\,|\mathbf{b}, else \sim \nu_e\tau_e^2\chi_{\nu_e}^{-2} \quad (25)$$

where $\nu_e = \bar{n} + \nu_{e_0}$ and $\tau_e^2 = \left(\mathbf{e}'\mathbf{e} + \nu_{e_0}\tau_{e_0}^2\right)/\nu_e$ with ν_{e_0} and $\tau_{e_0}^2$ being the prior values. The residual sum of squares ($\mathbf{e}'\mathbf{e}$) can be computed as

$$\begin{aligned}\mathbf{e}'\mathbf{e} &= (\mathbf{y}-\mathbf{X}\boldsymbol{\beta})'(\mathbf{y}-\mathbf{X}\boldsymbol{\beta}) \\ &= \mathbf{y}'\mathbf{y}-2\boldsymbol{\beta}'\mathbf{X}'\mathbf{y}+\boldsymbol{\beta}'\mathbf{X}'\mathbf{X}\boldsymbol{\beta} \\ &= \mathbf{y}'\mathbf{y}-2\boldsymbol{\beta}'\mathbf{r}+\boldsymbol{\beta}'(\mathbf{r}-\mathbf{r}_{adj}) \\ &= \mathbf{y}'\mathbf{y}-\boldsymbol{\beta}'\mathbf{r}-\boldsymbol{\beta}'\mathbf{r}_{adj} \end{aligned} \tag{26}$$

where $\mathbf{r}=\mathbf{Db}$ and $\mathbf{r}_{adj}=\mathbf{r}-\mathbf{X}'\mathbf{X}\boldsymbol{\beta}$ is the adjusted right-hand-side from the right-hand-side updating strategy (see below). The total sum of squares $\mathbf{y}'\mathbf{y}$ is computed from (9) and the median is used as the estimate of $\mathbf{y}'\mathbf{y}$ in (26).

The right-hand-side updating strategy and parallel computing

The MCMC implementation requires a computation of r_j in (12) for $m \times t$ times where m is the number of SNPs and t is the number of MCMC iterations. This is where the most majority computing time is spent. It can be seen from (12) that the summary-data level model is already much more efficient than the individual-data level model, because the vector-by-vector products $\mathbf{X}_j'\mathbf{y}$ and $\mathbf{X}_j'\mathbf{X}_k$ are replaced by scalar products $D_j b_j$ and $D_j^{\frac{1}{2}} B_{jk} D_k^{\frac{1}{2}}$. However, the adjustment of $D_j b_j$ (the right-hand-side $\mathbf{r}$ of the mixed-model equations (10)) for all the other SNP effects is still too computationally intense given over a million of SNPs. To improve computational efficiency, we adopted the so-called right-hand-side updating algorithm for genomic prediction in the context of animal breeding [5]. We set out to compute a vector of adjusted right-hand-side

$$\mathbf{r}_{adj}=\mathbf{r}-\mathbf{C}\boldsymbol{\beta}$$

, where $\mathbf{r}=\mathbf{Db}$ and $\mathbf{C}=\mathbf{D}^{\frac{1}{2}}\mathbf{B}\mathbf{D}^{\frac{1}{2}}$ (note that here $\mathbf{C}$ is defined different from that in (10)). For each SNP, we compute

$$r_j = r_{adj,j} + C_{jj}\beta_j$$

and use r_j in the full conditional distribution of β_j (24). After a new value of β_j is sampled, we update the adjusted right-hand-side

$$\mathbf{r}_{adj}^{new}=\mathbf{r}_{adj}^{old}+\mathbf{C}_j\left(\beta_j^{old}-\beta_j^{new}\right)$$

The benefit of this updating strategy comes from three sides. First, the computation of r_j for each SNP becomes trivial (reducing from vector to scalar operation). Second, the vector of $\mathbf{r}_{adj}$ needs to be updated only when either β_j^{old} or β_j^{new} is not zero, thus the sparse genetic architecture will lead to a substantial gain in speed. Third, due to the use of a sparse LD matrix, the vector of $\mathbf{C}_j$ has a large proportion of zero and therefore only a small fraction of $\mathbf{r}_{adj}$ according to nonzero $\mathbf{C}_j$ elements needs to be updated. In summary, the right-hand-side updating strategy substantially improves computational efficiency by taking the advantages of the sparse genetic architecture and the sparse LD correlation matrix. We further improve the efficiency by implementing a parallel

computing for sampling β_j of SNPs located on different chromosomes, and then combine results across threads to estimate the global parameters such as π, σ_β^2, S and etc. This led to about 4 times faster when 4 cores were used with OpenMP library in our real trait analysis.

Algorithm pseudocode

Our SBayesS algorithm is implemented in GCTB based on the following pseudocode:

Algorithm 1 SBayesS algorithm

Initialise parameters and read summary statistics
Reconstruct $\mathbf{X'X}$ and $\mathbf{X'y}$ from summary statistics and LD reference panel ($\mathbf{D}$ = diagonal elements of $\mathbf{X'X}$)
Calculate $\mathbf{r}^* = \mathbf{X'y} - \mathbf{X'X}\boldsymbol{\beta}$
for $i = 1$ to number of iterations **do**
  for $j = 1$ to number of SNPs **do**
    Calculate $\sigma_{e_j}^{2*} = \left(\frac{n_j}{m} s_j^2 + \frac{m_j^0}{m}\right) \sigma_g^2 + \sigma_e^2$
    Calculate the right hand side $r_j = r_j^* - D_j \beta_j$
    Calculate the left hand side $C_j = D_j + \frac{\sigma_{e_j}^{2*}}{(2p_j q_j)^S \sigma_\beta^2}$
    Calculate the posterior probability for $\delta_j = 1$ with $\Pr(\delta_j = 1|\mathbf{b}, \boldsymbol{\theta}) = \frac{1}{\exp[\log(L_0) - \log(L_1)]}$, where $\log(L_0) - \log(L_1) = \log(1-\pi) - 0.5(\log(C_j^{-1}) - \log((2p_j q_j)^S \sigma_\beta^2) + C_j^{-1} r_j^2) - \log\pi$
    Sample δ_j based on the posterior probability
    if $\delta = 1$ **then**
      Sample β_j from the full conditional distribution $N(\frac{r_j}{C_j}, \frac{\sigma_{e_j}^{2*}}{C_j})$
      Update $\mathbf{r}^* = \mathbf{r}^* + \mathbf{X'X}_j(\beta_j^{(i-1)} - \beta_j)$, where $\mathbf{X'X}_j$ is the $j^t h$ column of $\mathbf{X'X}$ and $\beta_j^{(i-1)}$ is the sampled value from the last iteration
    else
      if $\beta_j^{(i-1)} = 0$ **then**
        Update $\mathbf{r}^* = \mathbf{r}^* + \mathbf{X'X}_j \beta_j^{(i-1)}$
      end if
    end if
  end for
  Sample σ_β^2 from the full conditional distribution $\nu_\beta \tau_\beta^2 \chi_{\nu_\beta}^{-2}$ with $\nu_\beta = m_{nz} + \nu_{\beta_0}$ and $\tau_\beta = \frac{\sum \frac{\beta_j^2}{(2p_j q_j)^S} + \nu_{\beta_0}\tau_{\beta_0})}{\nu_\beta}$
  Sample S from the full conditional distribution using HMC algorithm
  Sample π from the full conditional distribution $Beta(m_{NZ} + a_0, m - m_{NZ} + b_0)$
  Sample σ_e^2 from the full conditional distribution $\nu_e \tau_e^2 \chi_{\nu_e}^{-2}$, where $\nu_e = \bar{n} + \nu_{e_0}$ and $\tau_e^2 = \frac{(\mathbf{e'e} + \nu_{e_0}\tau_{e_0}^2)}{\nu_e}$ with $\mathbf{e'e} = \mathbf{y'y} - \boldsymbol{\beta}'\mathbf{X'y} - \boldsymbol{\beta}'\mathbf{r}^*$
  Calculate the total genetic variance $\sigma_g^2 = \frac{\boldsymbol{\beta}'\mathbf{X'y} - \boldsymbol{\beta}'\mathbf{r}^*}{\bar{n}}$
  Calculate the SNP-based heritability $h_{SNP}^2 = \frac{\sigma_g^2}{\sigma_g^2 + \sigma_e^2}$
end for

Supplementary Note 2: Our genetic architecture parameter estimators are subject to SNP set and sample size

SNP-based heritability (h_{SNP}^2), polygenicity (π) and the relationship between MAF and effect size (S), all of which are defined with respect to a certain set of SNPs (see the definition of h_{SNP}^2 as an example [6]). Because the estimators for the genetic architecture parameters are based on the SNP markers rather than the actual causal variants, the estimates therefore rely on the tagging of the SNPs to the causal variants. For example,

our polygenicity estimate will be lower than that at the causal variants if many causal variants are not observed and poorly tagged by the SNPs (as shown in Supplementary Figure 4 in Zeng et al. [3]). In addition to the SNP panel, sample size can also affect our parameter estimates. As sample size increases, the polygenicity estimate is expected to increase because of the increased power to detect nonnull SNPs with very small effect sizes until all the genetic effects are detected. The other parameters, h^2_{SNP} and S, are estimated based on the nonnull SNPs, therefore the estimators of these parameters are also SNP set and sample size dependent.

Despite the expected differences, the parameter estimates from the real traits were by-and-large consistent between different GWAS sample sizes. We randomly sampled 120k individuals from the UKBv3 dataset, performed GWAS and ran SBayesS. The correlation was 0.99 for SNP-heritability, 0.95 for polygenicity and 0.81 for the S parameter (Supplementary Fig. 6c). Compared to SNP-based heritability and polygenicity, the correlation for the S parameter was lower. In addition to the reason that S depends on the SNPs that are identified with nonzero effects and the number of which was larger with N=350k, the lower correlation for S was also because the S parameter is a hyper-parameter, which is more difficult to estimate and thereby estimated with a higher degree of uncertainly (i.e., a larger standard error).

To further investigate the property of our estimators, we performed a simulation based on the real trait results. That is, for each of the 18 traits in our benchmarking analysis, we simulated a trait using the estimated h^2_{SNP}, π and S as the true values for the heritability, the proportion of causal variants and the S at the causal variants. Then, we ran SBayesS with the GWAS summary statistics computed with $n = 350$K or 120K, excluding the causal variants in the SNP panel. Supplementary Fig. 7a shows that $\widehat{h}^2_{SNP}$ was slightly lower than the heritability at the causal variants (because of imperfect tagging), $\widehat{\pi}$ is slightly higher than the proportion of causal variants (because of multiple SNPs jointly capturing a causal variant), and $\widehat{S}$ is almost unbiased. Supplementary Fig. 7b shows that the estimates with different sample sizes are highly concordant when the SNPs have a good tagging ability (in the simulation, we sampled causal variants from the 1.1 million common SNPs and left out the causal variants). This suggests that the additional variation and mean difference observed in the real data analysis (Supplementary Fig. 6c) are likely to be due to the insufficient tagging of HapMap3 common SNPs to the unobserved causal variants.

Finally, we ran SBayesS-strat with function annotations using the down-sampled dataset, and found a good consistency for different sample sizes (correlation $r = 0.99$ for h^2_{SNP} enrichment, $r = 0.93$ for polygenicity enrichment, $r = 0.87$ for h^2_{NZE} enrichment and $r = 0.73$ for the estimated S; Supplementary Figure 31). Given a higher degree of uncertainty (a larger standard error), the S parameter estimates with the two different sample sizes were reasonably consistent, although the correlation for the S parameter was lower than that for h^2_{SNP} and polygenicity as explained above. Compared to the per trait estimate (Supplementary Fig. 6c), the polygenicity enrichment estimates in the stratified model were much less sensitive to sample size because the extra number of small effects detected by a larger sample size in a functional category is likely proportional to that in the whole genome and therefore cancelled out in the ratio.

Supplementary Note 3: Simulation of GWAS summary statistics

The objective is to simulate the GWAS summary statistics $\widehat{\mathbf{b}}$ and their stanadard errors with an arbitrary sample size n given a reference sample. Consider model

$$\mathbf{y} = \mathbf{X}\boldsymbol{\beta} + \mathbf{e}$$

Let $\mathbf{D}$ be a diagonal matrix with $D_j = 2p_j q_j n_j$. The genotype matrix $\mathbf{X}$ is centered but not standardised.

$$\mathbf{D}^{-1}\mathbf{X}'\mathbf{y} = \mathbf{D}^{-1}\mathbf{X}'\mathbf{X}\boldsymbol{\beta} + \mathbf{D}^{-1}\mathbf{X}'\mathbf{e}$$

Let $\mathbf{B}$ be the LD correlation matrix.

$$\begin{aligned}\widehat{\mathbf{b}} &= \mathbf{D}^{-\frac{1}{2}}\mathbf{B}\mathbf{D}^{\frac{1}{2}}\boldsymbol{\beta} + \mathbf{D}^{-1}\mathbf{X}'\mathbf{e} \\ &= \mathbf{b} + \boldsymbol{\epsilon}\end{aligned}$$

Assuming normality,

$$\widehat{\mathbf{b}} \sim N\left(\mathbf{D}^{-\frac{1}{2}}\mathbf{B}\mathbf{D}^{\frac{1}{2}}\boldsymbol{\beta}, \mathbf{D}^{-\frac{1}{2}}\mathbf{B}\mathbf{D}^{-\frac{1}{2}}\sigma_{\epsilon}^{\mathbf{2}}\right)$$

The LD matrix $\mathbf{B}$ is estimated from the reference sample of a relatively small sample size. In order to remove chance LD and facilitate computation, we follow the method in O'Connor and Price [7] to convert the full matrix into a positive semidefinite block diagonal matrix. The first step is to convert the full matrix into a block diagonal matrix with 50 blocks, each containing a LD window size of 2Mb (the total chromosome length is 100Mb in the simulation). The next step is to perform an eigen decomposition on each block:

$$\mathbf{B}_i = \mathbf{V}_i\boldsymbol{\Sigma}_i\mathbf{V}_i'$$

To remove noise and ensure a positive definite LD matrix, we removed the non-positive eigenvalues in $\boldsymbol{\Sigma}_i$ and the corresponding eigenvectors in $\mathbf{V}_i$. The last step is to re-normalize each block to have the diagonal values equal to one:

$$\mathbf{B}_i^* = \mathbf{J}_i^{-\frac{1}{2}}\mathbf{B}_i\mathbf{J}_i^{-\frac{1}{2}}$$

where $\mathbf{J}_i$ is the diagonal matrix corresponding to the diagnal of $\mathbf{B}_i$.

Thus,

$$\mathbf{b} = \mathbf{D}^{-\frac{1}{2}}\mathbf{B}^*\mathbf{D}^{\frac{1}{2}}\boldsymbol{\beta}$$

Since

$$\mathbf{B}_i^* = \mathbf{J}_i^{-\frac{1}{2}}\mathbf{V}_i\boldsymbol{\Sigma}_i^{\frac{1}{2}}\boldsymbol{\Sigma}_i^{\frac{1}{2}}\mathbf{V}_i'\mathbf{J}_i^{-\frac{1}{2}}$$

let $u \sim N(0,1)$, then

$$Var\left(\mathbf{D}_i^{-\frac{1}{2}}\mathbf{J}_i^{-\frac{1}{2}}\mathbf{V}_i\mathbf{\Sigma}_i^{\frac{1}{2}}\mathbf{u}_i\sigma_\epsilon\right) = \mathbf{D}_i^{-\frac{1}{2}}\mathbf{B}_i^*\mathbf{D}_i^{-\frac{1}{2}}\sigma_\epsilon^2$$

Thus,

$$\boldsymbol{\epsilon}_i = \mathbf{D}_i^{-\frac{1}{2}}\mathbf{J}_i^{-\frac{1}{2}}\mathbf{V}_i\mathbf{\Sigma}_i^{\frac{1}{2}}\mathbf{u}_i\sigma_\epsilon$$

where $\sigma_\epsilon^2 \approx \sigma_y^2$. The standard error of $\widehat{b}_j$ (SE) is the square root of

$$Var\left(\widehat{b}_j\right) = D_j^{-1}\sigma_{\epsilon,j}^2 = D_j^{-1}\left(\sigma_y^2 - 2p_jq_j\widehat{b}_j^2\right)$$

Supplementary Note 4: Maximum likelihood estimation of S

Here, we derive the maximum likelihood estimate (MLE) of S given the true effect sizes ($\boldsymbol{\beta}$) of m causal variants. Since our model is

$$\beta_j \sim N\left(0, h_j^S\sigma_\beta^2\right)$$

where $h_j = 2p_j(1-p_j)$, the likelihood function is

$$f\left(\boldsymbol{\beta}|\, S, \sigma_\beta^2\right) = \prod_{j=1}^{m}(2\pi)^{-\frac{1}{2}}\left(h_j^S\sigma_\beta^2\right)^{-\frac{1}{2}}\exp\left\{-\frac{\beta_j^2}{2h_j^S\sigma_\beta^2}\right\}$$

Thus, the log-likelihood function, after dropping out the normalising constant, is

$$\mathcal{L} = -\frac{S}{2}\sum_{j=1}^{m}\log h_j - \frac{m}{2}\log\sigma_\beta^2 - \frac{1}{2\sigma_\beta^2}\sum_{j=1}^{m}\frac{\beta_j^2}{h_j^S}$$

Taking the partial derivatives of $\mathcal{L}$ with respect to S and σ_β^2, respectively, gives

$$\frac{\partial\mathcal{L}}{\partial S} = -\frac{1}{2}\sum_{j=1}^{m}\log h_j + \frac{1}{2\sigma_\beta^2}\sum_{j=1}^{m}\left(\frac{\beta_j^2}{h_j^S}\log h_j\right)$$

$$\frac{\partial\mathcal{L}}{\partial\sigma_\beta^2} = -\frac{m}{2\sigma_\beta^2} + \frac{1}{2\left(\sigma_\beta^2\right)^2}\sum_{j=1}^{m}\frac{\beta_j^2}{h_j^S}$$

The MLE of S and σ_β^2 can be obtained by solving the above equations that set to be zero. In the simulation study, we used *optim* function in R to find the MLE of S.

Supplementary Note 5: Standard error of predicted evolutionary parameter

We estimated the standard eror of predicted evolutionary parameter by considering a random coefficient linear model. Our model for predicting an evolutionary parameter is: $\widehat{\theta} = \widehat{\mathbf{X}}'\widehat{\boldsymbol{\beta}}$ where $\widehat{\mathbf{X}}$ is a vector of the three genetic architecture parameter estimates from SBayesS and their respective polynomial terms and $\widehat{\boldsymbol{\beta}}$ is a vector of the

weights estimated from the reference dataset of simulation. There are two sources of estimation uncertainty. The first one is the uncertainty in estimating $\widehat{\mathbf{X}}$ by SBayesS. The second source is the uncertainty in estimating $\widehat{\boldsymbol{\beta}}$ by the polynomial regression in training. The estimation variance of $\widehat{\theta}$ can be therefore written as

$$\begin{aligned}
Var\left(\widehat{\mathbf{X}}'\widehat{\boldsymbol{\beta}}\right) &= Var_{\widehat{\beta}}\left[E_{\widehat{X}}\left(\widehat{\mathbf{X}}'\widehat{\boldsymbol{\beta}}\middle|\widehat{\boldsymbol{\beta}}\right)\right] + E_{\widehat{\beta}}\left[Var_{\widehat{X}}\left(\widehat{\mathbf{X}}'\widehat{\boldsymbol{\beta}}\middle|\widehat{\boldsymbol{\beta}}\right)\right] \\
&= Var_{\widehat{\beta}}\left[\mathbf{X}'\widehat{\boldsymbol{\beta}}\right] + E_{\widehat{\beta}}\left[\widehat{\boldsymbol{\beta}}'Var\left(\widehat{\mathbf{X}}\right)\widehat{\boldsymbol{\beta}}\right] \\
&= \mathbf{X}'Var\left(\widehat{\boldsymbol{\beta}}\right)\mathbf{X} + E_{\widehat{\beta}}\left[tr\left(\widehat{\boldsymbol{\beta}}'Var\left(\widehat{\mathbf{X}}\right)\widehat{\boldsymbol{\beta}}\right)\right] \\
&= \mathbf{X}'Var\left(\widehat{\boldsymbol{\beta}}\right)\mathbf{X} + E_{\widehat{\beta}}\left[tr\left(\widehat{\boldsymbol{\beta}}\widehat{\boldsymbol{\beta}}'Var\left(\widehat{\mathbf{X}}\right)\right)\right] \\
&= \mathbf{X}'Var\left(\widehat{\boldsymbol{\beta}}\right)\mathbf{X} + tr\left[E\left(\widehat{\boldsymbol{\beta}}\widehat{\boldsymbol{\beta}}'\right)Var\left(\widehat{\mathbf{X}}\right)\right] \\
&= \mathbf{X}'Var\left(\widehat{\boldsymbol{\beta}}\right)\mathbf{X} + tr\left[Var\left(\widehat{\boldsymbol{\beta}}\right)Var\left(\widehat{\mathbf{X}}\right)\right]
\end{aligned}$$

where $Var\left(\widehat{\mathbf{X}}\right)$ is the variance-covariance matrix of the posterior estimates computed from the MCMC samples of SBayesS and $Var\left(\widehat{\boldsymbol{\beta}}\right)$ is the estimation variance matrix of the weights obtained from the polynomial regression in the reference dataset. The true values of $\mathbf{X}$ are unknown and therefore we replace them by their estimates $\widehat{\mathbf{X}}$.

Supplementary Note 6: Acknowledgements

UKB: This study has been conducted using UK Biobank resource under Application Number 12505. UK Biobank was established by the Wellcome Trust medical charity, Medical Research Council, Department of Health, Scottish Government and the Northwest Regional Development Agency. It has also had funding from the Welsh Assembly Government, British Heart Foundation and Diabetes UK.

GERA: The Genetic Epidemiology Research on Adult Health and Aging study was supported by grant RC2 AG036607 from the National Institutes of Health, grants from the Robert Wood Johnson Foundation, the Ellison Medical Foundation, the Wayne and Gladys Valley Foundation and Kaiser Permanente. The authors thank the Kaiser Permanente Medical Care Plan, Northern California Region (KPNC) members who have generously agreed to participate in the Kaiser Permanente Research Program on Genes, Environment and Health (RPGEH).

Supplementary Figures

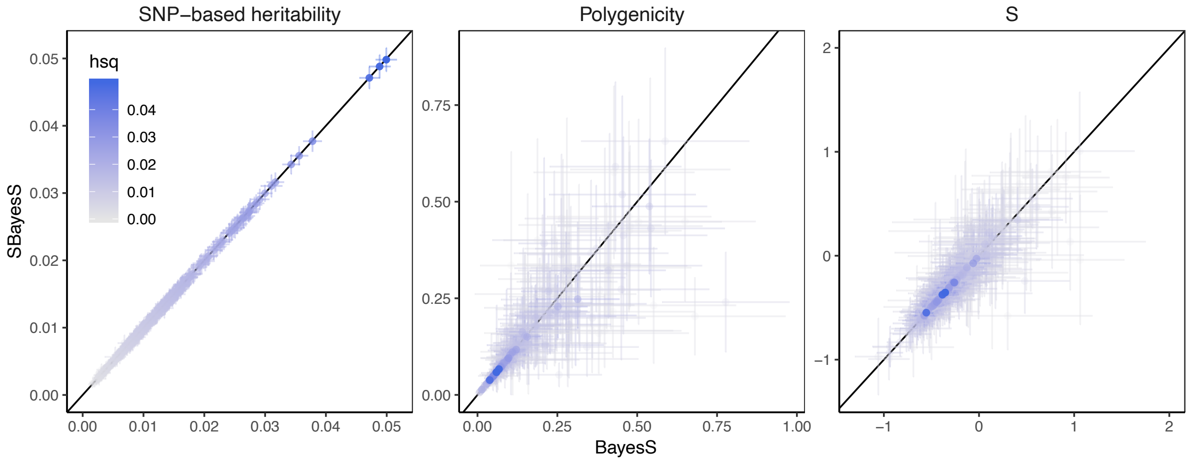

Supplementary Figure 1 Benchmarking SBayesS with BayesS using the same data in the chromosome-wide analysis across 18 UKB traits. The comparison was based on the unrelated individuals of European ancestry in the interim release of the UKB data (max n=120k) and ~500k array genotyped common SNPs (MAF>0.01). In the SBayesS analysis, the full LD matrix that included all pairwise LD was used for each chromosome. Data are presented as posterior means +/- posterior standard errors.

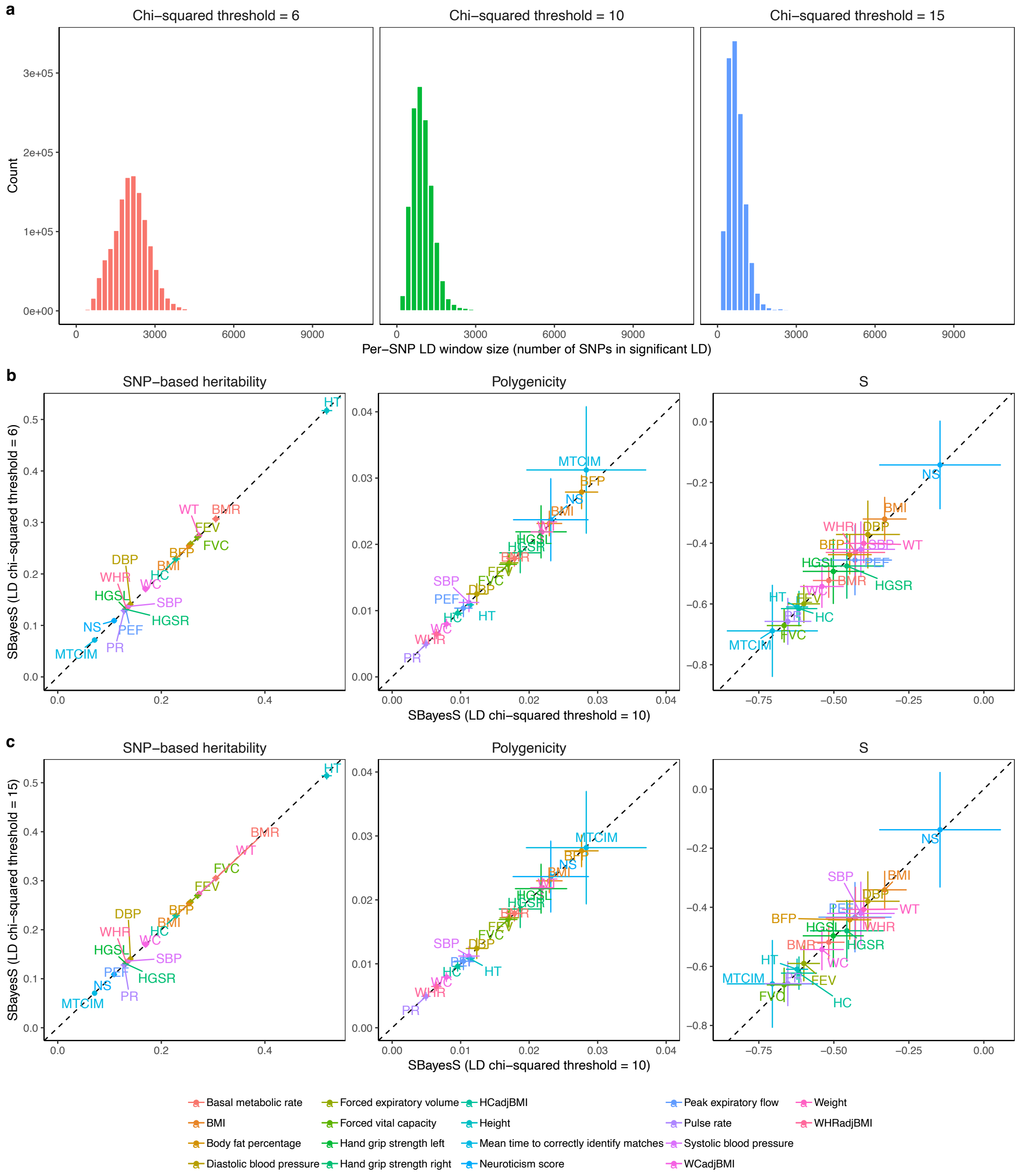

Supplementary Figure 2 Assessing the performance of SBayesS with different chi-squared thresholds used to make the sparse LD matrix. We computed summary statistics for 18 traits using the interim release of the UKB data (max n=120k) with ~1.1 million HapMap3 common SNPs. The sparse LD matrix was computed from a random sample of 50k unrelated individuals in the full UKB data with a chi-squared threshold of 6, 10 or 15 (corresponding to a r^2 threshold of 1, 2 or 3×10^{-4}, respectively). a) Distributions of the numbers of SNPs detected in LD with the target SNP given different chi-squared thresholds; b) Comparison between SBayesS results with chi-squared thresholds of 6 and 10; c) Comparison between SBayesS results with chi-squared thresholds of 10 and 15. Data are presented as posterior means +/- posterior standard errors.

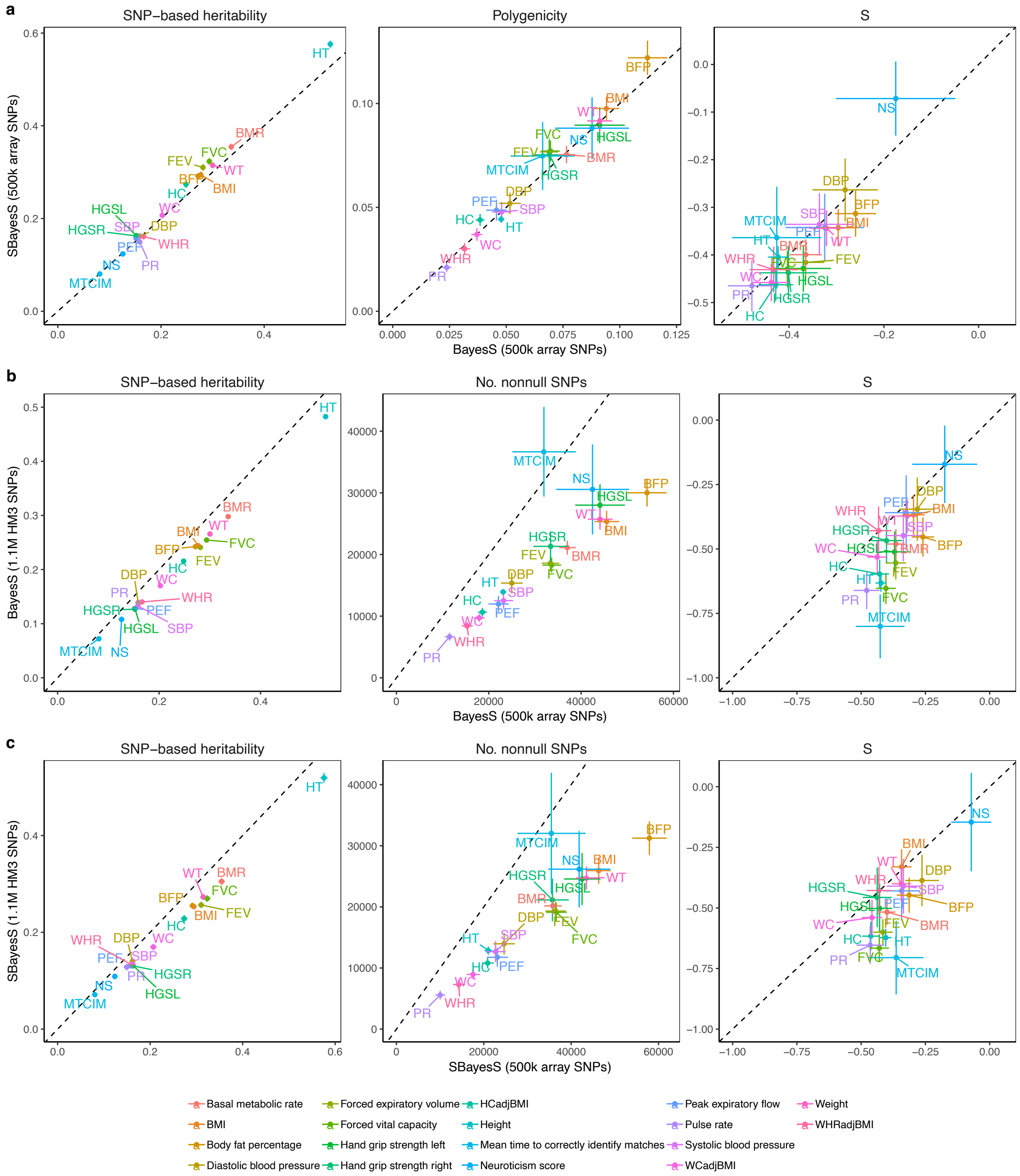

Supplementary Figure 3 Benchmarking SBayesS with BayesS given different SNP panels. We used the unrelated individuals of European ancestry in the interim release of the UKB data (max n=120k) and two SNP panels (~500k Affymetrix array SNPs and ~1.1 million HapMap3 SNPs) for the SBayesS analysis. The sparse LD matrix was computed from a random sample of 50k unrelated individuals from the full UKB cohort at a chi-squared threshold of 10. a) Comparison between SBayesS and BayesS using array SNPs; b) Comparison between BayesS results using HapMap3 and array SNPs; c) Comparison between SBayesS results using HapMap3 and array SNPs. For a fair comparison of π between panels, the number of SNPs with nonzero effects is shown in b) and c). Data are presented as posterior means +/- posterior standard errors.

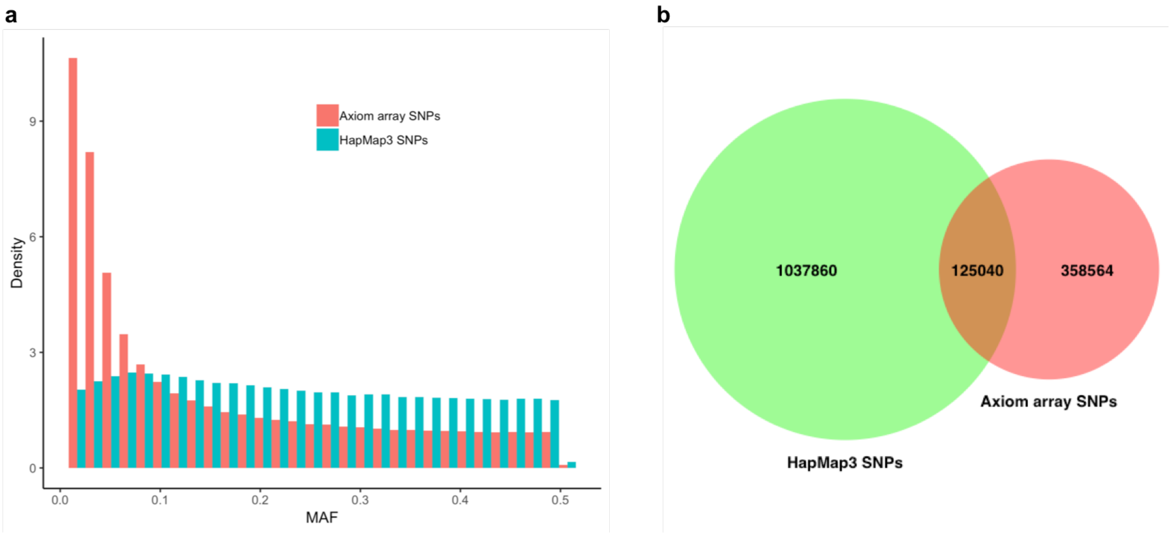

Supplementary Figure 4 Distributions of MAF of common SNPs (MAF>1%) in Affymetrix Axiom array and HapMap3 from the interim release of the UKB data. The array SNP panel was more enriched with low-frequency SNPs and had only a small overlap with the HapMap3 SNP panel. This may explain the differences in genetic architecture parameter estimates between array and HapMap3 SNPs (Supplementary Fig. 3). For example, the array SNPs might be more efficient to capture the low-frequency variance and therefore had slightly higher total SNP-based heritability. However, the majority of the common SNPs in HapMap3 Panel is believed to have better tagging to the causal variants. Thus, it is reasonable that the polygenicity estimates were lower than those with array SNPs because the model does not need multiple SNPs in low LD with the causal variants to jointly capture the causal effects. Similarly, a stronger estimate of S is expected because if the causal effects spread on multiple SNPs in LD, each SNP would have a relatively small effect size, which will dilute the signal for the relationship between effect size and MAF.

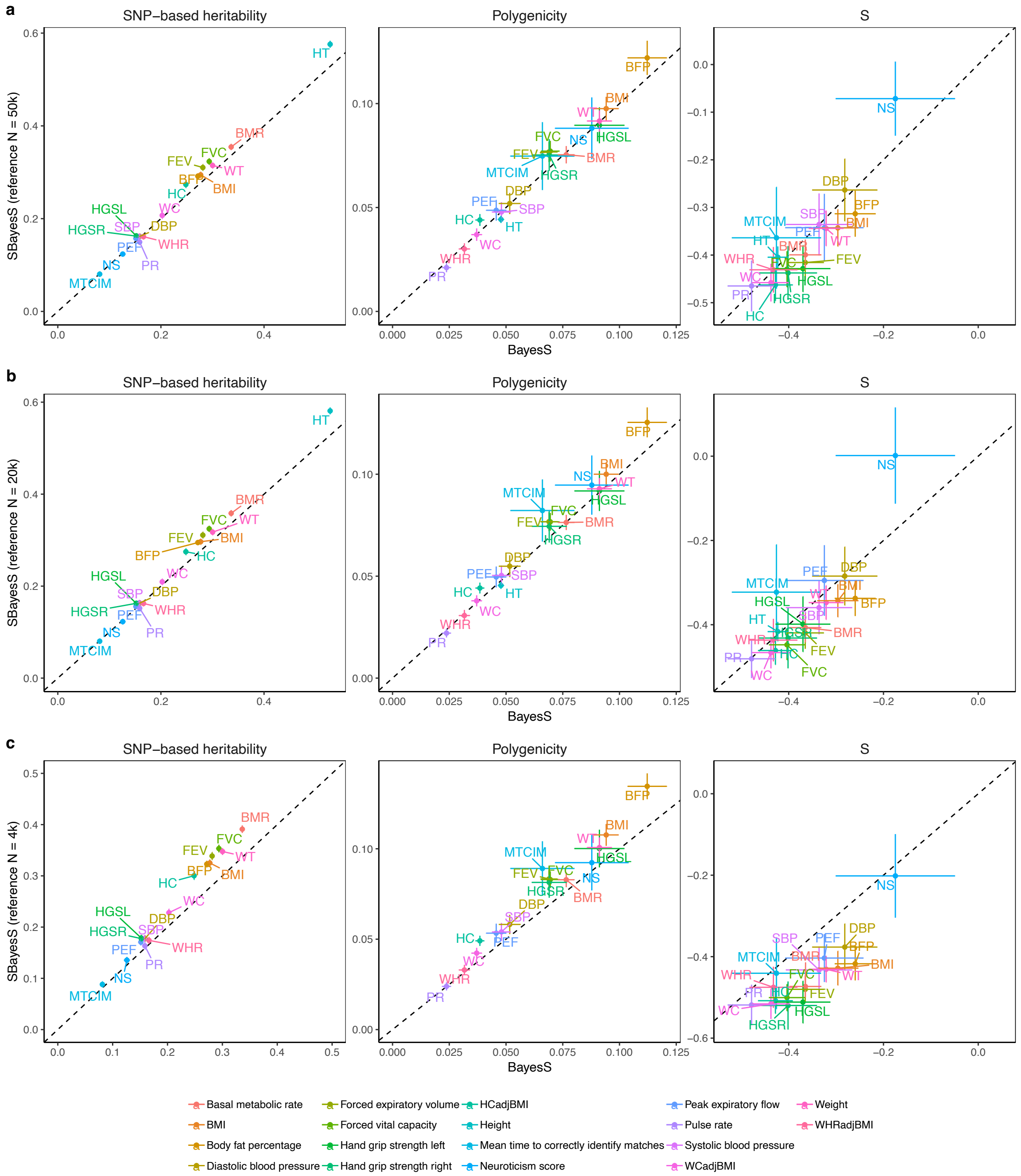

Supplementary Figure 5 Benchmarking SBayesS with BayesS given different reference sample sizes. We used the unrelated individuals of European ancestry in the interim release of the UKB data (max n=120k) and ~500k Affymetrix array common SNPs. The sparse LD matrix was computed from a random sample of a) 50k, b) 20k or c) 4k unrelated individuals from the full UKB data at a chi-square threshold of 10. The inflation in parameter estimation increased when the reference sample size was too small. When the reference sample size was 4k, SBayesS analysis for height did not converge. Data are presented as posterior means +/- posterior standard errors.

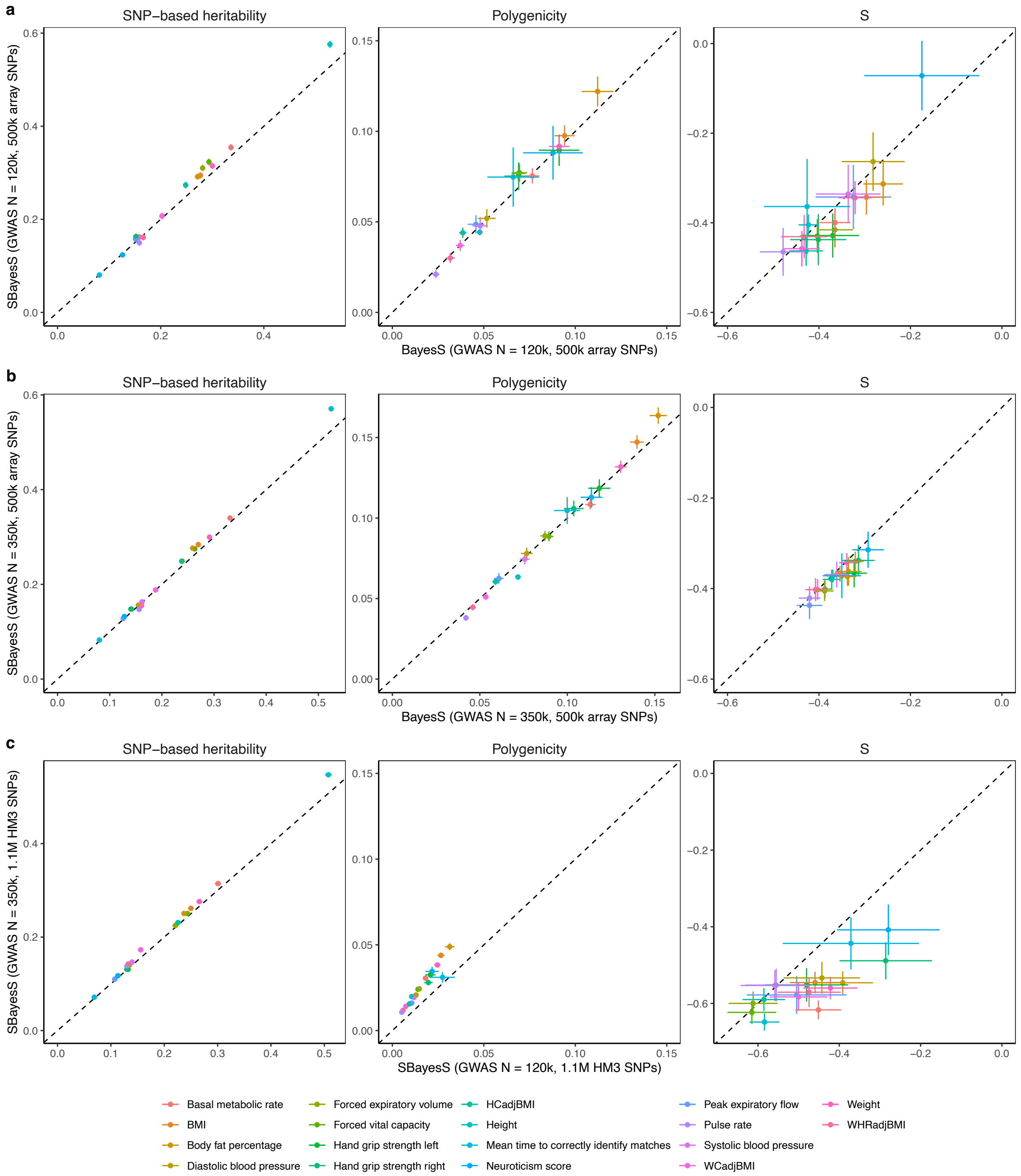

Supplementary Figure 6 Benchmarking SBayesS with BayesS given different GWAS sample sizes. a) and b) Comparison between SBayesS and BayesS using the unrelated individuals of European ancestry in the interim (max n=120k) and full (max n=350k) releases of the UKB data and ~500k Affymetrix array SNPs. c) Comparison between SBayesS results given GWAS sample size of 350k and 120k using ~1.1 million HapMap3 SNPs. The sparse LD matrix used in SBayesS was computed from a random sample of 50k unrelated individuals from the full UKB data at a chi-squared threshold of 10. Data are presented as posterior means +/- posterior standard errors.

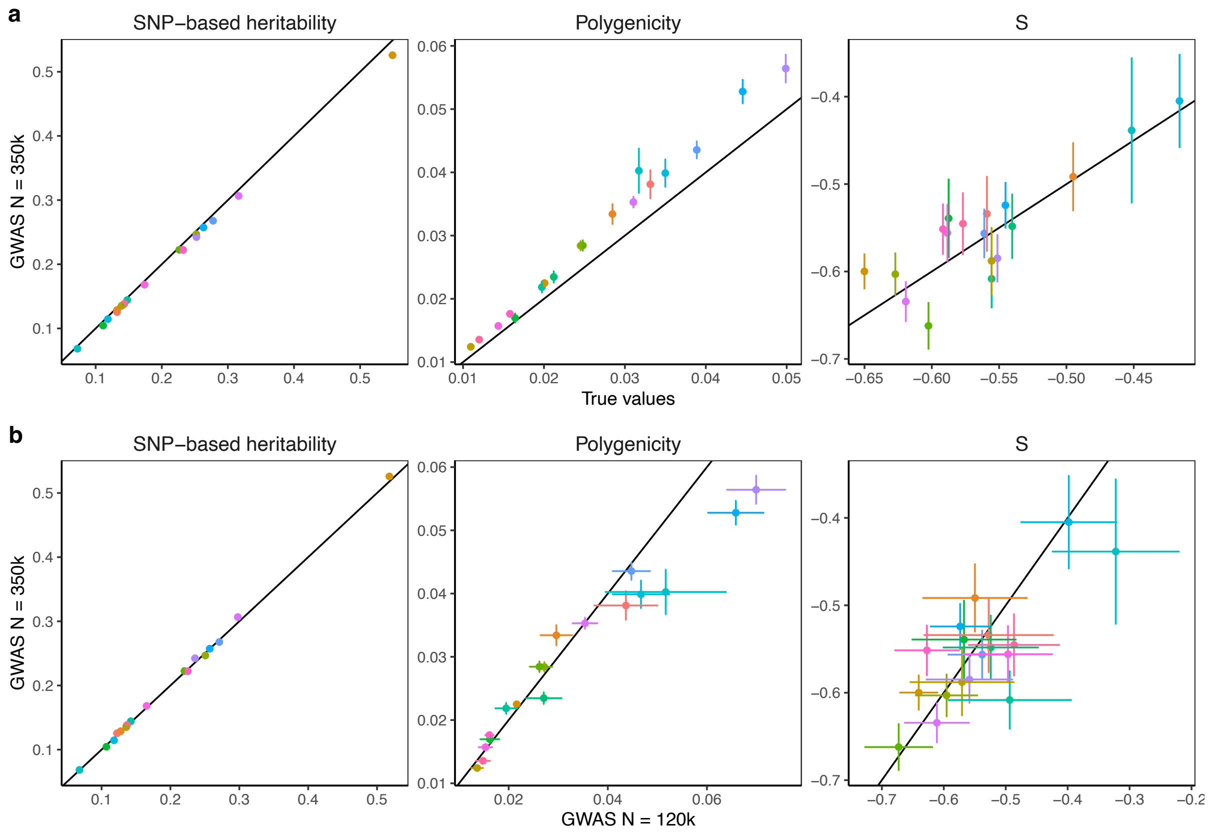

Supplementary Figure 7 SBayesS results of the simulation study based on the parameter estimates from the real trait analysis. a) Comparison of parameter estimates based on the SNP markers (causal variants excluded) and the true parameter values at the causal variants in the simulation. b) Comparison of parameter estimates with GWAS sample sizes of 350K and 120K. Data are presented as posterior means +/- posterior standard errors.

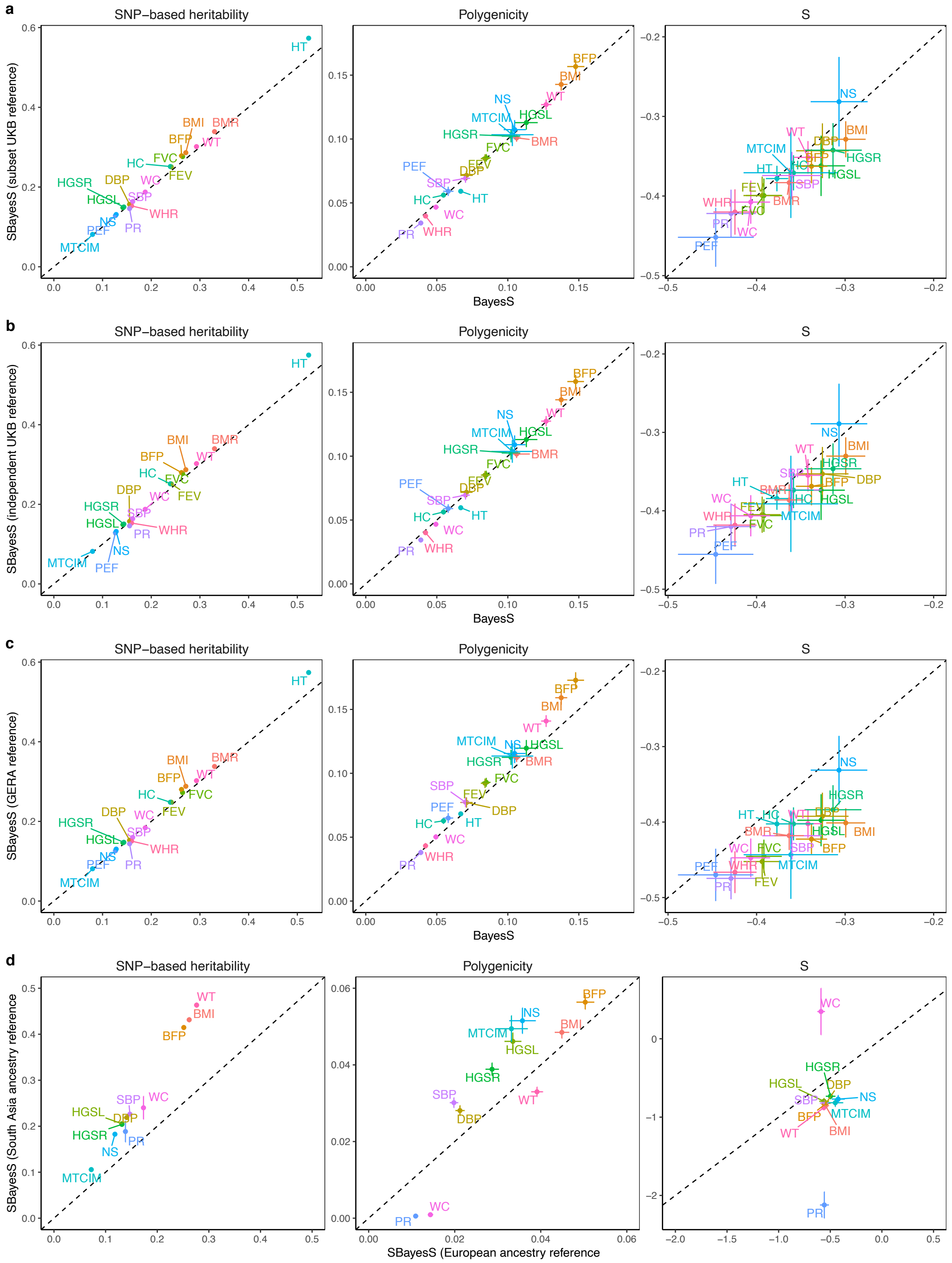

Supplementary Figure 8 Benchmarking SBayesS with BayesS given different LD references. We used phenotypes from a random sample of 300k unrelated individuals of European ancestry from the full UKB data and ~500k Affymetrix array SNPs. The sparse LD matrix with a chi-squared threshold of 10 used in SBayesS was computed from a) a subset sample of 50k UKB individuals, b) an independent sample of 50k UKB individuals, or c) 50k unrelated individuals

from the GERA dataset. d) When GWAS data were from the UKB European population, using a sample (n = 9948) of South Asia ancestry as LD reference in SBayesS (1.1 million HapMap3 SNPs) resulted in a severe bias to the genetic architecture parameter estimates and a failure in convergence for 11 out of the 18 traits (61%) with a chromosome-wide full LD matrix (all traits were failed in convergence when using a sparse LD matrix with the default sparsity, i.e., chi-squared statistic threshold of 10). Data are presented as posterior means +/- posterior standard errors.

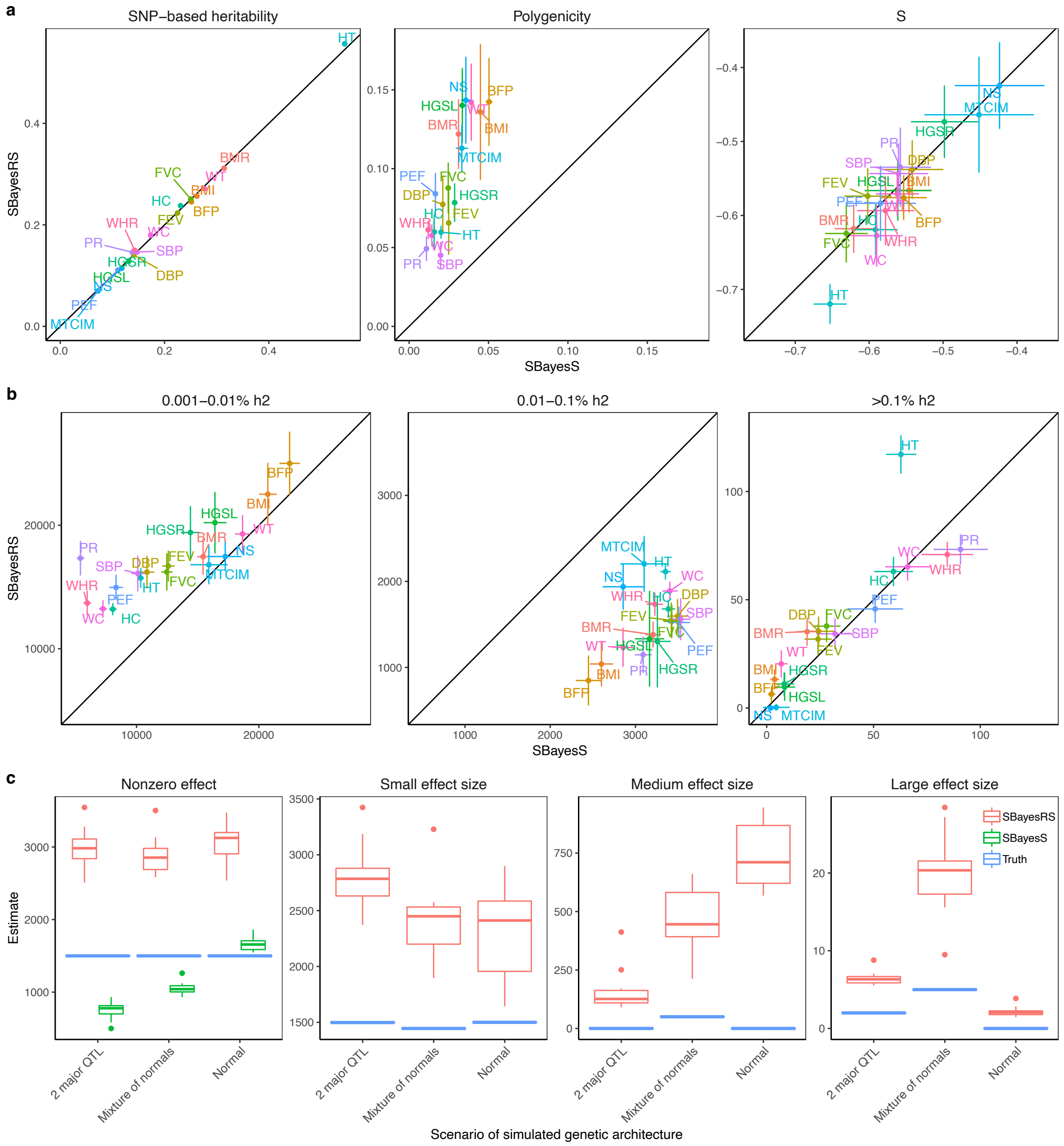

Supplementary Figure 9 Comparison between SBayesS and SBayesRS for the analyses of 18 quantitative traits in the UKB. a) Estimates of genetic architecture parameters, i.e. *S*, SNP-based heritability and polygenicity. b) Estimated number of SNPs that explain 0.001-0.01%, 0.01-0.1% and >0.1% of the total SNP-based heritability in SBayesRS and SBayesS. Data are presented as posterior means +/- posterior standard errors. c) Estimated number of SNPs with nonzero effects as well as that in the small, medium and large mixture components in the simulation on chromosomes 21 and 22. The band inside the box is the median, the bottom and top of the box are the first and third quartiles, respectively (Q1 and Q3), and the lower and upper whiskers are Q1 – 1.5 IQR and Q3 + 1.5 IQR, respectively, where IQR = Q3 – Q1.

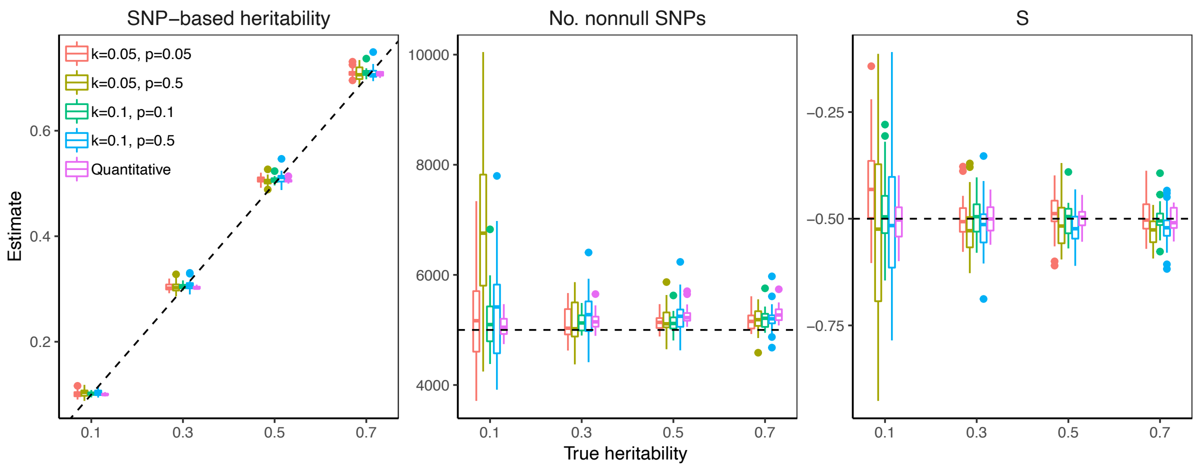

Supplementary Figure 10 Estimation of the three genetic architecture parameters using SBayesS with simulated data for quantitative traits or case-control studies with different population (k) and sample (p) prevalence. The band inside the box is the median, the bottom and top of the box are the first and third quartiles, respectively (Q1 and Q3), and the lower and upper whiskers are Q1 – 1.5 IQR and Q3 + 1.5 IQR, respectively, where IQR = Q3 – Q1. We used the full UKB data (n=350k) for simulations, where 5k SNPs were randomly chosen from ~1.1 million HapMap3 common SNPs as causal variants with true S=-0.5, and the trait heritability was set to be 0.1, 0.3, 0.5 or 0.7 (at the liability scale for the binary trait). The sparse LD matrix used in SBayesS was computed from a subset sample of 50k UKB individuals with a chi-squared threshold of 10. The simulation was repeated 30 times for each scenario. When k=0.05, p=0.5 and true heritability=0.1, the polygenicity estimate tend to bias upward with large estimation variation, which is likely due to insufficient power to distinguish the model that fits only causal variants from that fits multiple SNPs in LD with the causal variants.

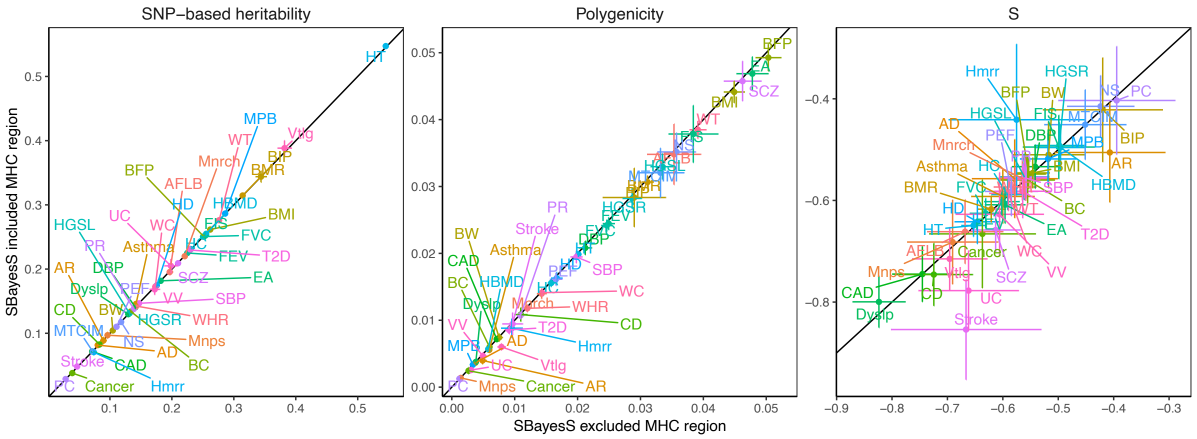

Supplementary Figure 11 Comparison between SBayesS results including versus excluding SNPs in the MHC region across 44 complex traits in this study (26 UKB complex traits, 9 UKB diseases and 9 diseases from public GWAS). Data are presented as posterior means +/- posterior standard errors. Colours indicate different traits and diseases.

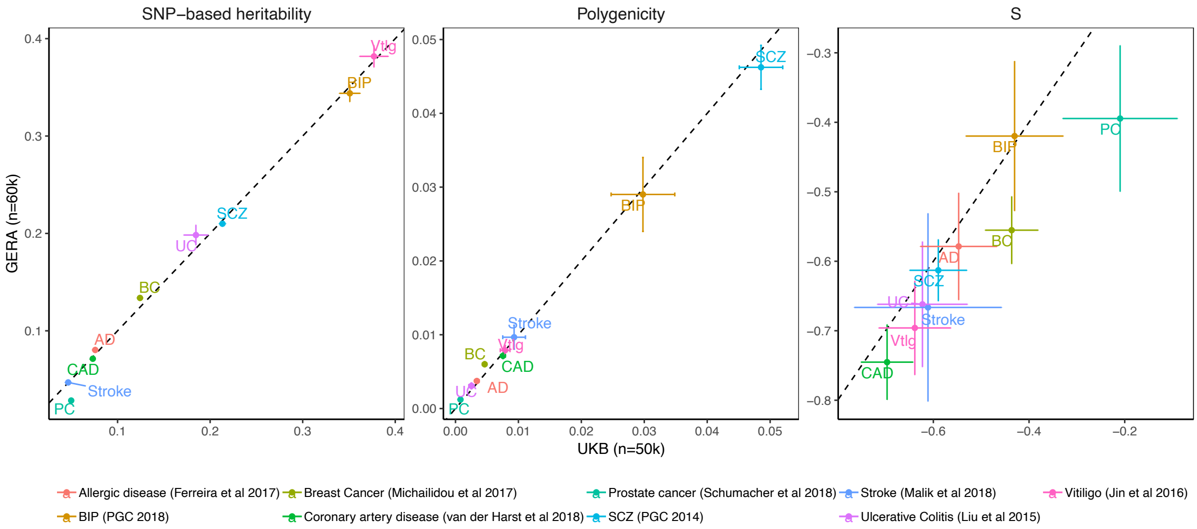

Supplementary Figure 12 Comparison between SBayesS results based on LD from GERA and UKB (a random subsample of 50k unrelated individuals) using published GWAS summary data for 9 diseases. Data are presented as posterior means +/- posterior standard errors. Colours with acronyms indicate different traits, whose full names are shown at the bottom of the figure.

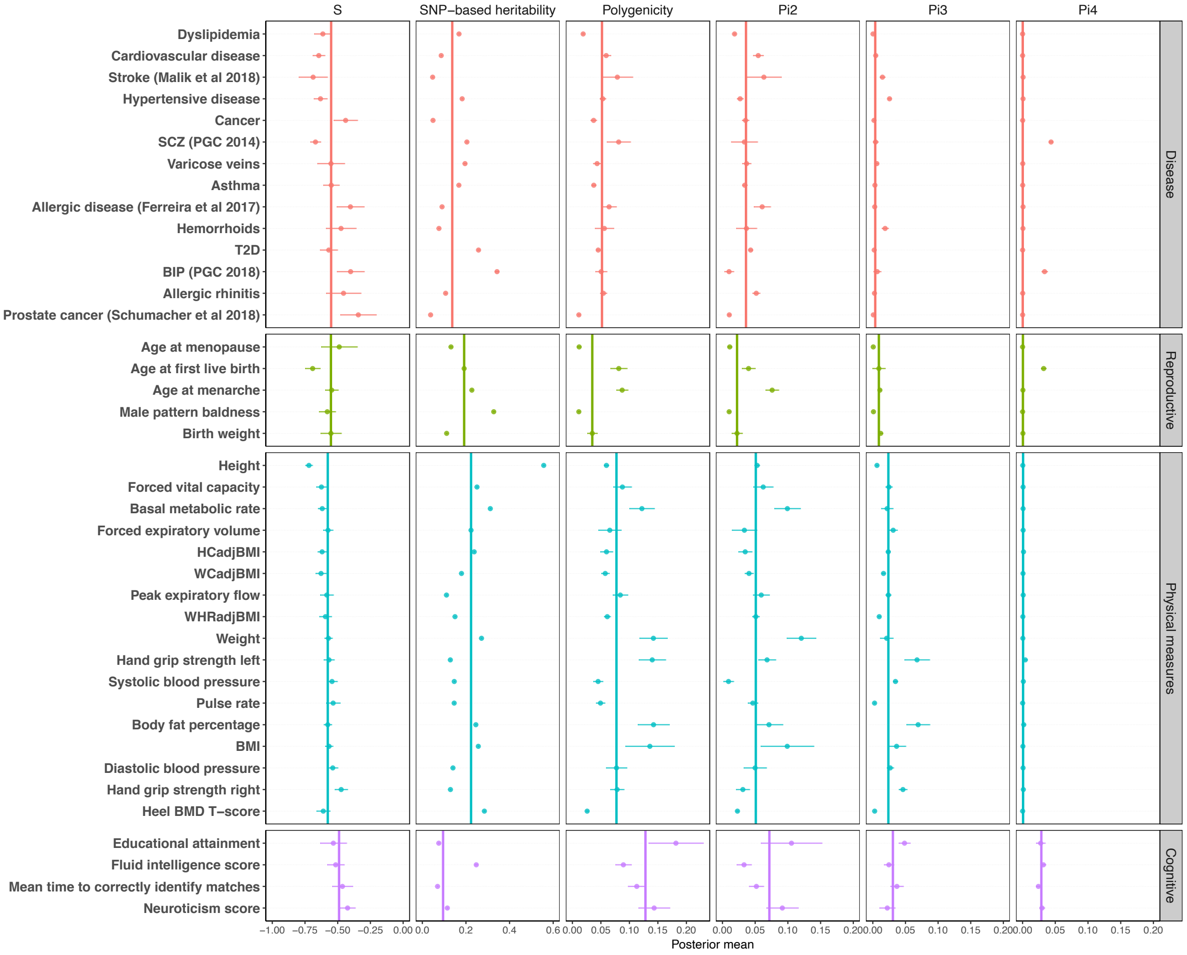

Supplementary Figure 13 Estimation of the three genetic architecture parameters for 35 complex traits (including diseases) in UKB (max n=350k) and 5 common diseases from published GWAS (labelled with publications) by SBayesRS. Shown are the posterior means (dots) and standard errors (horizontal bars) of the parameters for each trait. The colour indicates the category that the trait belongs to. The vertical bar shows the median of the estimates across traits in each category. Pi2, Pi3 and Pi4 show the proportions of SNPs in the small, medium and large effect size components of the mixture distribution in SBayesRS. Traits are in the same order as in Figure 2 for comparison. Coronary artery disease (van der Harst et al 2018), Vitiligo (Jin et al 2016) and Ulcerative Colitis (Liu et al 2015) and Breast cancer (Michallidou et al 2017) did not have converged results.

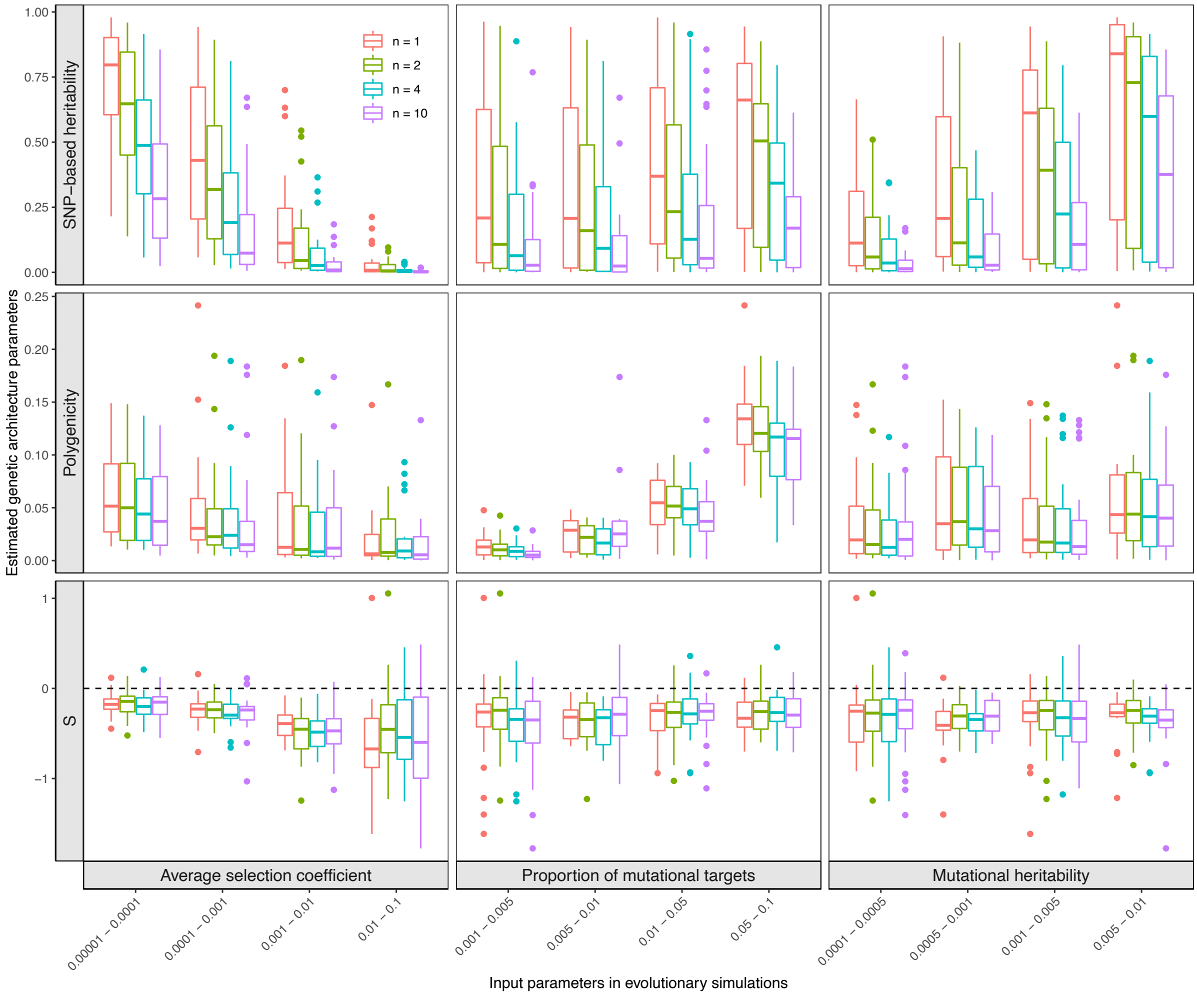

Supplementary Figure 14 Variational patterns of the estimated genetic architecture parameters under different scenarios of evolutionary simulations, when selection coefficients followed a mixture distribution. The Simons et al. pleiotropic model was used to generate genetic effects. The x-axis shows the values of three input parameters in evolutionary simulations. The y-axis shows the distribution of the genetic architecture parameter estimated by SBayesS. Colour shows the results under the model of Simons et al with n_t = 1, 2, 4 or 10. It can be seen that different n_t only affected the SNP-based heritability. The band inside the box is the median, the bottom and top of the box are the first and third quartiles, respectively (Q1 and Q3), and the lower and upper whiskers are Q1 – 1.5 IQR and Q3 + 1.5 IQR, respectively, where IQR = Q3 – Q1.

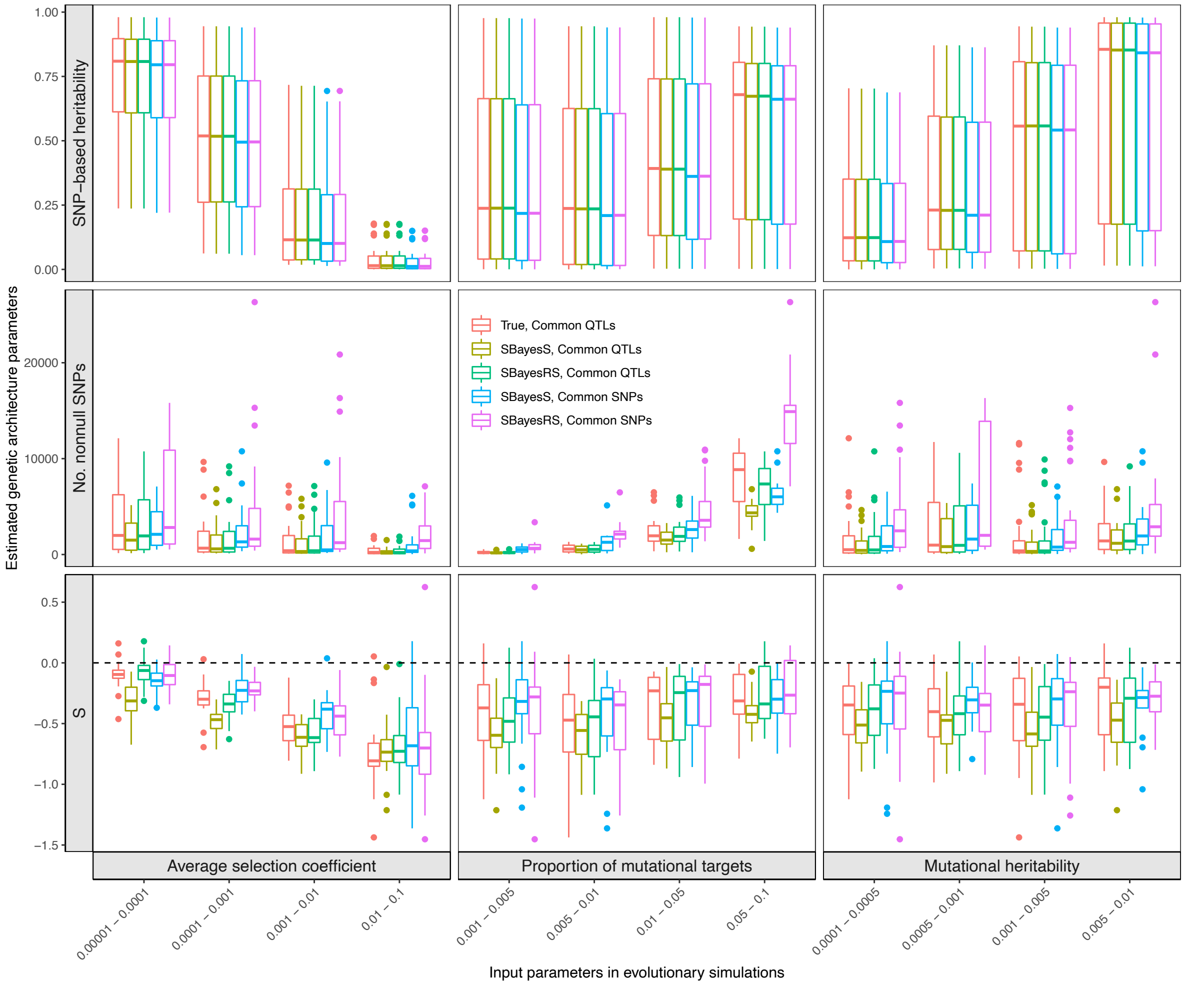

Supplementary Figure 15 Variational patterns of the estimated genetic architecture parameters under different scenarios of evolutionary simulations, when selection coefficients followed a mixture distribution. The Eyre-Walker model was used to generate genetic effects ($\tau = 0.5$ and $\sigma^2 = 0.1$). The x-axis shows the values of three input parameters in evolutionary simulations. The y-axis shows the distribution of the genetic architecture parameter estimates, where the polygenicity parameter is represented by the number of nonnull SNPs for better benchmarking. "True, Common QTLs": parameters computed directly from the simulated genetic effects of all common causal variants; "SBayesS, Common QTLs" (or "SBayesRS, Common QTLs"): SBayesS (or SBayesRS) estimates using the genotype data of the common causal variants and the phenotypes; "SBayesS, Common SNPs" (or "SBayesRS, Common SNPs"): SBayesS (or SBayesRS) estimates using the genotype data of 36k common SNPs and the simulated genetic values. The band inside the box is the median, the bottom and top of the box are the first and third quartiles, respectively (Q1 and Q3), and the lower and upper whiskers are Q1 – 1.5 IQR and Q3 + 1.5 IQR, respectively, where IQR = Q3 – Q1.

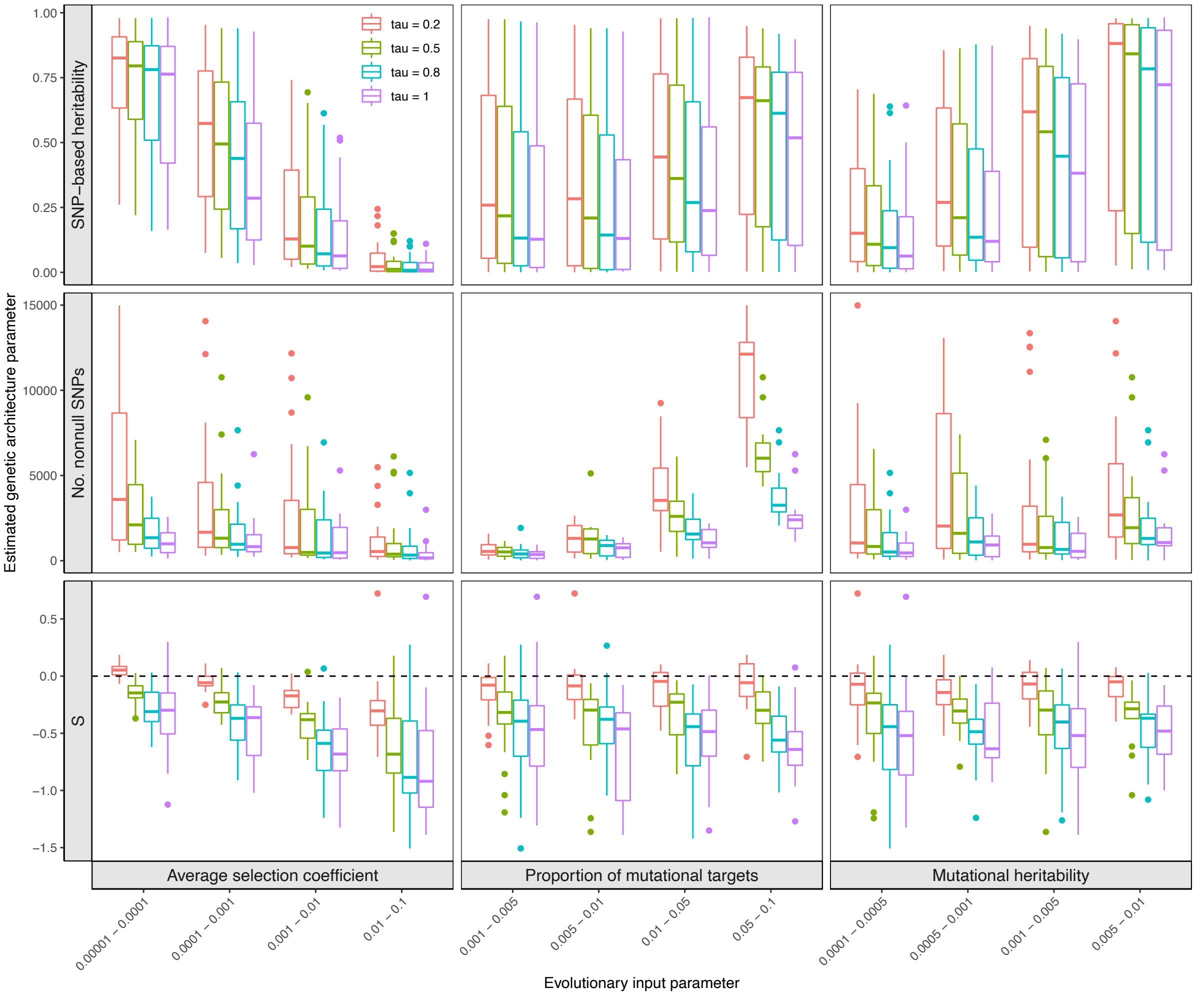

Supplementary Figure 16 Variational patterns of the estimated genetic architecture parameters under different scenarios of evolutionary simulations, when selection coefficients followed a mixture distribution. The Eyre-Walker model was used to generate genetic effects. The x-axis shows the values of three input parameters in evolutionary simulations. The y-axis shows the distribution of the genetic architecture parameters estimated by SBayesS. Colour shows the results under the Eyre-Walker model with $\tau = 0.2, 0.5, 0.8$ or 1. It can be seen that the genetic architecture parameter estimates were subject to τ. Each box plot shows the results of 25 independent simulation replicates. The band inside the box is the median, the bottom and top of the box are the first and third quartiles, respectively (Q1 and Q3), and the lower and upper whiskers are Q1 – 1.5 IQR and Q3 + 1.5 IQR, respectively, where IQR = Q3 – Q1.

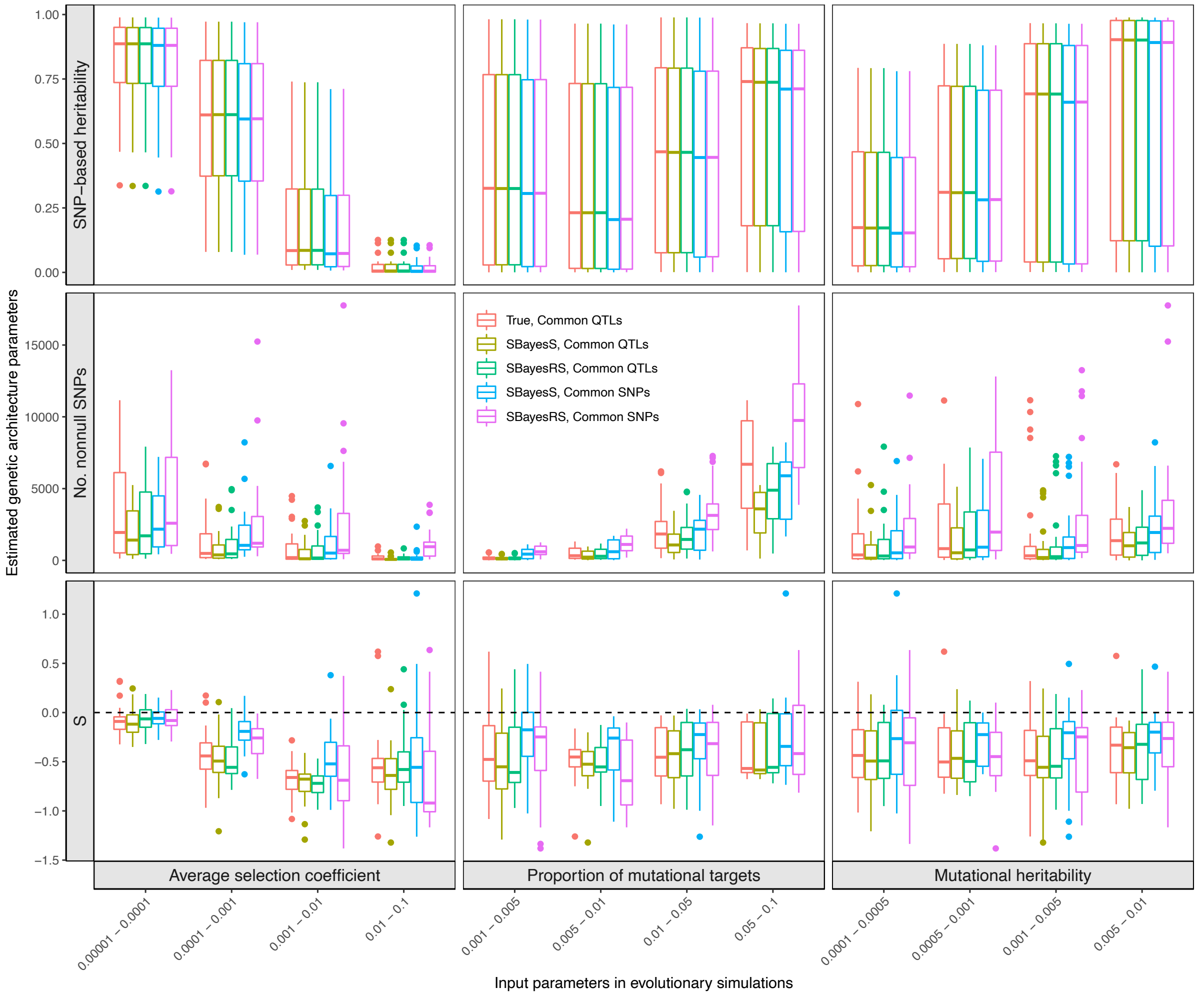

Supplementary Figure 17 Variational patterns of the estimated genetic architecture parameters under different evolutionary simulation scenarios, when selection coefficients followed a normal distribution. The Simons et al. pleiotropic model with n_t =1 was used to generate genetic effects (Methods). The x-axis shows the values of three input parameters in the evolutionary simulations. The y-axis shows the distribution of the genetic architecture parameter estimates, where the polygenicity parameter is represented by the number of nonnull SNPs for better benchmarking. “True, Common QTLs”: parameters computed directly from the simulated genetic effects of all common causal variants; “SBayesS, Common QTLs” (or “SBayesRS, Common QTLs”): SBayesS (or SBayesRS) estimates using the genotype data of the common causal variants and the phenotypes; “SBayesS, Common SNPs” (or “SBayesRS, Common SNPs”): SBayesS (or SBayesRS) estimates using the genotype data of 36k common SNPs and the simulated genetic values. Each box plot shows the results of 25 independent simulation replicates. The band inside the box is the median, the bottom and top of the box are the first and third quartiles, respectively (Q1 and Q3), and the lower and upper whiskers are Q1 – 1.5 IQR and Q3 + 1.5 IQR, respectively, where IQR = Q3 – Q1.

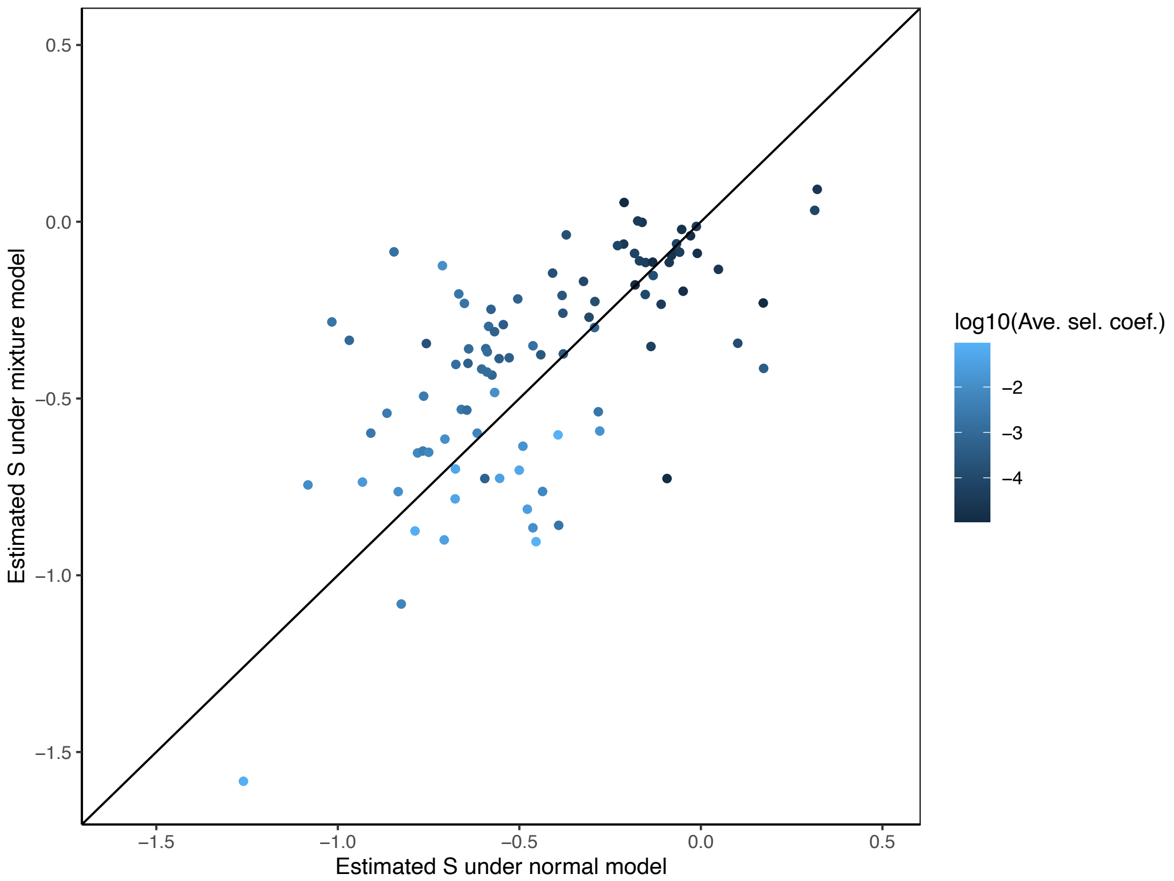

Supplementary Figure 18 Comparison of OLS estimates of S based on the true causal effects under the mixture distribution and the normal distribution for simulating the selection coefficients.

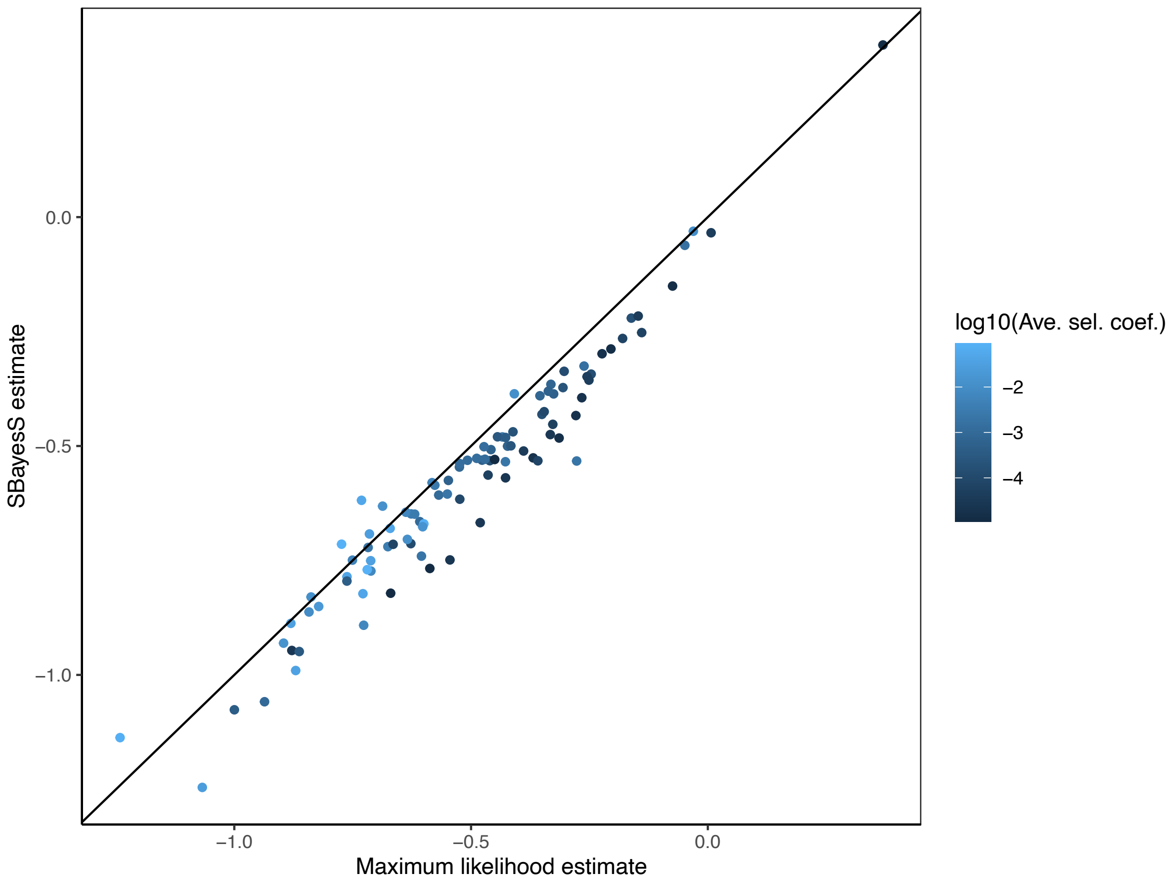

Supplementary Figure 19 SBayesS estimates of S using causal variant genotypes are consistent with the MLE estimates based on the causal variant effects.

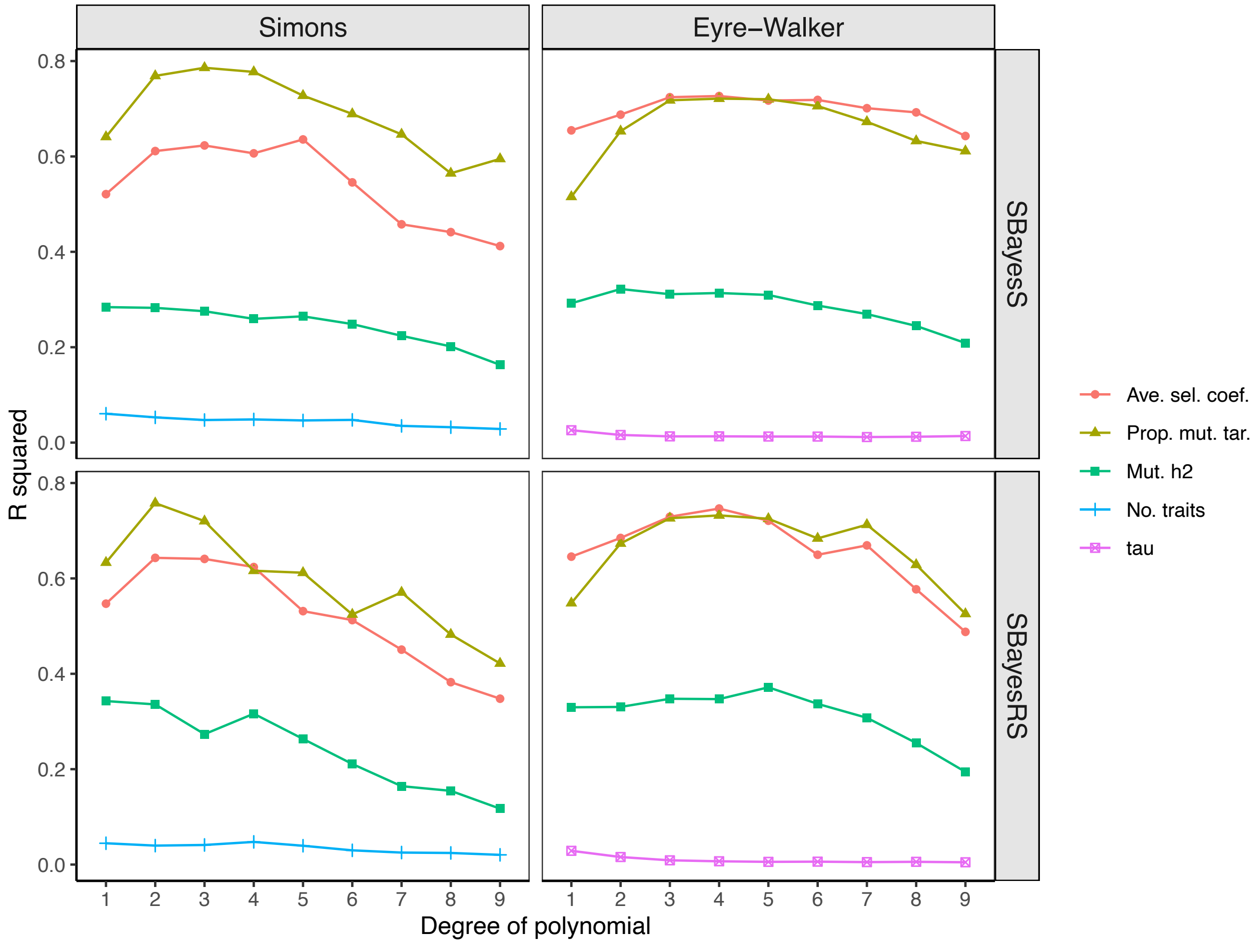

Supplementary Figure 20 Prediction R2 in cross-validation for predicting the evolutionary parameters using a polynomial regression with the simulated data under the Simons et al or Eyre-Walker model. The predictors in the polynomial regression were the estimated genetic architecture parameters, which were $\hat{h}^2_{SNP}$, $\hat{\pi}$ and $\hat{S}$ in SBayesS or $\hat{h}^2_{SNP}$, $\hat{\pi}_1$, $\hat{\pi}_2$, $\hat{\pi}_3$, $\hat{\pi}_4$ and $\hat{S}$ in SBayesRS. The response variables were $\bar{s}$ (average selection coefficient), π_m (proportion of mutational targets) or h^2_m (mutational heritability) at log10 scale, or exclusive parameters specific to each model, namely, the number of traits (n_t) in the model of Simons et al. and τ and σ^2 in the Eyre-Walker's model. The prediction R2 was computed from cross-validation with 80% of simulation data used as training and the rest as validation. It can be seen that there was reasonably high power to predict $\bar{s}$, π_m and h^2_m but no power to predict n_t and τ.

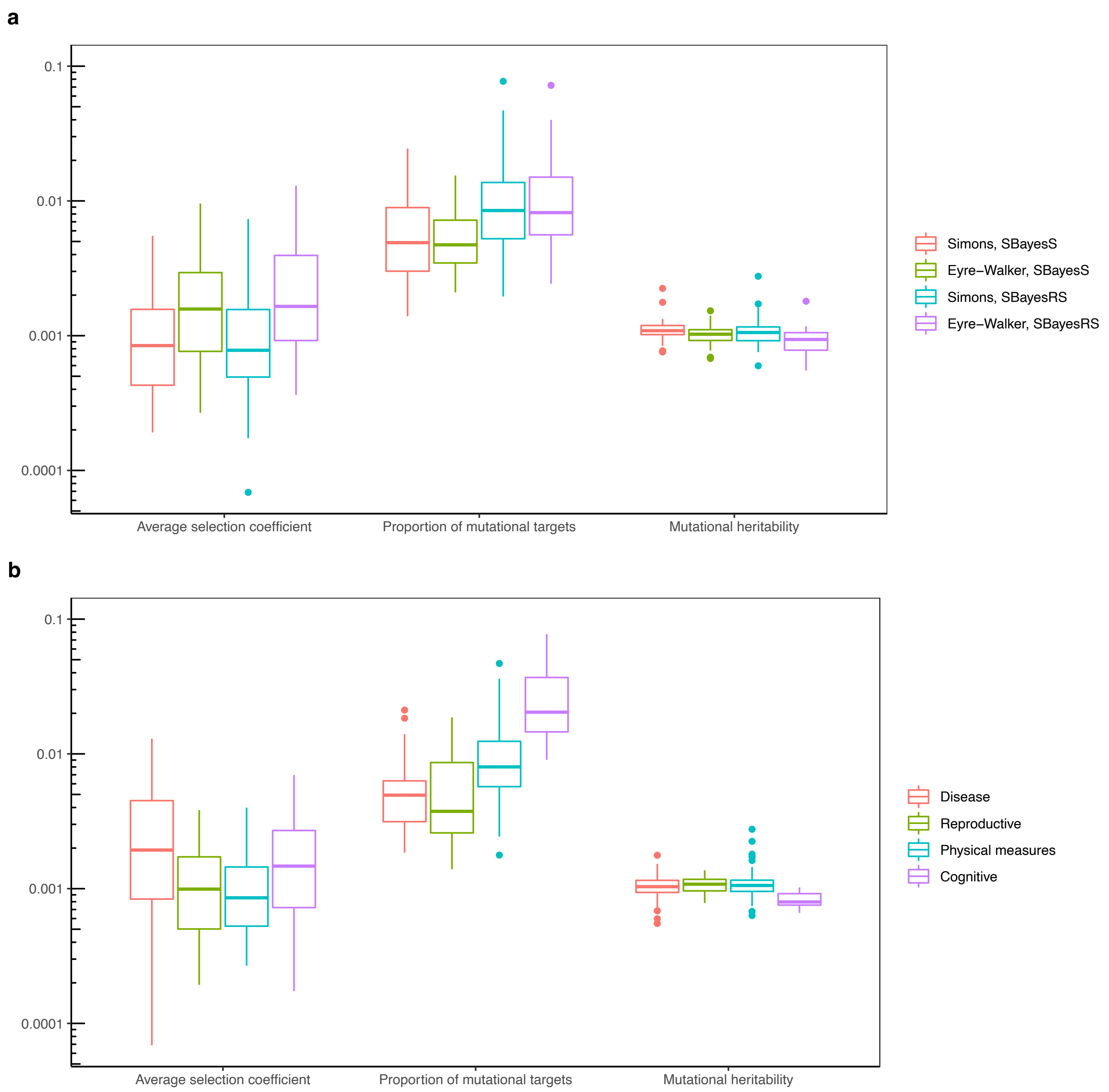

Supplementary Figure 21 Prediction of the evolutionary parameters for 44 complex traits and diseases based on a negative selection model where selection coefficients followed a normal distribution. a) Distribution of the predicted evolutionary parameters under different scenarios: methods used for estimating the genetic architecture parameters (SBayesS and SBayesRS) and pleiotropic effect models used for simulations (the Simons et al. and Eyre-Walker models). b) Distribution of predicted evolutionary parameters for four trait categories. Each box plot shows the results for a number of traits in a category, with each trait having four results from analyses using different estimation methods and simulation models. The band inside the box is the median, the bottom and top of the box are the first and third quartiles, respectively (Q1 and Q3), and the lower and upper whiskers are Q1 – 1.5 IQR and Q3 + 1.5 IQR, respectively, where IQR = Q3 – Q1.

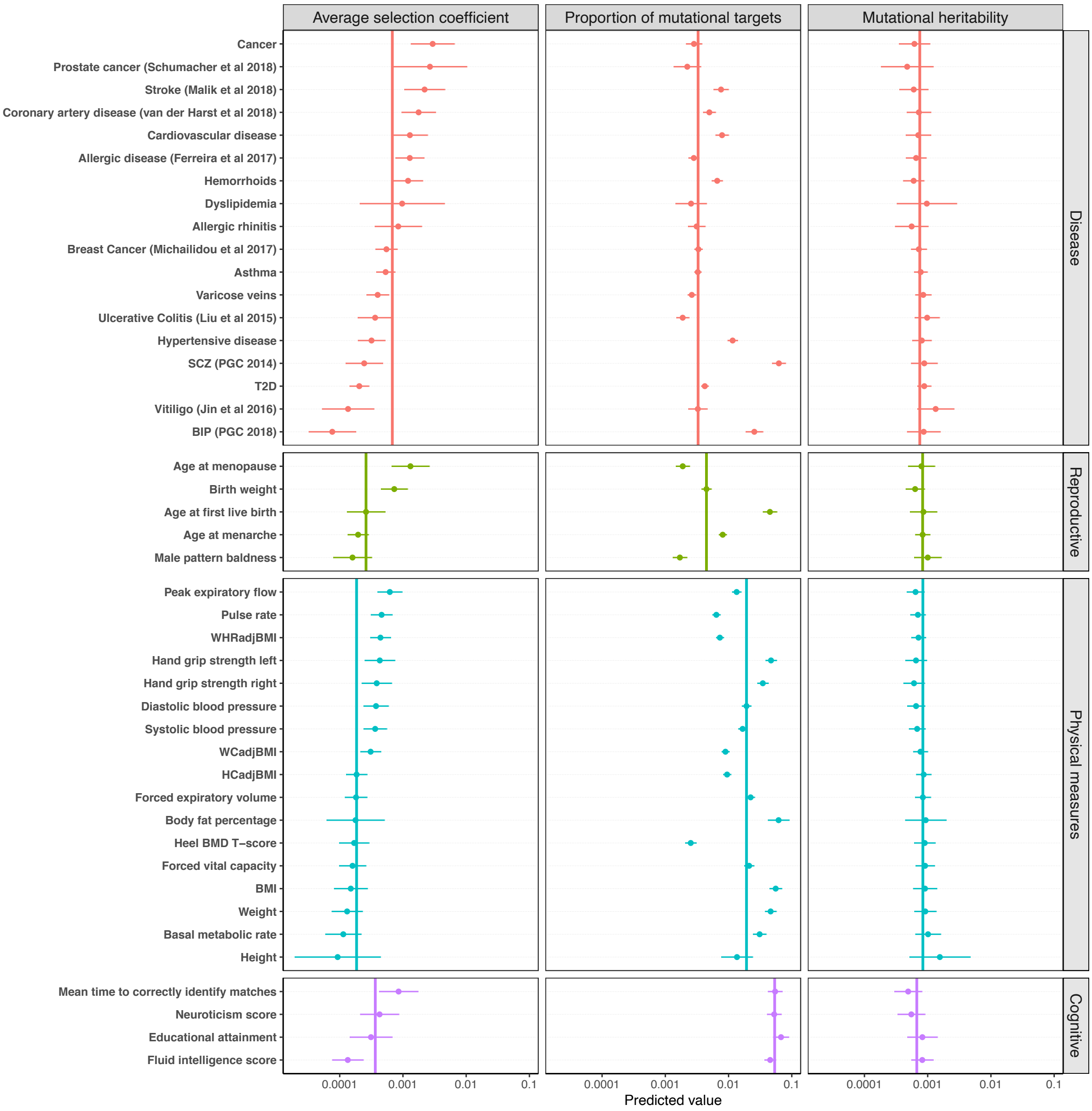

Supplementary Figure 22 Predicted values of the evolutionary parameters based on the Simons et al. model for simulation and SBayesS estimates of genetic architecture parameters. The horizontal bar shows the standard error of the predicted value. The vertical bar shows the median value in each trait category.

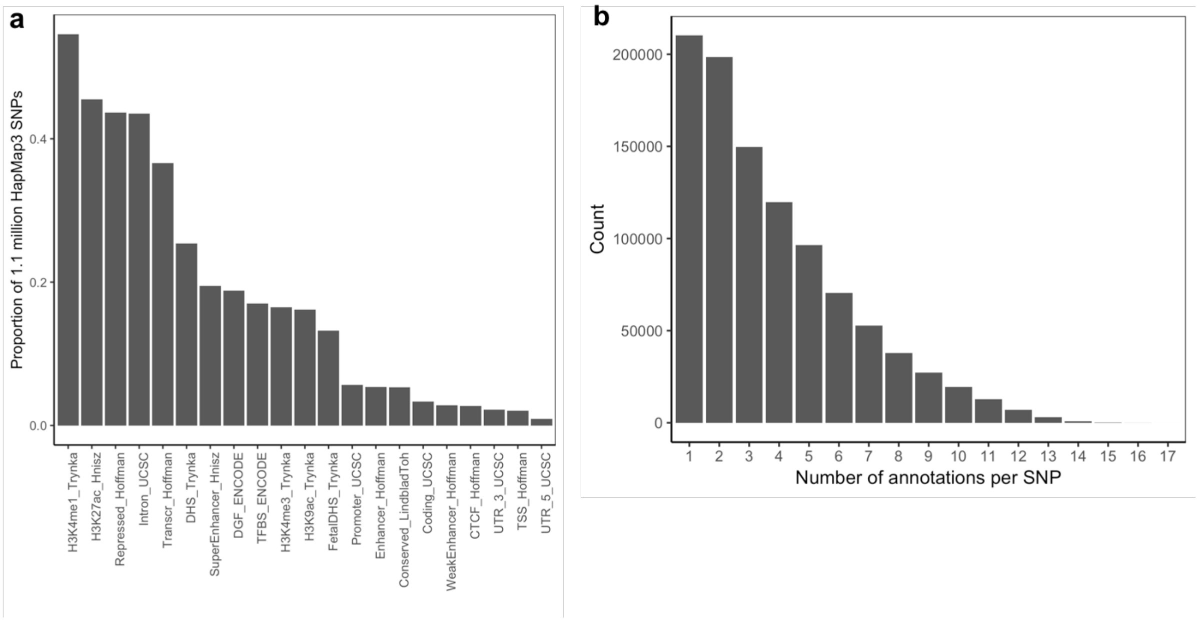

Supplementary Figure 23 Summary of the 21 functional annotation categories from the LDSC baseline model. a) The proportion of 1.1 million HapMap3 common SNPs used in the analysis in each functional category. b) The distribution of the number of annotations for each SNP.

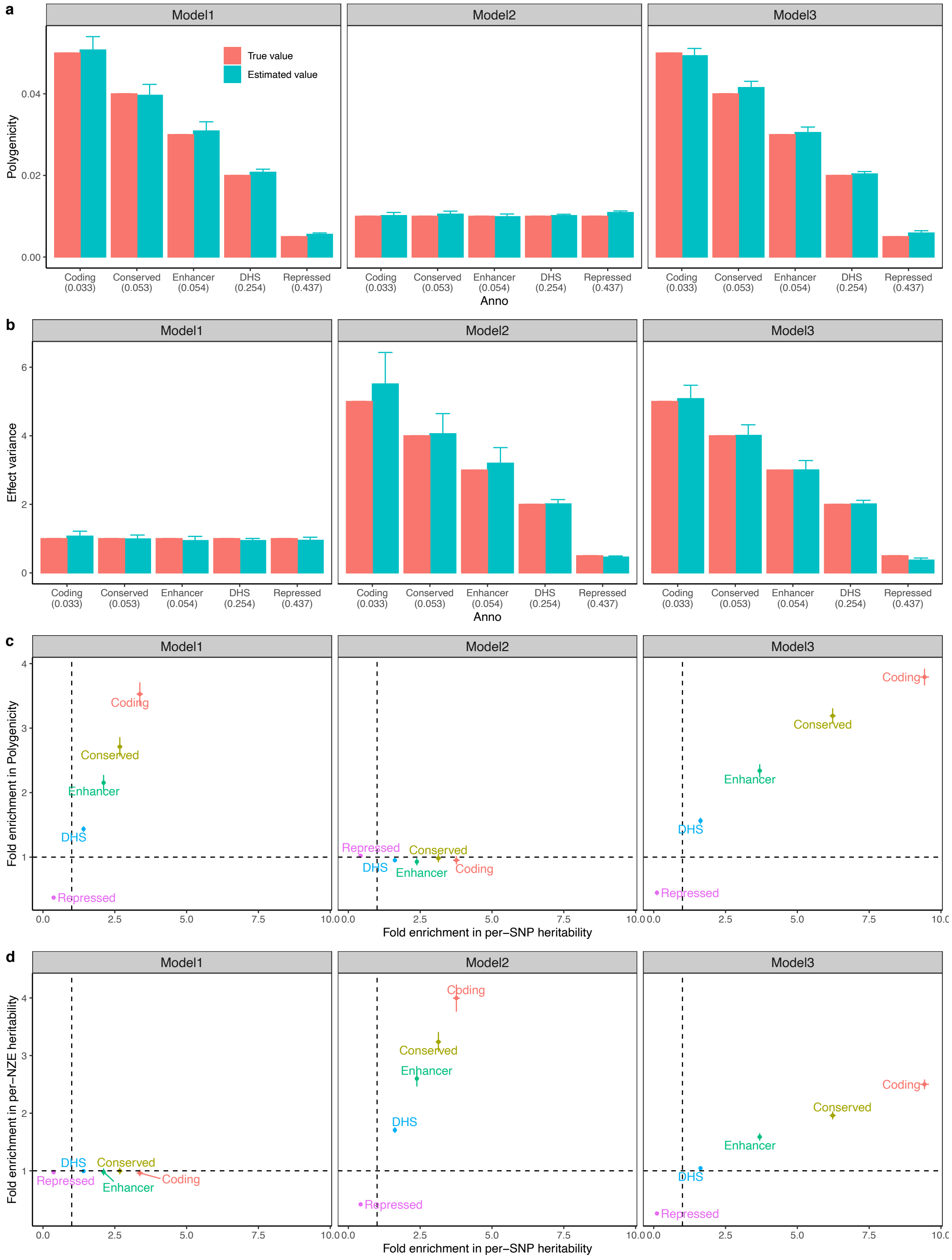

Supplementary Figure 24 Estimation of parameters and their enrichment under different simulated models of genetic architecture across functional annotation categories. We used the UKB data with ~1.1 million HapMap3 common SNPs for simulation and considered three models to simulate the distribution of causal effects. In model 1, the proportion of causal variants was set to 0.5, 2, 3, 4 and 5% for the repressed, DHS, enhancer, conserved and coding regions, respectively, and 1% for the rest of the genome, with the variance of causal effects = 1.

In model 2, the variance of causal effects was set to 0.5, 2, 3, 4 and 5 for the five functional annotation categories and 1 for the rest of the genome, with the proportion of causal variants = 1%. In model 3, we varied both the proportion of causal variants and the variance of causal effects across categories as in model 1 and 2. The trait heritability was set to 0.5. The annotation data were selected from the LDSC baseline model, which had extensive annotation overlaps. When a causal variant had more than one annotation, we randomly assigned one of the overlapping annotations to the causal variant in the simulation. In the analysis, we used the true annotations, where all annotations were mutually disjoint in each replicate, and then run the 2-component SBayesS-strat model. Results are a) Estimated polygenicity parameter under different models; b) Estimated effect variance under in different models; c) Fold enrichment in polygenicity compared with that in per-SNP heritability under different models; and d) Fold enrichment in per-NZE heritability compared with that in per-SNP heritability under different models. In a) and b), the true value is shown in red bar and the number in the a-axis label shows the proportion of SNPs in the corresponding annotation. Data are presented as mean values +/- standard errors of the means across 30 independent simulation replicates.

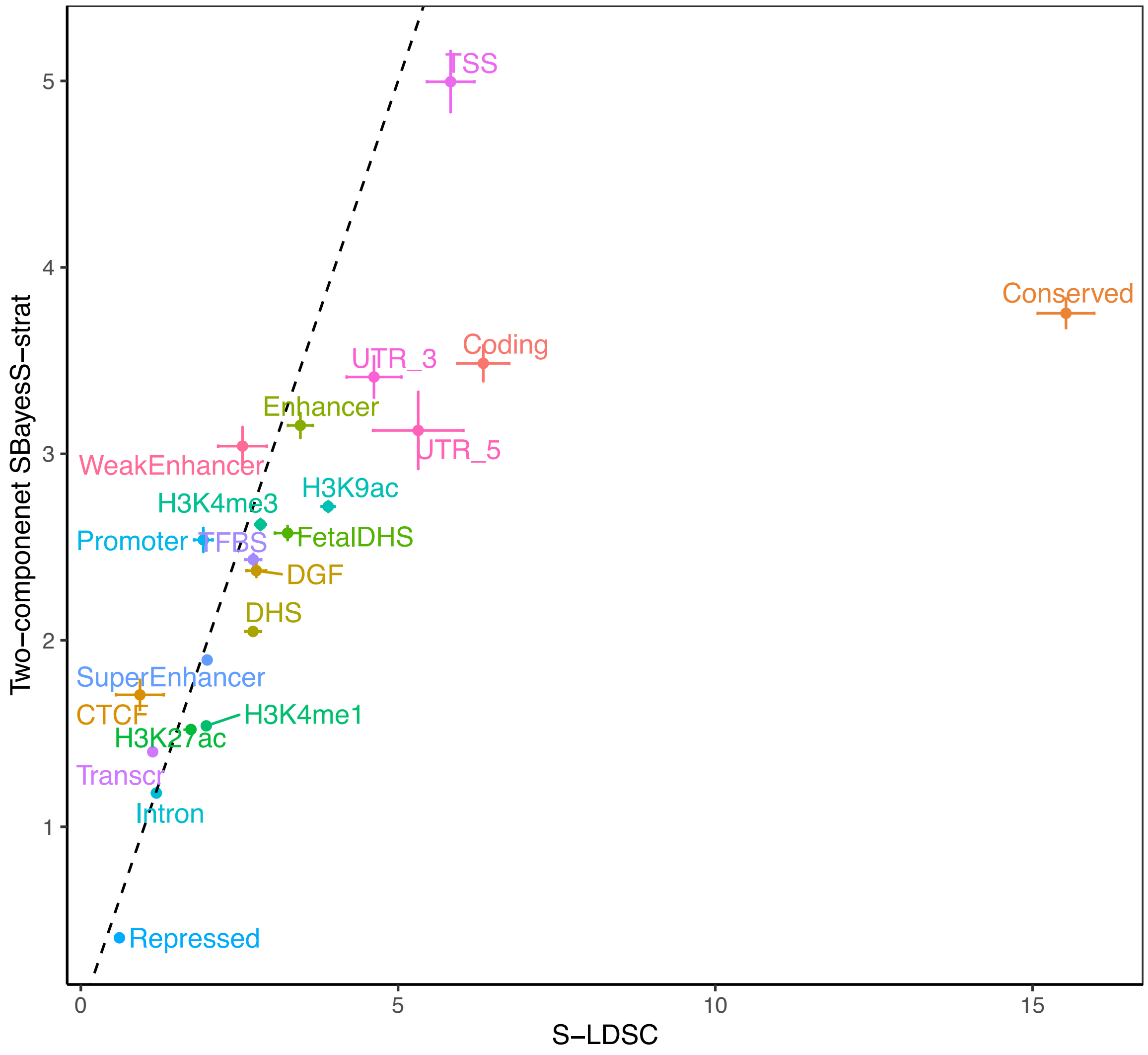

Supplementary Figure 25 Comparison between SBayesS-strat and S-LDSC in per-SNP heritability enrichment using 21 annotation categories from LDSC baseline model. Results are SBayesS-strat analysis that fitted only two components (SNPs in one annotation as the first component and the other SNPs as the second component) versus S-LDSC fitted all annotations. We ran S-LDSC with the same annotations and GWAS summary statistics as used in the analysis above and LD data from the 1000 Genomes Project (the default setting of S-LDSC). Each bar indicates the standard error of the mean. The dashed line shows y=x.

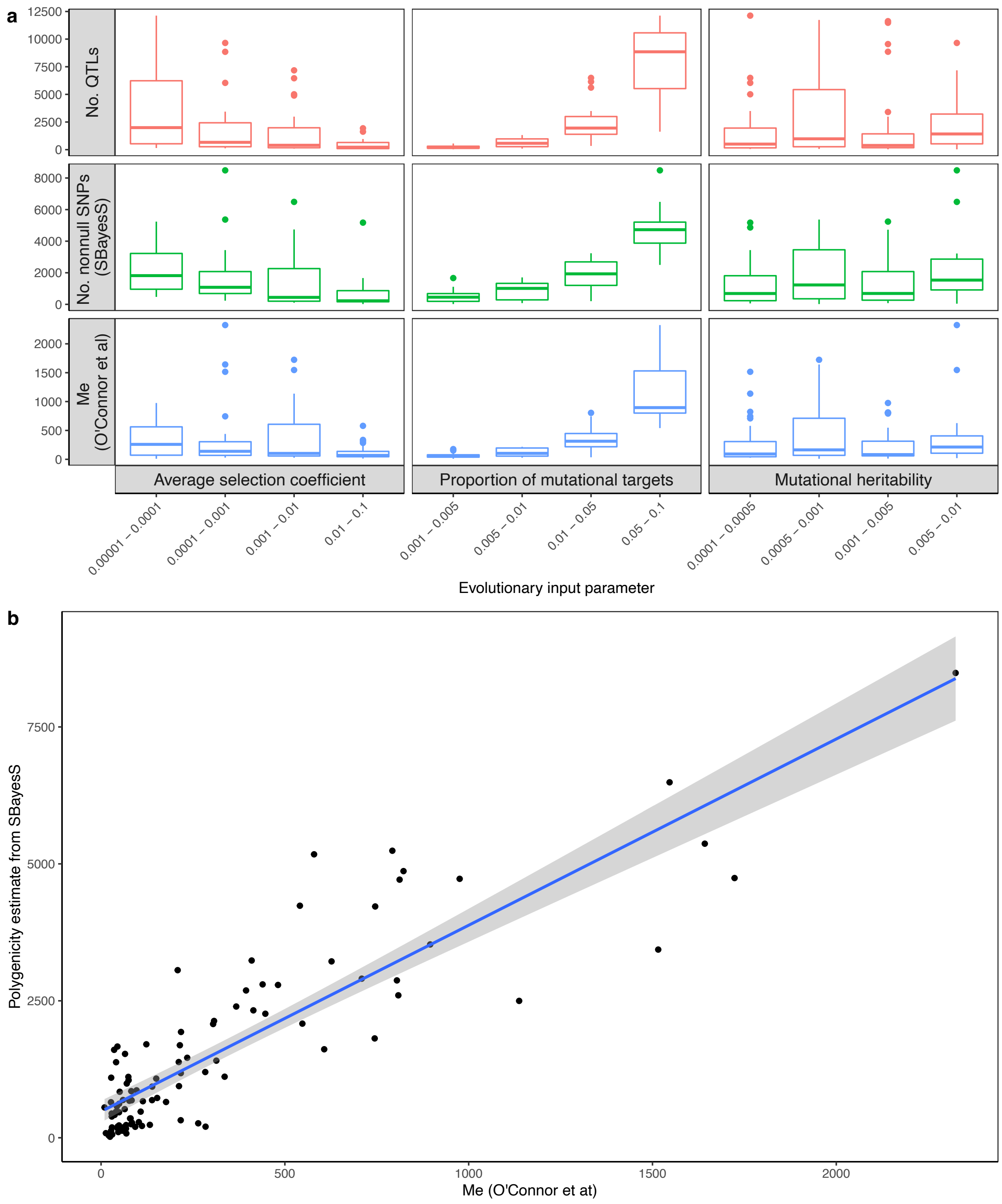

Supplementary Figure 26 Comparison of the polygenicity parameter defined in our study (π) and that in O'Connor et al (M_e) in our forward simulations under negative selection. a) Both π and M_e changed with the total number of causal variants in the simulation. According to the definition in O'Connor et al, $M_e = 3M/\kappa$, $\kappa = E[\beta^4]/E[\beta^2]^2$, where M is the total number of variants (causal variants + 36k SNP markers) and β are the true effect size for the causal variants in per-normalized-genotype units and zero for SNP markers. Each box plot shows the results of 25 independent simulation replicates. The band inside the box is the median, the bottom and top of the box are the first and third quartiles, respectively (Q1 and Q3), and the lower and upper whiskers are Q1 – 1.5 IQR and Q3 + 1.5 IQR, respectively, where IQR = Q3 – Q1.

b) M_e was highly correlated with the polygenicity estimate from SBayesS using 36k common SNP markers (correlation = 0.876, slope of regressing SBayesS estimates on M_e = 3.4). The grey bands around the line represent the standard error of the regression line.

a

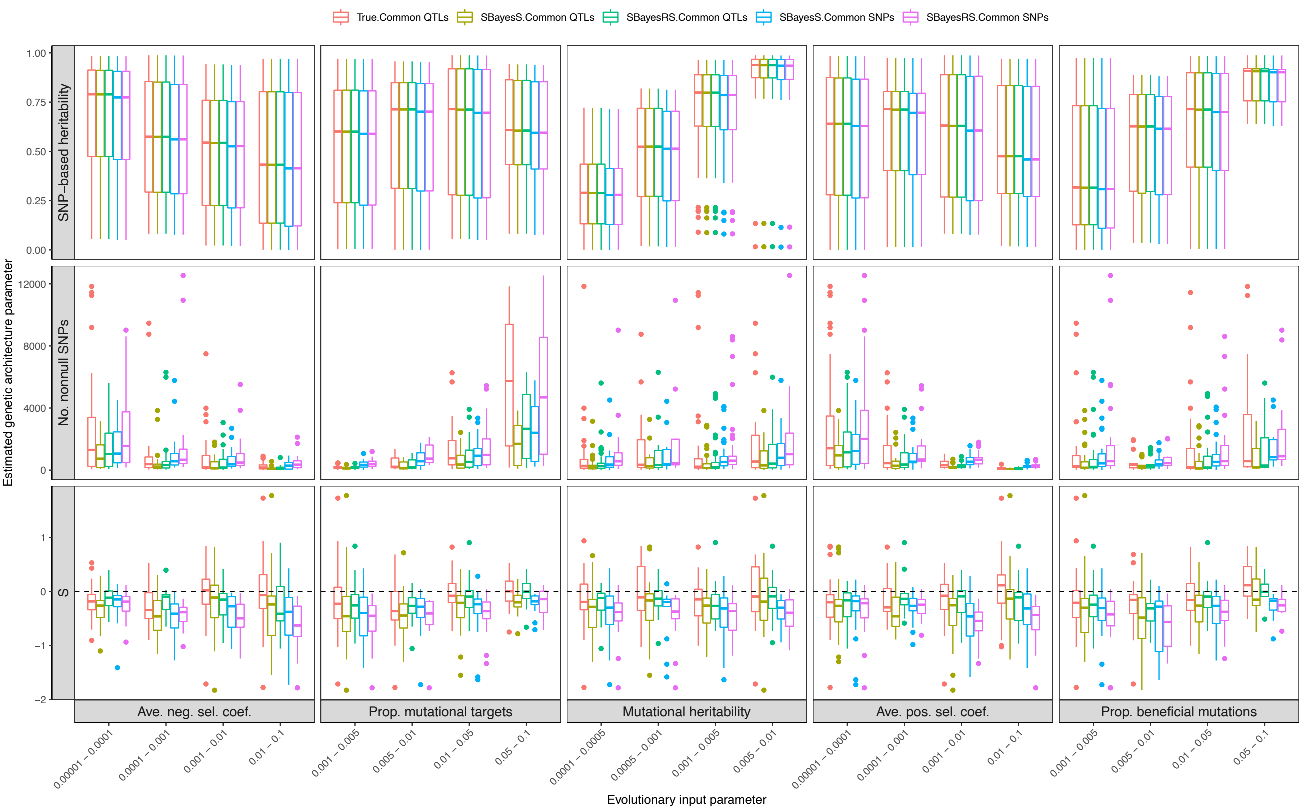

b

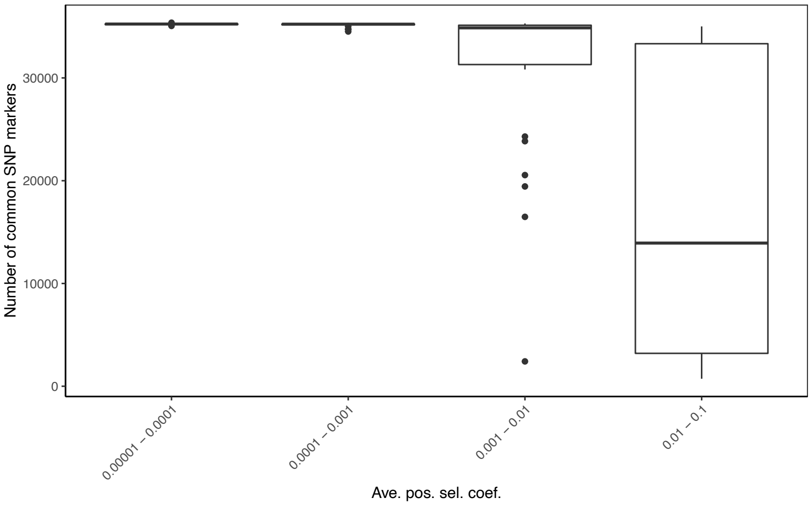

Supplementary Figure 27 Variational patterns of the estimated genetic architecture parameters under both negative and positive selection in evolutionary simulations, when selection coefficients followed a mixture distribution. a) The Simons et al. pleiotropic model with n_t = 1 was used to generate genetic effects. The x-axis shows the values of five input parameters in evolutionary simulations. The y-axis shows the distribution of the genetic architecture parameter estimates, where the polygenicity parameter is represented by the number of nonnull SNPs for better benchmarking. "True, Common QTLs": parameters computed directly from the simulated genetic effects of all common causal variants; "SBayesS, Common

QTLs" (or "SBayesRS, Common QTLs"): SBayesS (or SBayesRS) estimates using the genotype data of the common causal variants and the phenotypes; "SBayesS, Common SNPs" (or "SBayesRS, Common SNPs"): SBayesS (or SBayesRS) estimates using the genotype data of 36k common SNPs and the simulated genetic values. b) The number of common SNP markers decreased with the increased strength of positive selection. Each box plot shows the results of 25 independent simulation replicates. The band inside the box is the median, the bottom and top of the box are the first and third quartiles, respectively (Q1 and Q3), and the lower and upper whiskers are Q1 – 1.5 IQR and Q3 + 1.5 IQR, respectively, where IQR = Q3 – Q1. An apparent discrepancy between the true S value and the estimate from SBayesS/RS using SNP markers was observed when the strength of positive selection increased (row 3, column 4 in panel a). This is likely because the strong positive selection introduced a selective sweep on SNPs in LD with the beneficial mutations of relatively large effects (known as hitchhiking), resulting in a reduced number of common SNPs and a reduced capacity of SNPs to track all the beneficial mutations. In contrast, we did not observe a reduction in the number of common SNPs in the presence of strong negative selection, suggesting background selection has a smaller impact on reducing the SNP diversity than hitchhiking so that the deleterious mutations are better tracked by the SNPs.

a

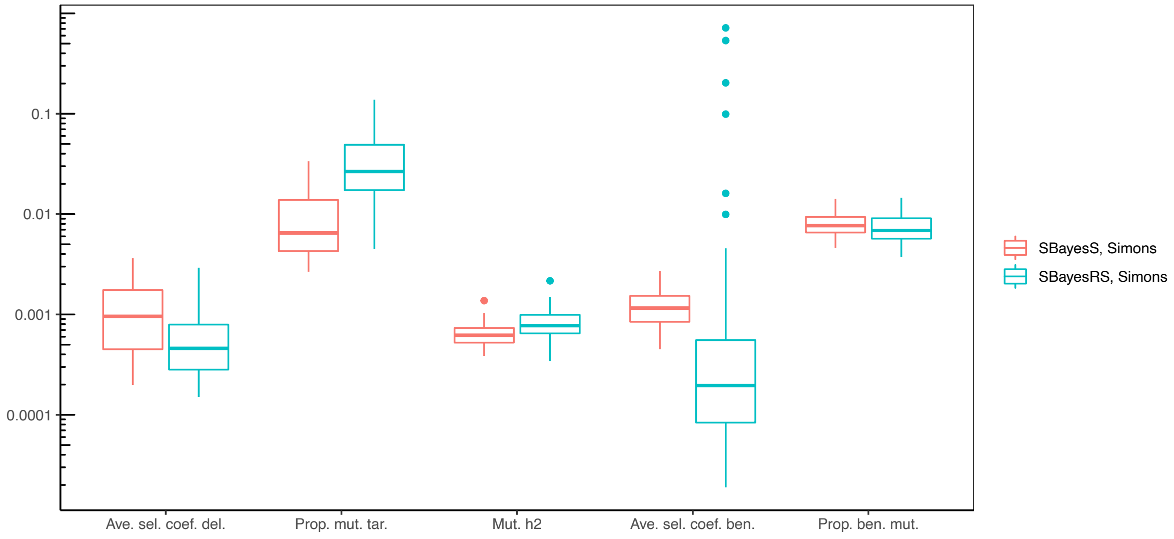

b

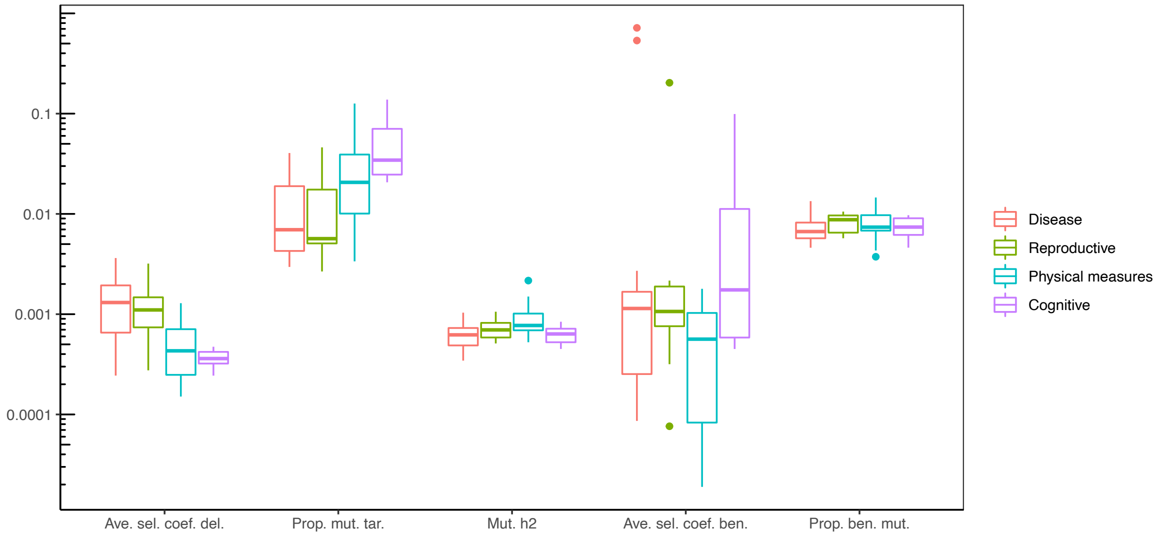

Supplementary Figure 28 Prediction of the evolutionary parameters for 44 complex traits and diseases based on a mixture of negative and positive selection model. a) Distribution of the predicted evolutionary parameters under different methods for estimating genetic architecture parameters (SBayesS and SBayesRS), shown by colours. Each box plot shows the results for 44 complex traits. b) Distribution of predicted evolutionary parameters for five trait categories, shown by colours. Each box plot shows the results for a number of traits in a category, with each trait having two results from analyses using different estimation methods. The band inside the box is the median, the bottom and top of the box are the first and third quartiles, respectively (Q1 and Q3), and the lower and upper whiskers are Q1 – 1.5 IQR and Q3 + 1.5 IQR, respectively, where IQR = Q3 – Q1.

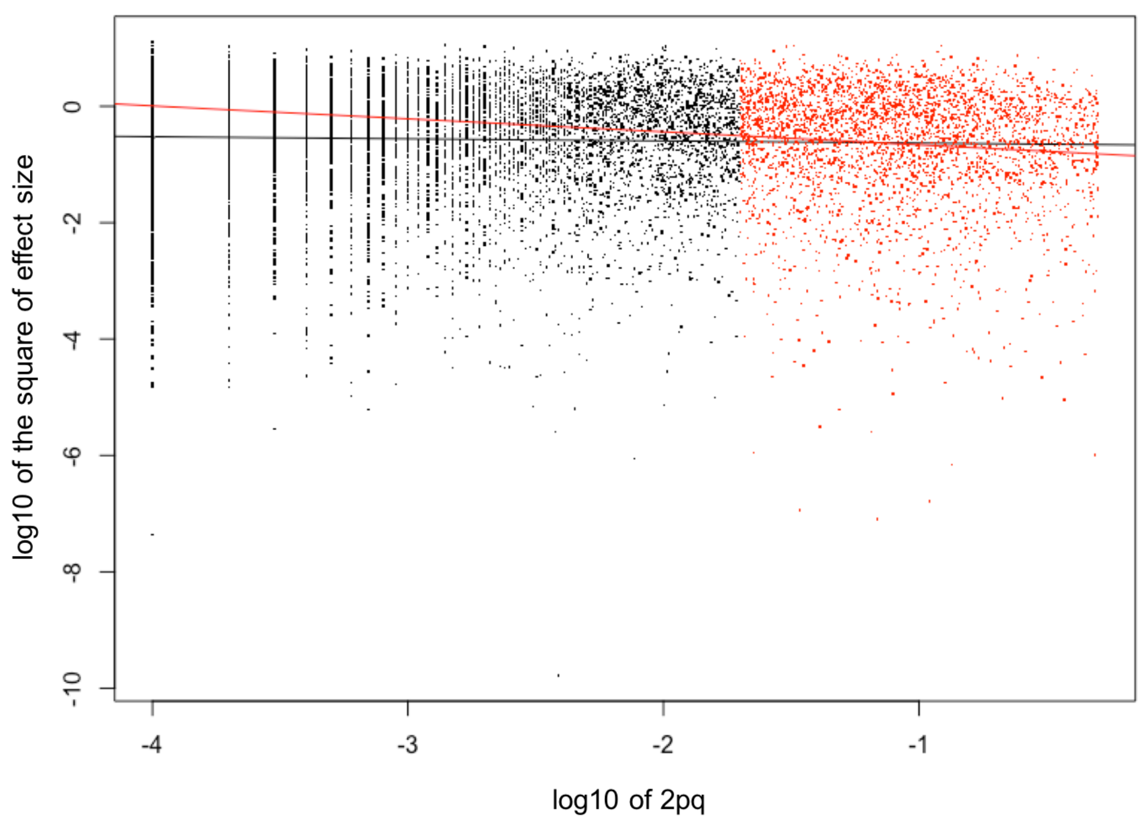

Supplementary Figure 29 Joint distribution of the squared effect size and heterozygosity (2pq) for causal variants in the forward simulation in the presence of negative selection. Red colour shows the causal variants with MAF > 0.01. The line is the regression line for all causal variants (black) or common causal variants (red), which is an estimate of the *S* parameter.

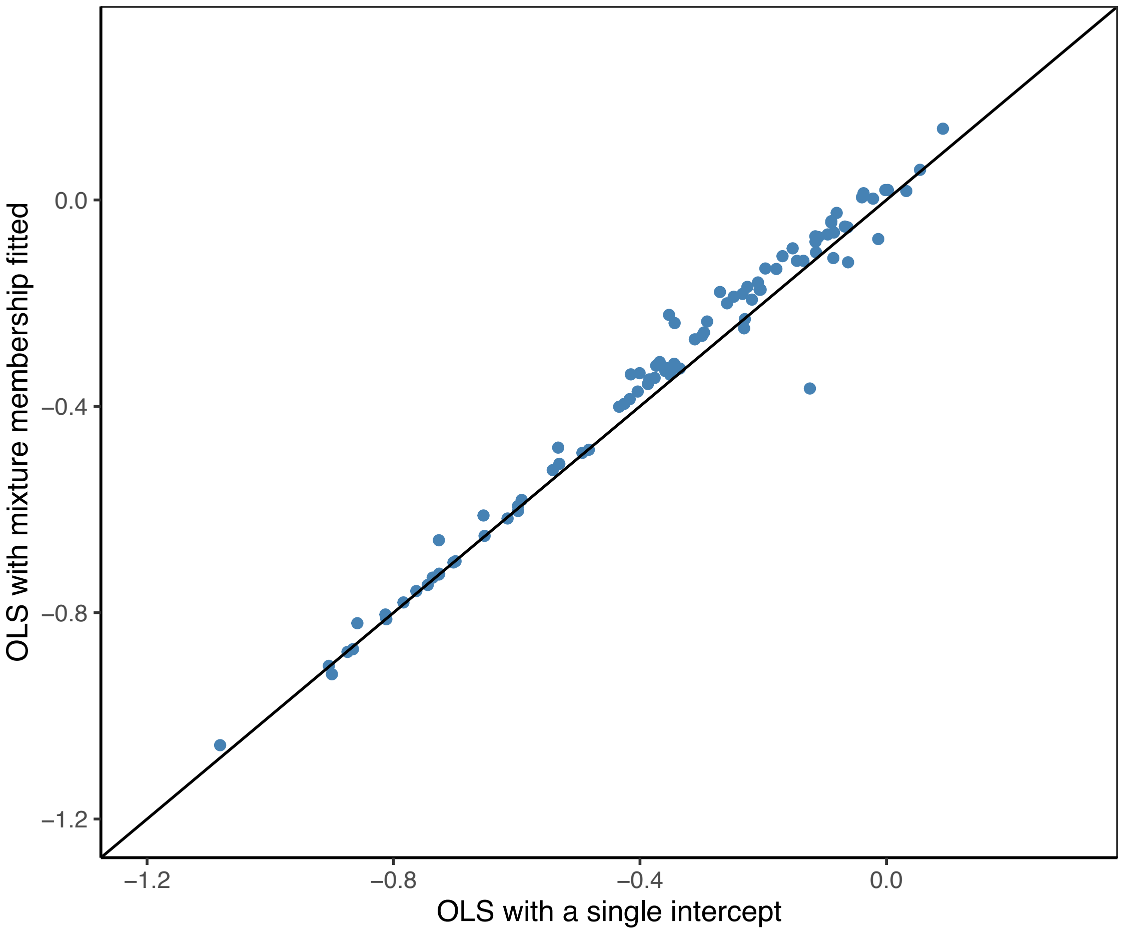

Supplementary Figure 30 Ordinary least squares (OLS) estimate of S from a regression model fitting mixture membership-specific intercepts vs. that from a model fitting a single intercept, when the true model is a mixture distribution.

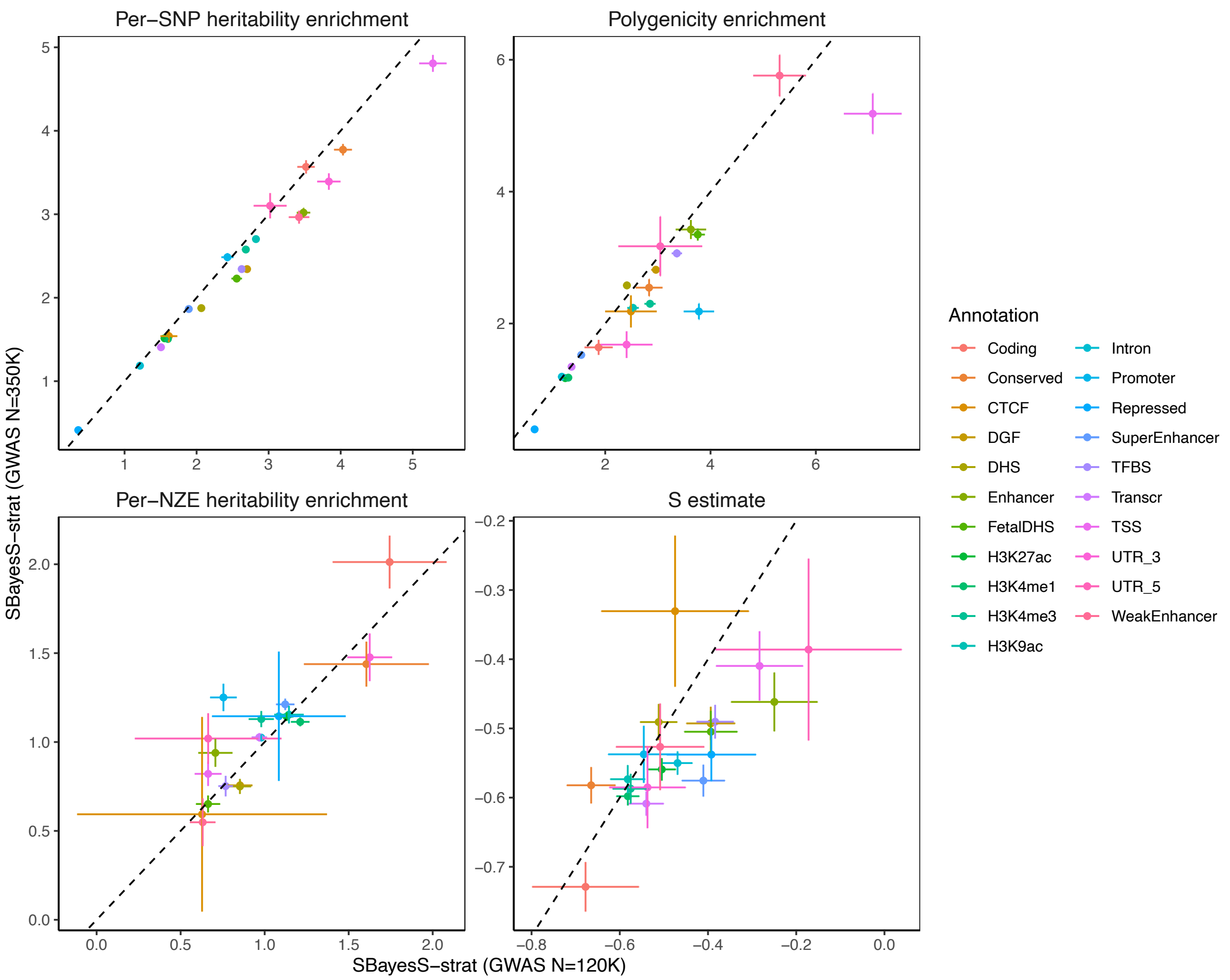

Supplementary Figure 31 Comparison in genetic architecture estimation in 21 functional categories between SBayesS-strat with GWAS sample sizes of 350k and 120k using 1.1M common HapMap3 SNPs. Data are presented as mean values +/- standard errors of the means.

Supplementary Tables

Supplementary Table 1: Estimation of genetic architecture parameters for 35 complex traits (including diseases) in the full UKB data.
The point estimate is the posterior mean and SE is the posterior standard error estimated from the MCMC sample.
The column of "Sample size" for a disease shows the number of cases. The total number of cases and controls is 452,272 for all of the diseases.

Category	Trait	Acronym	Sample size	SNP-based heritability		Polygenicity		S	
				Estimate	SE	Estimate	SE	Estimate	SE
Physical measures	Basal metabolic rate	BMR	339237	0.3143	0.0026	0.0311	0.0009	-0.6209	0.0256
Physical measures	BMI	BMI	344306	0.2619	0.0024	0.0449	0.0016	-0.5461	0.0303
Physical measures	Body fat percentage	BFP	339098	0.2514	0.0024	0.0503	0.0020	-0.5533	0.0308
Physical measures	Hand grip strength left	HGSL	344014	0.1315	0.0021	0.0335	0.0019	-0.5613	0.0448
Physical measures	Hand grip strength right	HGSR	344033	0.1316	0.0021	0.0287	0.0015	-0.4982	0.0451
Physical measures	HCadjBMI	HC	344252	0.2307	0.0025	0.0158	0.0005	-0.5913	0.0294
Physical measures	Heel BMD T-score	HBMD	196375	0.2553	0.0036	0.0058	0.0003	-0.4959	0.0441
Physical measures	Height	Height	344664	0.5452	0.0028	0.0201	0.0004	-0.6529	0.0220
Physical measures	WCadjBMI	WC	344248	0.1734	0.0023	0.0143	0.0006	-0.5899	0.0347
Physical measures	Weight	Weight	344434	0.2763	0.0025	0.0391	0.0013	-0.5620	0.0288
Physical measures	WHRadjBMI	WHR	344228	0.1426	0.0022	0.0120	0.0005	-0.5779	0.0385
Physical measures	Diastolic blood pressure	DBP	322683	0.1410	0.0023	0.0212	0.0010	-0.5417	0.0415
Physical measures	Forced expiratory volume in 1 second	FEV	315184	0.2249	0.0026	0.0249	0.0009	-0.6020	0.0318
Physical measures	Forced vital capacity	FVC	315012	0.2504	0.0026	0.0246	0.0009	-0.6309	0.0287
Physical measures	Peak expiratory flow	PEF	315184	0.1107	0.0022	0.0166	0.0010	-0.5846	0.0475
Physical measures	Pulse rate	PR	325075	0.1384	0.0023	0.0109	0.0005	-0.5578	0.0409
Physical measures	Systolic blood pressure	SBP	322679	0.1471	0.0023	0.0198	0.0009	-0.5603	0.0395
Cognitive	Educational attainment	EA	326945	0.1820	0.0024	0.0478	0.0025	-0.5960	0.0988
Cognitive	Fluid intelligence score	FIS	113198	0.2540	0.0054	0.0384	0.0039	-0.4990	0.0364
Cognitive	Mean time to correctly identify matches	MTCIM	342712	0.0723	0.0019	0.0332	0.0037	-0.4514	0.0738
Cognitive	Neuroticism score	NS	279979	0.1181	0.0023	0.0357	0.0030	-0.4236	0.0604
Reproductive	Age at first live birth	AFLB	125831	0.1966	0.0049	0.0353	0.0043	-0.6681	0.0684
Reproductive	Age at menopause	Mnps	106965	0.0961	0.0049	0.0014	0.0002	-0.6907	0.0818
Reproductive	Age menarche	Mnrch	181335	0.2208	0.0039	0.0143	0.0008	-0.5647	0.0439
Reproductive	Birth weight	BW	196388	0.1046	0.0032	0.0075	0.0006	-0.5175	0.0650
Reproductive	Male pattern baldness	MPB	158696	0.2895	0.0036	0.0035	0.0002	-0.5222	0.0499
Disease	Allergic rhinitis	AR	28041	0.0894	0.0048	0.0049	0.0006	-0.4070	0.0998
Disease	Asthma	Asthma	58479	0.1421	0.0037	0.0060	0.0004	-0.6000	0.0569
Disease	Cancer	Cancer	65534	0.0392	0.0031	0.0027	0.0005	-0.6369	0.0959
Disease	Cardiovascular disease	CD	73856	0.0832	0.0030	0.0110	0.0010	-0.7246	0.0702
Disease	Dyslipidemia	Dyslp	78921	0.1297	0.0036	0.0038	0.0003	-0.8236	0.0477
Disease	Hemorrhoids	Hmrr	26955	0.0738	0.0047	0.0095	0.0017	-0.5752	0.1227
Disease	Hypertensive disease	HD	87650	0.1757	0.0033	0.0167	0.0009	-0.6460	0.0411
Disease	Type 2 diabetes	T2D	27091	0.2291	0.0060	0.0091	0.0006	-0.5642	0.0507
Disease	Varicose veins	VV	13252	0.1722	0.0082	0.0050	0.0006	-0.6059	0.0794

Supplementary Table 2: Estimation of genetic architecture parameters for 9 common diseases from published GWAS data. The point estimate is the posterior mean and SE is the posterior standard error estimated from the MCMC sample.

Disease	Acronym	GWAS summary data set	Cases	Controls	Sample prevalence	Population prevalence	SNP-based heritability		Polygenicity		S	
							Estimate	SE	Estimate	SE	Estimate	SE
Allergic Disease	AD	Ferreira et al (2017 NG)	180129	180709	0.499	0.2	0.0803	0.0026	0.0037	0.0003	-0.5786	0.0772
Bipolar Disorder	BIP	PGC (2018 Cell)	20129	54065	0.271	0.03	0.3439	0.0088	0.0290	0.0050	-0.4198	0.1079
Breast Cancer	BC	Michailidou et al (2017 Nature)	122977	105974	0.537	0.07	0.1338	0.0023	0.0060	0.0003	-0.5553	0.0487
Coronary Artery Disease	CAD	van der Harst et al (2018 Circ Res)	122733	424528	0.224	0.07	0.0713	0.0013	0.0071	0.0004	-0.7452	0.0543
Prostate Cancer	PC	Schumacher et al (2018 NG)	79194	61112	0.564	0.0012	0.0284	0.0010	0.0012	0.0001	-0.3946	0.1053
Schizophrenia	SCZ	PGC (2014 Nature)	36989	113075	0.246	0.01	0.2100	0.0026	0.0462	0.0030	-0.6130	0.0445
Stroke	Stroke	Malik et al (2018 NG)	40585	406111	0.091	0.05	0.0470	0.0026	0.0097	0.0016	-0.6664	0.1355
Ulcerative Colitis	UC	Liu et al (2015 NG)	6968	20464	0.254	0.005	0.1983	0.0104	0.0031	0.0004	-0.6619	0.0904
Vitiligo	Vtlg	Jin et al (2016 NG)	4680	39586	0.106	0.002	0.3818	0.0113	0.0079	0.0006	-0.6960	0.0678

Supplementary Table 3: Classification of 35 UKB traits (including diseases) based on the information provided by the UKB.

Category	Trait	UKB Data-Field code	UKB Category description	UKB Category ID	Remark
Physical measures	Basal metabolic rate	23105	Impedance measures - Anthropometry - Physical measures	100009	
Physical measures	BMI	21001	Body size measures - Anthropometry - Physical measures	100010	
Physical measures	Body fat percentage	23099	Impedance measures - Anthropometry - Physical measures	100009	
Physical measures	Hand grip strength left	46	Hand grip strength - Physical measures	100019	
Physical measures	Hand grip strength right	47	Hand grip strength - Physical measures	100019	
Physical measures	HCadjBMI	49	Body size measures - Anthropometry - Physical measures	100010	Adjusted the phenotype for
Physical measures	Heel BMD T-score	78	Bone-densitometry of heel - Physical measures	100018	
Physical measures	Height	50	Body size measures - Anthropometry - Physical measures	100010	
Physical measures	WCadjBMI	48	Body size measures - Anthropometry - Physical measures	100010	Adjusted the phenotype for
Physical measures	Weight	21002	Body size measures - Anthropometry - Physical measures	100010	
Physical measures	WHRadjBMI	-	-	-	Derived from WC and HC and adjusted for BMI
Physical measures	Diastolic blood pressure	4079	Blood pressure - Physical measures	100011	
Physical measures	Forced expiratory volume in 1 second	3063	Spirometry - Physical measures	100020	
Physical measures	Forced vital capacity	3062	Spirometry - Physical measures	100020	
Physical measures	Peak expiratory flow	3064	Spirometry - Physical measures	100020	
Physical measures	Pulse rate	102	Blood pressure - Physical measures	100011	
Physical measures	Systolic blood pressure	4080	Blood pressure - Physical measures	100011	
Cognitive	Educational attainment	6138	Education - Sociodemographics - Touchscreen	100063	
Cognitive	Fluid intelligence score	20016	Fluid intelligence / reasoning - Cognitive function	100027	
Cognitive	Mean time to correctly identify matches	20023	Reaction time - Cognitive function	100032	
Cognitive	Neuroticism score	20127	Mental health - Psychosocial factors - Touchscreen	100060	
Reproductive	Age at first live birth	2754	Female-specific factors - Sex-specific factors - Touchscreen	708	
Reproductive	Age at menopause	3581	Female-specific factors - Sex-specific factors - Touchscreen	708	
Reproductive	Age at menarche	2714	Female-specific factors - Sex-specific factors - Touchscreen	708	
Reproductive	Birth weight	20022	Early life factors - Verbal interview	708	
Reproductive	Male pattern baldness	2395	Male-specific factors - Sex-specific factors - Touchscreen	708	
Disease	Allergic rhinitis	J30 + 1387	Diagnoses - main ICD10 + secondary ICD10 + self-reported	41202+41204+20002	Phenotype are acquired from self-reported, ICD 10 main diagnosis and ICD 10 secondary diagnosis
Disease	Asthma	J45 + 1111	Diagnoses - main ICD10 + secondary ICD10 + self-reported	41202+41204+20002	
Disease	Cancer	C01, C03, C04, C05, C06, C07, C08, C09, C10, C11, C12, C13, C14, C15, C16, C18, C22, C23, C24, C25, C33, C43, C44, C45, C46, C47, C48, C49, C50, C53, C54, C55, C56, C57, C58, C59, C60, C61, C64, C67, C71, C72, C76, C77, C78, C79, C80, C81, C82, C83, C84, C85, C91, C92, C94, C96, D48, K22.7	Diagnoses - main ICD10 + secondary ICD10	41202+41204	
Disease	Cardiovascular disease	I01, I09, I11, I13, I20, I21, I22, I23, I24, I25, I42, I43, I44, I45, I46, I47, I48, I49, I50, I60, I61, I62, I63, I64, I65, I66, I67, I68, I69, I71 + 1074, 1075, 1066, 1485, 1076, 1471, 1483, 1484, 1486, 1487, 1588, 1426, 1479, 1086, 1491, 1083,1082, 1425, 1592, 1068, 1094, 1079, 1492, 1591, 1583, 1077	Diagnoses - main ICD10 + secondary ICD10 + self-reported	41202+41204+20002	
Disease	Dyslipidemia	E78 + 1473	Diagnoses - main ICD10 + secondary ICD10 + self-reported	41202+41204+20002	
Disease	Hemorrhoids	I84 + 1505	Diagnoses - main ICD10 + secondary ICD10 + self-reported	41202+41204+20002	
Disease	Hypertensive disease	I10-I13 + 1065, 1072,1073	Diagnoses - main ICD10 + secondary ICD10 + self-reported	41202+41204+20002	
Disease	Type 2 diabetes	E11 + 1223	Diagnoses - main ICD10 + secondary ICD10 + self-reported	41202+41204+20002	
Disease	Varicose veins	I26, I70, I82 + 1067, 1087 , 1088	Diagnoses - main ICD10 + secondary ICD10 + self-reported	41202+41204+20002	

Supplementary Table 4: Estimation of genetic architecture parameters for 110 quantitative traits from Neale Lab GWAS summary data.

Phenotype Code	Phenotype Description	Sample size	SNP-based heritability		Polygenicity		S	
			Estimate	SE	Estimate	SE	Estimate	SE
102_irnt	Pulse rate, automated reading	340162	0.1505	0.0023	0.0112	0.0005	-0.5682	0.0375
1438_irnt	Bread intake	353030	0.0503	0.0017	0.0373	0.0075	-0.5239	0.0886
1488_irnt	Tea intake	349376	0.0570	0.0018	0.0170	0.0015	-0.3777	0.0877
20015_irnt	Sitting height	360066	0.3711	0.0026	0.0188	0.0005	-0.6428	0.0244
20150_irnt	Forced expiratory volume in 1-second (FEV1), Best measure	272338	0.2252	0.0028	0.0239	0.0009	-0.5923	0.0348
20151_irnt	Forced vital capacity (FVC), Best measure	272338	0.2571	0.0029	0.0232	0.0008	-0.6254	0.0306
20153_irnt	Forced expiratory volume in 1-second (FEV1), predicted	117241	0.4595	0.0064	0.0104	0.0004	-0.6217	0.0363
20154_irnt	Forced expiratory volume in 1-second (FEV1), predicted percentage	117241	0.2109	0.0053	0.0186	0.0014	-0.4829	0.0726
2139_irnt	Age first had sexual intercourse	317694	0.1636	0.0023	0.0408	0.0023	-0.5570	0.0399
2217_irnt	Age started wearing glasses or contact lenses	310992	0.0755	0.0021	0.0148	0.0011	-0.5360	0.0650
23098_irnt	Weight	354838	0.2821	0.0025	0.0392	0.0013	-0.5620	0.0275
23100_irnt	Whole body fat mass	354244	0.2530	0.0024	0.0463	0.0018	-0.5138	0.0300
23101_irnt	Whole body fat-free mass	354808	0.3260	0.0026	0.0278	0.0008	-0.6397	0.0240
23102_irnt	Whole body water mass	354834	0.3254	0.0026	0.0280	0.0008	-0.6382	0.0240
23104_irnt	Body mass index (BMI)	354831	0.2648	0.0024	0.0477	0.0017	-0.5347	0.0297
23106_irnt	Impedance of whole body	354795	0.2790	0.0025	0.0337	0.0011	-0.6082	0.0265
23107_irnt	Impedance of leg (right)	354817	0.2602	0.0025	0.0310	0.0010	-0.5915	0.0280
23108_irnt	Impedance of leg (left)	354811	0.2592	0.0025	0.0310	0.0010	-0.5878	0.0273
23109_irnt	Impedance of arm (right)	354792	0.2540	0.0024	0.0323	0.0011	-0.5761	0.0284
23110_irnt	Impedance of arm (left)	354807	0.2546	0.0024	0.0330	0.0011	-0.5908	0.0283
23111_irnt	Leg fat percentage (right)	354811	0.2382	0.0023	0.0528	0.0022	-0.5211	0.0313
23112_irnt	Leg fat mass (right)	354807	0.2465	0.0024	0.0469	0.0019	-0.5126	0.0302
23113_irnt	Leg fat-free mass (right)	354798	0.3037	0.0026	0.0302	0.0009	-0.6275	0.0252
23114_irnt	Leg predicted mass (right)	354798	0.3033	0.0026	0.0302	0.0009	-0.6305	0.0254
23115_irnt	Leg fat percentage (left)	354791	0.2391	0.0023	0.0514	0.0022	-0.5326	0.0307
23116_irnt	Leg fat mass (left)	354788	0.2477	0.0024	0.0460	0.0018	-0.5172	0.0314
23117_irnt	Leg fat-free mass (left)	354771	0.3016	0.0025	0.0309	0.0009	-0.6239	0.0251
23118_irnt	Leg predicted mass (left)	354766	0.3011	0.0025	0.0309	0.0009	-0.6227	0.0253
23119_irnt	Arm fat percentage (right)	354760	0.2368	0.0023	0.0478	0.0020	-0.5235	0.0311
23120_irnt	Arm fat mass (right)	354736	0.2451	0.0024	0.0443	0.0017	-0.5172	0.0309
23121_irnt	Arm fat-free mass (right)	354732	0.2931	0.0025	0.0287	0.0009	-0.6329	0.0255
23122_irnt	Arm predicted mass (right)	354726	0.2923	0.0025	0.0287	0.0009	-0.6330	0.0253
23123_irnt	Arm fat percentage (left)	354707	0.2379	0.0023	0.0481	0.0020	-0.5159	0.0321
23124_irnt	Arm fat mass (left)	354673	0.2451	0.0024	0.0451	0.0017	-0.5263	0.0301
23125_irnt	Arm fat-free mass (left)	354668	0.2909	0.0025	0.0295	0.0009	-0.6293	0.0255
23126_irnt	Arm predicted mass (left)	354653	0.2905	0.0025	0.0296	0.0009	-0.6295	0.0255
23127_irnt	Trunk fat percentage	354619	0.2330	0.0023	0.0460	0.0018	-0.5410	0.0306
23128_irnt	Trunk fat mass	354597	0.2520	0.0024	0.0453	0.0017	-0.5219	0.0297
23129_irnt	Trunk fat-free mass	354530	0.3212	0.0026	0.0250	0.0007	-0.6382	0.0247
23130_irnt	Trunk predicted mass	354494	0.3194	0.0026	0.0250	0.0007	-0.6394	0.0249
2764_irnt	Age at last live birth	131806	0.1010	0.0043	0.0319	0.0101	-0.6399	0.0987
30000_irnt	White blood cell (leukocyte) count	350470	0.1998	0.0024	0.0121	0.0005	-0.6241	0.0321
30010_irnt	Red blood cell (erythrocyte) count	350475	0.2357	0.0024	0.0089	0.0003	-0.5954	0.0340
30020_irnt	Haemoglobin concentration	350474	0.1813	0.0022	0.0082	0.0003	-0.6303	0.0375
30030_irnt	Haematocrit percentage	350475	0.1710	0.0022	0.0087	0.0003	-0.6006	0.0372
30040_irnt	Mean corpuscular volume	350473	0.2582	0.0020	0.0032	0.0001	-0.6632	0.0515
30060_irnt	Mean corpuscular haemoglobin concentration	350468	0.0468	0.0013	0.0008	0.0001	-0.7779	0.1022
30070_irnt	Red blood cell (erythrocyte) distribution width	350473	0.1939	0.0019	0.0026	0.0001	-0.5763	0.0595
30080_irnt	Platelet count	350474	0.2820	0.0022	0.0049	0.0002	-0.5876	0.0389
30090_irnt	Platelet crit	350471	0.2441	0.0023	0.0059	0.0002	-0.6417	0.0392
30120_irnt	Lymphocyte count	349856	0.1989	0.0023	0.0100	0.0004	-0.5674	0.0351
30130_irnt	Monocyte count	349856	0.2138	0.0021	0.0042	0.0002	-0.4001	0.0464
30140_irnt	Neutrophill count	349856	0.1704	0.0023	0.0087	0.0004	-0.6591	0.0338
30180_irnt	Lymphocyte percentage	349861	0.1603	0.0021	0.0073	0.0003	-0.5850	0.0379
30190_irnt	Monocyte percentage	349861	0.1917	0.0018	0.0026	0.0001	-0.3677	0.0549
30200_irnt	Neutrophill percentage	349861	0.1472	0.0021	0.0068	0.0003	-0.6293	0.0381
30210_irnt	Eosinophill percentage	349861	0.1945	0.0021	0.0049	0.0002	-0.6009	0.0407
30220_irnt	Basophill percentage	349861	0.0427	0.0013	0.0011	0.0001	-0.6336	0.0819
30240_irnt	Reticulocyte percentage	344728	0.1922	0.0024	0.0050	0.0002	-0.6802	0.0417
30250_irnt	Reticulocyte count	344729	0.2009	0.0024	0.0065	0.0003	-0.6711	0.0386
30280_irnt	Immature reticulocyte fraction	344728	0.1290	0.0020	0.0027	0.0002	-0.6708	0.0513
30290_irnt	High light scatter reticulocyte percentage	344729	0.2077	0.0024	0.0058	0.0003	-0.7186	0.0376
30300_irnt	High light scatter reticulocyte count	344729	0.2111	0.0024	0.0068	0.0003	-0.6987	0.0361
30510_irnt	Creatinine (enzymatic) in urine	350812	0.0671	0.0018	0.0304	0.0032	-0.4226	0.0734
30520_irnt	Potassium in urine	350053	0.0425	0.0017	0.0297	0.0063	-0.4672	0.1001
30530_irnt	Sodium in urine	350061	0.0744	0.0018	0.0318	0.0032	-0.4729	0.0669
3143_irnt	Ankle spacing width	206589	0.3142	0.0038	0.0167	0.0006	-0.6304	0.0319
3144_irnt	Heel Broadband ultrasound attenuation, direct entry	206576	0.2585	0.0035	0.0060	0.0003	-0.5104	0.0430
3147_irnt	Heel quantitative ultrasound index (QUI), direct entry	206589	0.2886	0.0036	0.0062	0.0003	-0.5102	0.0423
3148_irnt	Heel bone mineral density (BMD)	206496	0.2902	0.0036	0.0062	0.0003	-0.5027	0.0426
3761_irnt	Age hay fever, rhinitis or eczema diagnosed	72232	0.0967	0.0073	0.0026	0.0004	-0.8569	0.0859
3786_irnt	Age asthma diagnosed	36955	0.1065	0.0124	0.0012	0.0003	-0.4404	0.1889
399_irnt	Number of incorrect matches in round	360686	0.0536	0.0017	0.0274	0.0036	-0.4154	0.0847
400_irnt	Time to complete round	354739	0.0910	0.0019	0.0347	0.0029	-0.5390	0.0542
404_irnt	Duration to first press of snap-button in each round	358500	0.0889	0.0019	0.0331	0.0026	-0.4543	0.0605
4100_irnt	Ankle spacing width (left)	114630	0.3123	0.0061	0.0123	0.0007	-0.6002	0.0444
4101_irnt	Heel broadband ultrasound attenuation (left)	114625	0.2388	0.0055	0.0038	0.0003	-0.4869	0.0603
4104_irnt	Heel quantitative ultrasound index (QUI), direct entry (left)	114630	0.2692	0.0055	0.0040	0.0003	-0.5005	0.0571
4105_irnt	Heel bone mineral density (BMD) (left)	114561	0.2706	0.0056	0.0039	0.0003	-0.5044	0.0563
4106_irnt	Heel bone mineral density (BMD) T-score, automated (left)	114630	0.2692	0.0056	0.0040	0.0003	-0.5008	0.0567
4119_irnt	Ankle spacing width (right)	114614	0.3070	0.0060	0.0121	0.0007	-0.5788	0.0454
4120_irnt	Heel broadband ultrasound attenuation (right)	114609	0.2368	0.0053	0.0035	0.0002	-0.5772	0.0571
4123_irnt	Heel quantitative ultrasound index (QUI), direct entry (right)	114614	0.2694	0.0056	0.0037	0.0002	-0.5475	0.0551
4124_irnt	Heel bone mineral density (BMD) (right)	114552	0.2709	0.0055	0.0037	0.0002	-0.5519	0.0546
4125_irnt	Heel bone mineral density (BMD) T-score, automated (right)	114614	0.2694	0.0056	0.0037	0.0002	-0.5439	0.0553
48_irnt	Waist circumference	360564	0.2151	0.0022	0.0440	0.0018	-0.5108	0.0331
49_irnt	Hip circumference	360521	0.2316	0.0023	0.0376	0.0014	-0.5381	0.0305
5084_irnt	Spherical power (right)	77983	0.3132	0.0084	0.0072	0.0005	-0.5600	0.0557
5085_irnt	Spherical power (left)	77739	0.3100	0.0085	0.0070	0.0005	-0.5887	0.0540
5096_irnt	3mm weak meridian (left)	75398	0.3787	0.0084	0.0057	0.0004	-0.5773	0.0503
5097_irnt	6mm weak meridian (left)	65551	0.4059	0.0097	0.0058	0.0004	-0.6037	0.0499
5098_irnt	6mm weak meridian (right)	66256	0.4103	0.0094	0.0059	0.0004	-0.6093	0.0506
5099_irnt	3mm weak meridian (right)	75410	0.3853	0.0084	0.0059	0.0004	-0.5697	0.0508
5116_irnt	3mm cylindrical power (right)	75410	0.0789	0.0063	0.0054	0.0011	0.0894	0.2621
5119_irnt	3mm cylindrical power (left)	75398	0.0786	0.0064	0.0055	0.0011	-0.2686	0.2346
5132_irnt	3mm strong meridian (right)	75410	0.3694	0.0083	0.0056	0.0003	-0.5123	0.0536
5133_irnt	6mm strong meridian (right)	66256	0.4010	0.0094	0.0056	0.0004	-0.5585	0.0542
5134_irnt	6mm strong meridian (left)	65551	0.4025	0.0097	0.0057	0.0004	-0.5535	0.0533
5135_irnt	3mm strong meridian (left)	75398	0.3628	0.0083	0.0055	0.0004	-0.5198	0.0525
5254_irnt	Intra-ocular pressure, corneal-compensated (right)	76630	0.1485	0.0072	0.0042	0.0005	-0.5365	0.0868
5255_irnt	Intra-ocular pressure, Goldmann-correlated (right)	76630	0.2116	0.0077	0.0056	0.0005	-0.4560	0.0776
5256_irnt	Corneal hysteresis (right)	76630	0.1939	0.0072	0.0028	0.0003	-0.6372	0.0702
5257_irnt	Corneal resistance factor (right)	76630	0.2528	0.0076	0.0033	0.0003	-0.6076	0.0631
5262_irnt	Intra-ocular pressure, corneal-compensated (left)	76510	0.1531	0.0076	0.0049	0.0006	-0.5963	0.0849
5263_irnt	Intra-ocular pressure, Goldmann-correlated (left)	76510	0.2242	0.0076	0.0061	0.0005	-0.5166	0.0694
5264_irnt	Corneal hysteresis (left)	76510	0.1792	0.0072	0.0027	0.0003	-0.5867	0.0744
5265_irnt	Corneal resistance factor (left)	76510	0.2430	0.0075	0.0033	0.0003	-0.5730	0.0680
5983_irnt	ECG, heart rate	53777	0.0997	0.0096	0.0034	0.0007	-0.8084	0.1199
699_irnt	Length of time at current address	352690	0.0278	0.0016	0.0167	0.0036	-0.3322	0.1929
874_irnt	Duration of walks	308989	0.0463	0.0018	0.0332	0.0081	-0.3579	0.1174

Supplementary Table 5: Estimation of genetic architecture parameters for 21 functional genomic categories from the LDSC baseline model meta-analysed over 44 UKB complex traits and diseases.

Results are from the two-component model where the SNPs in one annotation are fitted as one group and the other SNPs are fitted as the other group. The point estimate is the posterior median and SE is the posterior standard error estimated from the MCMC sample.
Polygenicity is the proportion of SNPs with nonzero effects among all SNPs in the annotation.

Annotation	Number of SNPs	Fraction of SNPs	SNP-based heritability		Polygenicity		S	
			median	s.e.m.	median	s.e.m.	median	s.e.m.
Coding_UCSC	33338	0.033	0.1158	0.0034	0.0513	0.0031	-0.7392	0.0411
Conserved_LindbladToh	53319	0.053	0.1993	0.0045	0.0686	0.0044	-0.5741	0.0390
CTCF_Hoffman	27260	0.027	0.0464	0.0024	0.0923	0.0075	-0.4981	0.0950
DGF_ENCODE	188706	0.188	0.4463	0.0074	0.0720	0.0024	-0.4983	0.0272
DHS_Trynka	254685	0.254	0.5195	0.0066	0.0669	0.0021	-0.5100	0.0254
Enhancer_Hoffman	54119	0.054	0.1700	0.0039	0.0803	0.0044	-0.4677	0.0391
FetalDHS_Trynka	132853	0.132	0.3408	0.0058	0.0973	0.0034	-0.4594	0.0336
H3K27ac_Hnisz	456432	0.455	0.6923	0.0052	0.0293	0.0005	-0.5646	0.0176
H3K4me1_Trynka	548165	0.546	0.8418	0.0061	0.0333	0.0006	-0.5929	0.0155
H3K4me3_Trynka	165655	0.165	0.4328	0.0057	0.0609	0.0016	-0.5805	0.0222
H3K9ac_Trynka	162379	0.162	0.4398	0.0055	0.0563	0.0017	-0.5862	0.0235
Intron_UCSC	436629	0.435	0.5139	0.0047	0.0237	0.0006	-0.5304	0.0193
Promoter_UCSC	56916	0.057	0.1440	0.0039	0.0477	0.0027	-0.5188	0.0409
Repressed_Hoffman	438171	0.437	0.1767	0.0052	0.0097	0.0011	-0.5735	0.0575
SuperEnhancer_Hnisz	195544	0.195	0.3693	0.0040	0.0298	0.0008	-0.5239	0.0262
TFBS_ENCODE	170829	0.170	0.4142	0.0062	0.0662	0.0021	-0.5399	0.0296
Transcr_Hoffman	367588	0.366	0.5136	0.0054	0.0339	0.0010	-0.6069	0.0202
TSS_Hoffman	20812	0.021	0.1036	0.0035	0.1184	0.0059	-0.3609	0.0657
UTR_3_UCSC	22031	0.022	0.0750	0.0026	0.0636	0.0060	-0.5122	0.0549
UTR_5_UCSC	9223	0.009	0.0287	0.0020	0.0977	0.0088	-0.5521	0.1102
WeakEnhancer_Hoffman	28247	0.028	0.0856	0.0030	0.1350	0.0069	-0.5917	0.0540

References

[1] Jian Yang, Teresa Ferreira, Andrew P Morris, Sarah E Medland, Genetic of Consortium, DIAbetes Consortium, Pamela AF Madden, Andrew C Heath, Nicholas G Martin, Grant W Montgomery, Michael N Weedon, Ruth J Loos, Timothy M Frayling, McCarthy, Mark I, Joel N Hirschhorn, Michael E Goddard, Peter M Visscher. Conditional and joint multiple-SNP analysis of GWAS summary statistics identifies additional variants influencing complex traits. *Nat Genet*, 44(4):369, 2012.

[2] Xiang Zhu, Matthew Stephens. Bayesian large-scale multiple regression with summary statistics from genome-wide association studies. *Ann Appl Statistics*, 11(3):1561–1592, 2017.

[3] Jian Zeng, Ronald Vlaming, Yang Wu, Matthew R Robinson, Lloyd-Jones, Luke R, Loic Yengo, Chloe X Yap, Angli Xue, Julia Sidorenko, McRae, Allan F, Joseph E Powell, Grant W Montgomery, Andres Metspalu, Tonu Esko, Greg Gibson, Naomi R Wray, Peter M Visscher, Jian Yang. Signatures of negative selection in the genetic architecture of human complex traits. *Nat Genet*, strona 1, 2018.

[4] Lloyd-Jones, Luke R, Jian Zeng, Julia Sidorenko, Loic Yengo, Gerhard Moser, Kathryn E Kemper, Huanwei Wang, Zhili Zheng, Reedik Magi, Tonu Esko, Andres Metspalu, Naomi R Wray, Michael E Goddard, Jian Yang, Peter M Visscher. Improved polygenic prediction by bayesian multiple regression on summary statistics. *Biorxiv*, strona 522961, 2019.

[5] Mario PL Calus. Right-hand-side updating for fast computing of genomic breeding values. 46(1):24, 2014.

[6] Jian Yang, Jian Zeng, Michael E Goddard, Naomi R Wray, Peter M Visscher. Concepts, estimation and interpretation of SNP-based heritability. *Nat Genet*, 49(9):1304–1310, 2017.

[7] O'Connor, Luke J, Alkes L Price. Distinguishing genetic correlation from causation across 52 diseases and complex traits. strony 1728–1734, 2018.
