# Supplementary material for: Widespread signatures of natural selection across human complex traits and functional genomic categories
Source: Nat Commun. 2021 Feb 19;12:1164. doi: 10.1038/s41467-021-21446-3 (PMC7896067; doi:10.1038/s41467-021-21446-3)
Supplement: Supplementary file 3 — Reporting Summary [file 41467_2021_21446_MOESM3_ESM.pdf]

nature research

Corresponding author(s): Jian Zeng and Jian Yang

Last updated by author(s): Dec 31, 2020

Reporting Summary

Nature Research wishes to improve the reproducibility of the work that we publish. This form provides structure for consistency and transparency in reporting. For further information on Nature Research policies, see Authors & Referees and the Editorial Policy Checklist .

Statistics

For all statistical analyses, confirm that the following items are present in the figure legend, table legend, main text, or Methods section.

n/a	Confirmed	
☐	☒	The exact sample size (*n*) for each experimental group/condition, given as a discrete number and unit of measurement
☒	☐	A statement on whether measurements were taken from distinct samples or whether the same sample was measured repeatedly
☐	☒	The statistical test(s) used AND whether they are one- or two-sided<br>*Only common tests should be described solely by name; describe more complex techniques in the Methods section.*
☐	☒	A description of all covariates tested
☐	☒	A description of any assumptions or corrections, such as tests of normality and adjustment for multiple comparisons
☐	☒	A full description of the statistical parameters including central tendency (e.g. means) or other basic estimates (e.g. regression coefficient) AND variation (e.g. standard deviation) or associated estimates of uncertainty (e.g. confidence intervals)
☐	☒	For null hypothesis testing, the test statistic (e.g. *F*, *t*, *r*) with confidence intervals, effect sizes, degrees of freedom and *P* value noted<br>*Give P values as exact values whenever suitable.*
☐	☒	For Bayesian analysis, information on the choice of priors and Markov chain Monte Carlo settings
☒	☐	For hierarchical and complex designs, identification of the appropriate level for tests and full reporting of outcomes
☐	☒	Estimates of effect sizes (e.g. Cohen's *d*, Pearson's *r*), indicating how they were calculated

Our web collection on statistics for biologists contains articles on many of the points above.

Software and code

Policy information about availability of computer code

Data collection	We used PLINK v1.90beta to read and extract the genotype data for the traits in the UK Biobank resource. The GWAS summary data from Neale Lab and the other published studies were directly downloaded from the provided websites. We also used SLiM v3.3 to generate data from the forward simulation. The functional genomic annotation data were collected from the baseline-LD annotations (URLs).
Data analysis	We used PLINK v1.90beta for quality control and GWAS analyses of the UKB data. We also used GCTA v1.92.2beta to estimate genetic relationships between individuals from SNP data. We implemented the SBayesS methods (including the MCMC algorithm) in GCTB v2.02 for the analysis of GWAS summary data. The computer code of GCTB is freely available at http://cnsgenomics.com/software/gctb.

For manuscripts utilizing custom algorithms or software that are central to the research but not yet described in published literature, software must be made available to editors/reviewers. We strongly encourage code deposition in a community repository (e.g. GitHub). See the Nature Research guidelines for submitting code & software for further information.

Data

Policy information about availability of data

All manuscripts must include a data availability statement. This statement should provide the following information, where applicable:

- Accession codes, unique identifiers, or web links for publicly available datasets
- A list of figures that have associated raw data
- A description of any restrictions on data availability

This study makes use of individual-level genotype and phenotype data from UK Biobank Resource (application number: 12505) as well as GWAS summary data and functional genomic annotation data from the public domain. UK Biobank: https://www.ukbiobank.ac.uk; GERA: https://www.ncbi.nlm.nih.gov/projects/gap/cgi-bin/study.cgi?study_id=phs000674.v2.p2; UKB GWAS summary data from the Neale Lab: http://www.nealelab.is/uk-biobank; baseline-LD annotations: https://data.broadinstitute.org/alkesgroup/LDSCORE; HapMap3: https://www.sanger.ac.uk/resources/downloads/human/hapmap3.html. Sparse LD matrix of ~1.1 million of HapMap3 SNPs computed from 50,000 unrelated UKB individuals of European ancestry: https://cnsgenomics.com/software/gctb/#Download.

Field-specific reporting

Please select the one below that is the best fit for your research. If you are not sure, read the appropriate sections before making your selection.

☒ Life sciences ☐ Behavioural & social sciences ☐ Ecological, evolutionary & environmental sciences

For a reference copy of the document with all sections, see nature.com/documents/nr-reporting-summary-flat.pdf

Life sciences study design

All studies must disclose on these points even when the disclosure is negative.

Sample size	We applied our methods to the GWAS data of 155 traits including diseases. The sample sizes for the traits analyzed in our study are shown in Supplementary Tables 1-3, which vary from ~36,000 to 550,000 with a median of ~350,000. These are the largest GWAS sample sizes to date for most of the traits. In most our analyses, we used a sample of ~350,000 unrelated UKB individuals of European ancestry, which is well-powered to precisely estimate all the genetic architecture parameters as demonstrated in Supplementary Figure 6.
Data exclusions	We restricted the GWAS analysis to unrelated individuals of European ancestry and SNPs with MAF > 0.01 (Methods), which are the commonly used criteria in previous studies of the genetic architecture of complex traits. Traits for which there was evidence for lack of convergence in MCMC were excluded (Methods), which is also a pre-established criterion.
Replication	No replication was performed because the aim of this study is to estimate the genetic architecture parameters with the highest possible precision by using the largest available sample sizes. Splitting the data into discovery and replication sets would reduce the precision of the estimates.
Randomization	Not applicable because the aim of the study is not to make causal inference.
Blinding	Not applicable because all the data used in this study have been collected previously.

Reporting for specific materials, systems and methods

We require information from authors about some types of materials, experimental systems and methods used in many studies. Here, indicate whether each material, system or method listed is relevant to your study. If you are not sure if a list item applies to your research, read the appropriate section before selecting a response.

Materials & experimental systems

n/a	Involved in the study
☒	☐ Antibodies
☒	☐ Eukaryotic cell lines
☒	☐ Palaeontology
☒	☐ Animals and other organisms
☒	☐ Human research participants
☒	☐ Clinical data

Methods

n/a	Involved in the study
☒	☐ ChIP-seq
☒	☐ Flow cytometry
☒	☐ MRI-based neuroimaging
